# HRK downregulation and augmented BCL-xL binding to BAK confer apoptotic protection to therapy-induced senescent melanoma cells

**DOI:** 10.1038/s41418-024-01417-z

**Published:** 2024-12-03

**Authors:** Clara Alcon, Marta Kovatcheva, Paula Morales-Sánchez, Vanessa López-Polo, Teresa Torres, Susana Puig, Albert Lu, Josep Samitier, Carlos Enrich, Manuel Serrano, Joan Montero

**Affiliations:** 1 Department of Biomedical Sciences, Faculty of Medicine and Health Sciences, University of Barcelona, Barcelona, Spain; 2 Institute for Research in Biomedicine (IRB Barcelona), Barcelona Institute of Science and Technology (BIST), Barcelona, Spain; 3 IFOM, The FIRC Institute of Molecular Oncology, Milan, Italy; 4 Institut d’Investigacions Biomèdiques August Pi i Sunyer (IDIBAPS), Barcelona, Spain; 5 CIBER of Rare Diseases (CIBERER), Instituto de Salud Carlos III, Barcelona, Spain; 6 Dermatology Department, Hospital Clinic and Fundació Clínic per la Recerca Biomèdica., Barcelona, Spain; 7 Institute for Bioengineering of Catalonia (IBEC), Barcelona Institute of Science and Technology (BIST), Barcelona, Spain; 8 Networking Biomedical Research Center in Bioengineering, Biomaterials and Nanomedicine (CIBER-BBN), Madrid, Spain; 9 Department of Electronics and Biomedical Engineering, Faculty of Physics, University of Barcelona, Barcelona, Spain; 10 Present Address: Altos Labs, Cambridge Institute of Science, Cambridge, UK

**Keywords:** Cancer, Cell biology

## Abstract

Senescent cells are commonly detected in tumors after chemo and radiotherapy, leading to a characteristic cellular phenotype that resists apoptotic cell death. In this study, we used multiple melanoma cell lines, molecular markers, and therapies to investigate the key role of the BCL-2 family proteins in the survival of senescent cells. We first used BH3 profiling to assess changes in apoptotic priming upon senescence induction. Unexpectedly, not all cell types analyzed showed a decrease in apoptotic priming, BIM was downregulated, there was variability in BAX expression and BAK remained constant or increased. Therefore, there was not a clear pattern for pro-survival adaptation. Many studies have been devoted to find ways to eliminate senescent cells, leading to one of the most studied senolytic agents: navitoclax, a promiscuous BH3 mimetic that inhibits BCL-2, BCL-xL and BCL-W. While it is known that the BCL-2 family of proteins is commonly upregulated in senescent cells, the complexity of the apoptotic network has not been fully explored. Interestingly, we found distinct protein expression changes always leading to a BCL-xL mediated pro-survival adaptation, as assessed by BH3 profiling. When analyzing potential therapeutic strategies, we observed a stronger senolytic activity in these melanoma cell lines when specifically targeting BCL-xL using A-1331852, navitoclax or the PROTAC BCL-xL degrader DT2216. We found that the sensitizer protein HRK was systematically downregulated when senescence was induced, leading to an increased availability of BCL-xL. Furthermore, we identified that the main apoptotic inhibition was shaped by BCL-xL and BAK binding increase that prevented mitochondrial permeabilization and apoptosis. To our knowledge, this is the first time that the molecular basis for BCL-xL anti-apoptotic adaptation in senescence is described, paving the way for the development of new molecules that either prevent HRK downregulation or displace BCL-xL binding to BAK to be used as senolytics.

## Introduction

Cellular senescence is a stable form of cell cycle exit that can be induced by a variety of stressors including aberrant oncogene activation, oxidative stress, and chemotherapies. While senescence is thought to have evolved as a tumor-suppressive mechanism [1], the inappropriate accumulation of senescent cells has pathogenic consequences, including risk of cancer relapse [2], contributing to the long-term side effects of cancer therapy [3], and acting as a driver of many age-associated illnesses, including fibrotic, cardiovascular, and neurologic diseases [4, 5]. A recent field of study between cancer and senescence that is now extensively explored is therapy-induced senescence (TIS). TIS has been found in tumors after radiation or chemotherapy, but only in a subset of cells [6]. Commonly used treatments such as doxorubicin, paclitaxel, cisplatin… conventional chemotherapy and radiotherapy in general, but also targeted therapies and immunotherapies have been reported to induce TIS [7, 8]. Thus, the selective elimination of senescent cells by a class of molecules known as senolytics has become a therapeutic objective [9, 10].

While the therapeutic potential of senolytics in a wide swath of pathologies is clear, the identification and successful administration of these drugs has been proved challenging. Navitoclax (ABT-263) was identified as one of the first senolytic agents based on the transcriptional profiling of senescent cells, which revealed a key role of anti-apoptotic BCL-2 family proteins [11, 12]. Resistance to apoptosis has become a so-called hallmark of senescent cells; paradoxically, the hypersensitivity of senescent cells to BH3 mimetic drugs indicates that they are simultaneously “primed” towards apoptosis [13, 14]. Rationally increasing this apoptotic priming and inducing killing in senescent cells is a goal of senolytic therapeutics.

While navitoclax is routinely used as a “gold standard” or proof of concept senolytic agent in preclinical studies, its therapeutic potential is confounded by dose-limiting toxicities largely based in thrombocytopenia. Navitoclax is a BH3 mimetic drug, whose mechanism of action is based on the inhibition of several anti-apoptotic BCL-2 family members, simultaneously targeting BCL-2, BCL-xL, and BCL-W. Platelet toxicity has been attributed to BCL-xL inhibition, which was followed by second generation BH3 mimetics with enhanced specificity, such as venetoclax (ABT-199), which specifically targets BCL-2 [15], or DT2216, a proteolysis targeting chimera (PROTAC) that targets BCL-xL without causing thrombocytopenia [16].

Nevertheless, when and how such drugs may have efficacy as senolytics is unclear. The pro- and anti-apoptotic network is complex, and the expression levels of its constituent genes do not necessarily reflect their cellular activity [17]. Moreover, recent work has indicated that different cell types exhibit unique adaptations, such as the MCL-1-dependent resistance to navitoclax in melanocytes [18]. Given the relatively low predictive capacity of individual features of the BCL-2 family members, a new generation of functional biomarkers has been recently established to predict anticancer therapy induction of apoptosis and guide the use of BH3 mimetics. Directly exposing living cancer cells to therapeutic agents ex vivo to determine chemosensitivity has been historically explored, but the development of novel technologies have fostered functional precision medicine. Among them, BH3 profiling has demonstrated that it can rapidly identify anti-apoptotic dependencies in cancer cells [19, 20]. This functional assay uses synthetic ~ 20-mer BH3 peptides, mimicking BH3-only proteins, acting as a pro-death signal to induce mitochondrial outer membrane permeabilization (MOMP). By using different peptides, BH3 profiling can rapidly interrogate cancer cells and obtain precise information regarding the apoptotic status of the cell. For example, the BH3 peptides BIM and BID, that bind to all anti-apoptotic proteins and directly activate the effector proteins BAX/BAK and MOMP, measure overall apoptotic priming, or how close cancer cells are to the apoptotic threshold [21, 22]. Further development of this technique led to dynamic BH3 profiling, which rapidly measures how much a given treatment primes cancer cells towards apoptosis (Δ% priming) by uniquely measuring early changes in the apoptotic signaling preceding frank cell death on the order of days or weeks [23]. In addition, by using peptides recapitulating the BH3 domain sequence of sensitizer proteins—such as BAD, HRK, or NOXA BH3 peptides— BH3 profiling can also identify changes in anti-apoptotic dependencies upon treatment and accurately identify BH3 mimetic combinations to enhance cytotoxicity [20, 24–26], also to eliminate oncogene-induced senescent cells [27]. Nevertheless, to date BH3 profiling has not been fully validated on senescent cells. Given the hypersensitivity of senescent cells to BH3 mimetics, we reasoned that BH3 profiling could be a novel approach to dissect the complexity of anti-apoptotic networks in senescent cells, and with which to nominate novel senolytic therapeutic approaches.

## Results

### Palbociclib-induced senescence distinctly affects apoptotic signaling in melanoma cells

**Fig. 1:**
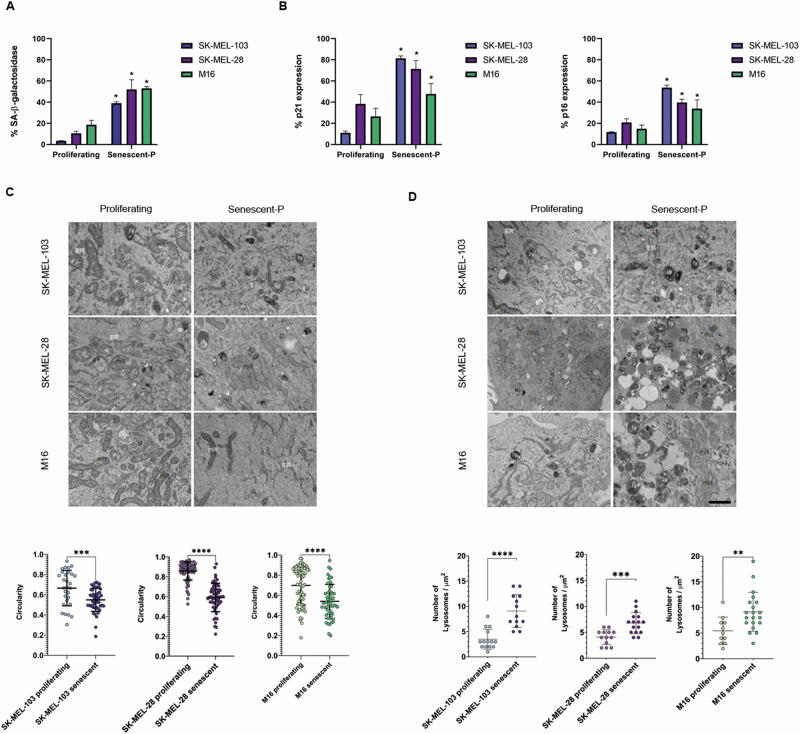
Characterization of palbociclib-induced senescent melanoma cells. **A** Quantification of SA-β-galactosidase in proliferating and palbociclib-induced senescent melanoma cells (senescent-P) by flow cytometry. **B** % of p21 and p16 in proliferating and palbociclib-induced senescent (senescent-P) melanoma cells measured by flow cytometry. **C**, **D** Upper panels: Representative images of transmission electron microscopy from proliferating and palbociclib-induced senescent (senescent-P) melanoma cells. ER endoplasmic reticulum, ly lysosome, mit mitochondria, LD lipid droplet, g Golgi complex, Nu nucleus. Scale bar: 1 μm. Lower panels: Quantification of mitochondrial circularity and the number of lysosomes in proliferating and palbociclib-induced senescent melanoma cells. Values indicate mean values ± SEM from at least three independent experiments. ****p < 0.0001, *** *p* < 0.001, **p < 0.01, *p < 0.05 compared to control.

We decided to focus in melanoma since senescence and apoptotic protection play a key role in this type of cancer development and therapy outcome [18, 24, 28]. We used three melanoma cell lines; SK-MEL-103 (NRAS mutant), SK-MEL-28 (BRAFV600E mutant) [28], and the M16 (BRAFV600E mutant) that was derived from a patient continuously exposed to UV light, as previously described [29]. To induce senescence, we chronically treated these cell lines with a CDK4/6 inhibitor for 7 days [8] and, since this inhibitor does not uniformly induce senescence in all cancer cells [30], we confirmed the phenotype using different well-established markers: SA-β-galactosidase, p21, and p16 [31, 32]. As expected, we detected a significant increase of these markers in all three cell lines when treated with palbociclib, thus indicating therapy-induced senescence (TIS) (Fig. 1A, B). To assess senescence morphologically, we examined by transmission electron microscopy (TEM) for potential phenotypic changes. Overall, we detected elongated mitochondria as measured by a decrease in their circularity (Fig. 1C) and an increase in the number of lysosomes (Fig. 1D) in palbociclib-treated cells, as previously described [5, 33]. All these observations confirm that palbociclib effectively induces senescence in all three melanoma cell lines examined.

**Fig. 2:**
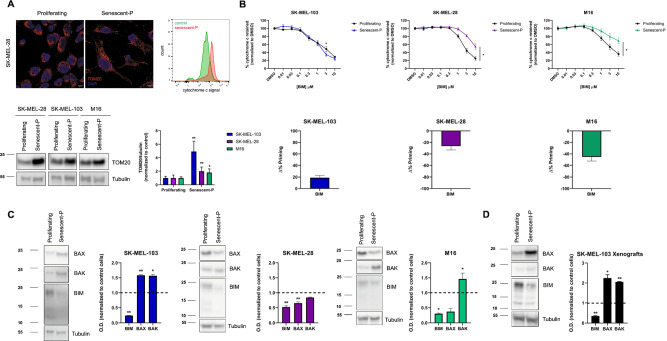
Palbociclib-induced senescence distinctly affects apoptotic priming and protein expression in melanoma cells. **A** Upper left panel: Confocal images of proliferating and palbociclib-induced senescent SK-MEL-28 cells (senescent-P) stained with the mitochondrial marker TOM20 (red) and DAPI (blue). Upper right panel: Representative histograms of cytochrome c signal detected by flow cytometry in proliferating and palbociclib-induced senescent (senescent-P) SK-MEL-28 cells. Lower panel: Representative western blot images and optical density quantification for TOM20 and tubulin of proliferating and palbociclib-induced senescent SK-MEL-103, SK-MEL-28 and M16 cells. **B** Upper panel: % of cytochrome c retained inside the mitochondria with increasing concentrations of BIM peptide in control and palbociclib-induced senescent SK-MEL-103, SK-MEL-28, and M16 cells (senescent-P). Lower panel: Maximum Δ% priming BIM peptide in the three palbociclib-induced cell lines. **C** Representative western blot images and quantification of the optical density of each protein and normalized with tubulin in the three cell lines. **D** Representative western blot images and quantification of the optical density of each protein and normalized with tubulin in protein extracts from SK-MEL-103 tumor xenografts. Results expressed as fold increase represent the increase in optical density compared to proliferating cells. Values indicate mean values ± SEM from at least three independent experiments. **p < 0.01, *p < 0.05 compared to control.

Senescent cells often present resistance to die by apoptosis [2]. Therefore, we next sought to study how TIS modulates apoptosis using BH3 profiling. As mentioned above, senescent cells present longer mitochondria, and we reasoned that this could affect MOMP and cytochrome c release measurements, which are required for BH3 profiling analyses [22–24]. Thus, we first evaluated if these selected senescent melanoma cells presented a larger mitochondrial content compared to non-senescent cells. Using TOM20 as a mitochondrial marker, we observed a significant increase in its expression in palbociclib-induced senescent SK-MEL-28, SK-MEL-103 and M16 cells compared to non-treated (proliferating) cells (Fig. 2A). We also evaluated cytochrome c staining by flow cytometry and, correlating with TEM, fluorescence microscopy and western blot, we detected a significant increase in its intensity after senescence induction (Fig. 2A). Therefore, for flow cytometry BH3 profiling analyses we adjusted the cytochrome c gating taking into consideration this variation in basal fluorescence (see the “Methods” section for more details). When comparing non-treated (proliferating) and palbociclib-treated melanoma cancer cells by BH3 profiling, we observed that these cell lines showed a different priming pattern when becoming senescent. If a given treatment or state sensitizes cells towards apoptosis, this would cause a leftward shift of the cytochrome c retained curve after BIM peptide exposure, since less amount of BIM is required to promote MOMP and cytochrome c release, indicating an increase in overall apoptotic priming (positive Δ% priming). In contrast, if a treatment or state renders cells more resistant to apoptosis, we would observe a shift of the curve to the right, indicating a decrease in priming (negative Δ% priming). Surprisingly, we observed that SK-MEL-103 cells treated with palbociclib became slightly primed for apoptosis, while the SK-MEL-28 and M16 cells clearly turn out to be less primed for apoptosis (Fig. 2B).

To further elucidate this distinct effect on apoptotic priming induced by palbociclib, we compared the expression of different pro-apoptotic members of the BCL-2 family of proteins between proliferating and senescent cells. For instance, in all cell lines we observed a significant reduction of the activator protein BIM (Fig. 2C), that could partially explain why senescent cells exert resistance to cell death. When further analyzing the SK-MEL-103 cells, we identified that the expression of the effector proteins BAX and BAK significantly increased after palbociclib treatment, correlating with the observed increase in Δ% priming detected by BH3 profiling with the BIM BH3 peptide (Fig. 2B). In fact, these results correlate with previous mRNA expression data results from the Serrano laboratory [34] (Supplementary Fig. 1). However, in SK-MEL-28 and M16 cells we observed that BAK remained constant or increased while BAX expression decreased, partially explaining the potential role of the latter regulating negative Δ% priming detected by BH3 profiling (Fig. 2C). Furthermore, we assessed tumors from SK-MEL-103 xenografts treated with palbociclib [35], which became senescent (Supplementary Fig. 2), and detected a decrease in BIM and an increase in BAK (and BAX) expression, correlating with our in vitro observations (Fig. 2D).

In summary, palbociclib promoted a decrease in the activator protein BIM, thus preventing apoptosis, and effectively inducing senescence, assessed by SA-β-galactosidase, p21 and p16. Paradoxically, one melanoma cell line became slightly primed for apoptosis (positive Δ% priming) while the other two showed protection towards this form of programmed cell death (negative Δ% priming) in part due to a distinct change in BAX expression after TIS.

### Therapy-induced senescence induces a BCL-xL-dependent anti-apoptotic adaptation and can be targeted with specific BH3 mimetics or PROTAC

**Fig. 3:**
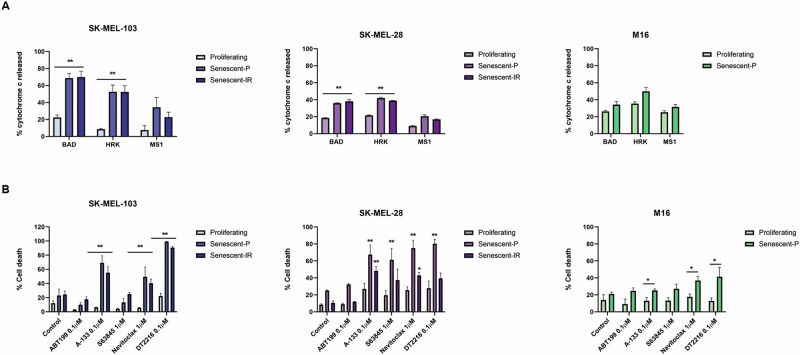
Senescent melanoma cells acquire BCL-xL dependence. **A** % cytochrome c release after the incubation with sensitizer peptides (BAD 10 µM, HRK 100 µM, and MS1 10 µM) in proliferating, palbociclib-induced (senescent-P) and irradiation-induced senescent (senescent-IR) melanoma cells measured by flow cytometry. **B** % cell death assessed by annexin V and DAPI staining and flow cytometry analysis in proliferating, palbociclib-induced (senescent-P) and irradiated-induced senescent (senescent-IR) melanoma cells after the incubation with the treatments for 48 h in SK-MEL-103 and SK-MEL-28 and 96 h in M16 cells. Values indicate mean values ± SEM from at least three independent experiments. **p < 0.01, *p < 0.05 compared to control.

Another hallmark of senescent cells is their pro-survival adaptation through anti-apoptotic BCL-2 family proteins, and non-surprisingly one of the most studied senolytics has been the promiscuous BCL-2/BCL-xL/BCL-W inhibitor navitoclax [11]. We sought to elucidate if TIS melanoma cells also presented a similar adaptation and, if that was the case, how they prevented apoptosis. Thus, we performed BH3 profiling using specific BH3 peptides BAD (for BCL-2 and BCL-xL), HRK (for BCL-xL) and MS1 (for MCL-1), as previously described [24, 25], on the same cell lines after senescence induction using palbociclib, and γ-irradiation (which also increased SA-β-galactosidase activity, p21 and p16 expression in all three cell lines as shown in Supplementary Fig. 3). In brief, a positive Δ% priming using these sensitizer BH3 peptides would point to a specific anti-apoptotic protein that senescent cells become addicted to. We found that SK-MEL-103, SK-MEL-28 and, to a lesser extent, M16 displayed a significant BCL-xL adaptation, as we detected an increase in the % of cytochrome c released with the HRK (and BAD, that binds to BCL-2 and BCL-xL) peptide (Fig. 3A). We also detected a minor increase in priming with the MS1 BH3 peptide, indicating a partial adaptation through MCL-1 but to a lesser extent than BCL-xL (Fig. 3A). We next explored anti-apoptotic inhibition potential to eliminate senescent cells. Using palbociclib and γ-irradiation, we generated senescent melanoma cells and tested BH3 mimetics’ senolytic activity. We exposed these cells to ABT-199 (venetoclax), the BCL-xL inhibitor A-1331852, the MCL-1 inhibitor S63845, ABT-263 (navitoclax) and the BCL-xL degrading PROTAC DT2216 [16]. As anticipated by our BH3 profiling results, we observed significant senolytic activity in these melanoma cell lines when targeting BCL-xL using A-1331852, navitoclax or DT2216 (Fig. 3B); and in SK-MEL-103 and SK-MEL-28 BCL-xL targeting therapies had a greater senolytic activity compared to M16. Furthermore, we detected a significant increase in cell death with the MCL-1 inhibitor S63845 in SK-MEL-28 suggesting an implication of this protein in apoptotic resistance in senescent cells harboring a BRAFV600 mutation [24]. These results indicate that TIS melanoma cells block apoptosis primarily through BCL-xL, and to a lesser extent using MCL-1. Consequently, inhibiting this anti-apoptotic protein with specific inhibitors - beyond navitoclax that also targets BCL-2 and BCL-W - such as the BH3 mimetic A-1331852 or a PROTAC like DT2216 that would not cause thrombocytopenia, due to the minor expression of E3 ligase in platelets [16], appears as an effective therapeutic strategy to remove melanoma senescent cells.

### BCL-xL adaptation is mediated by HRK downregulation and changes in binding affinities to pro-apoptotic proteins

**Fig. 4:**
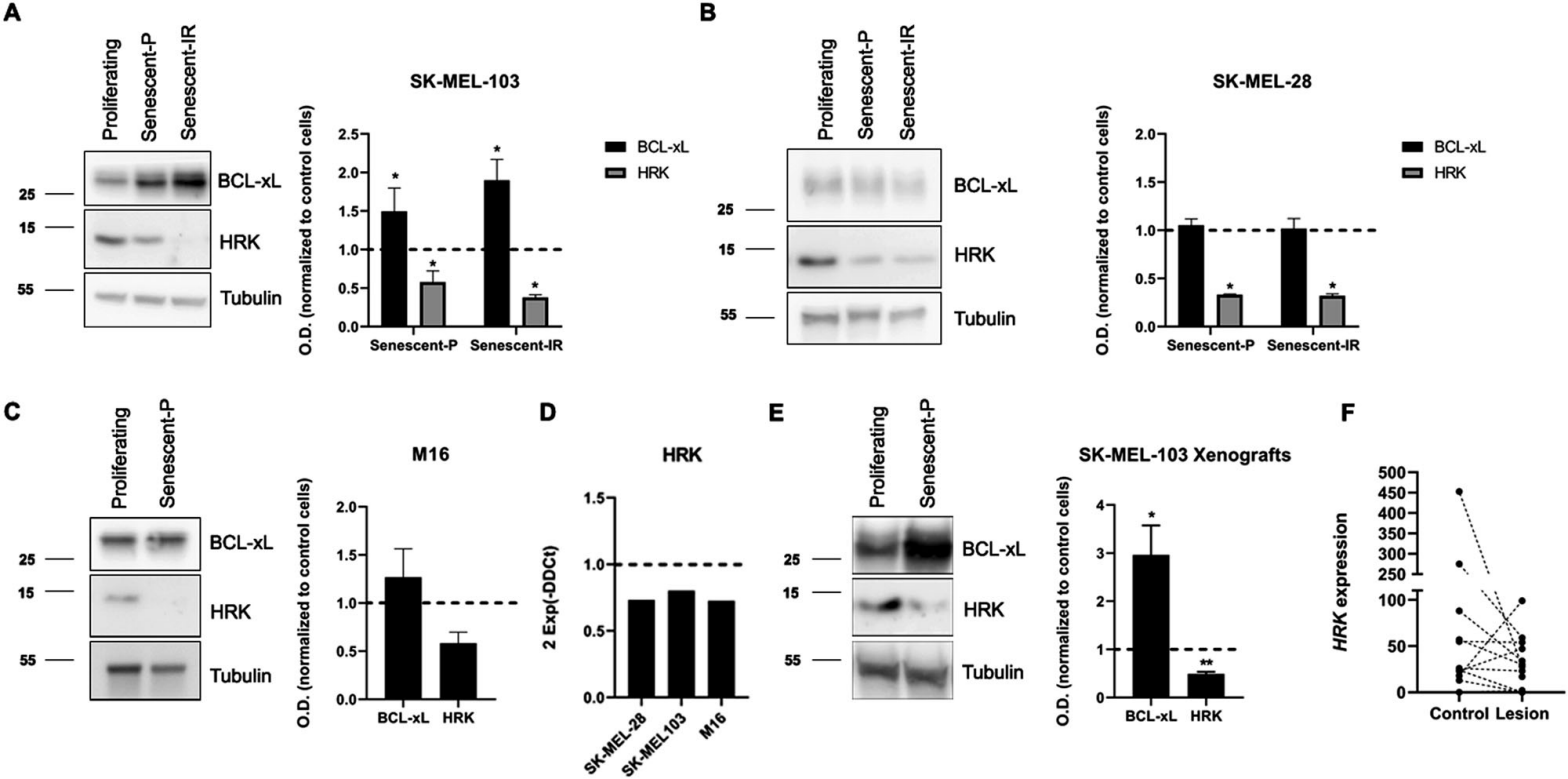
BCL-xL dependence in senescent melanoma cells is mediated through HRK downregulation. **A** Representative western blot images and optical density quantification for BCL-xL and HRK of proliferating, palbociclib-induced (senescent-P) and irradiated-induced senescent (senescent-IR) SK-MEL-103 cells. **B** Representative western blot images and optical density quantification for BCL-xL and HRK of proliferating, palbociclib-induced (senescent-P) and irradiated-induced senescent (senescent-IR) SK-MEL-28 cells. **C** Representative western blot images and optical density quantification for BCL-xL and HRK of proliferating and palbociclib-induced senescent M16 cells (senescent-P). Results expressed as fold increase represents the increase in optical density compared to proliferating cells. **D** RT-qPCR results showing a decrease in mRNA expression of *HRK* gene in palbociblib-induced melanoma senescent cells. **E** Representative western blot images and optical density quantification for BCL-xL and HRK of protein extracts from SK-MEL-103 control and palbociclib-treated xenografts. **F** mRNA expression of *HRK* in control and lesioned skin from healthy donors. Values indicate mean values ± SEM from at least three independent experiments. **p < 0.01, *p < 0.05 com*p*ared to control.

To elucidate how TIS melanoma cells become dependent on BCL-xL, we first analyzed the expression of this anti-apoptotic protein. We found in palbociclib-treated SK-MEL-103 and M16 cells that its expression increased. Surprisingly, SK-MEL-28 cells did not exert significant BCL-xL changes upon treatment, suggesting a potential role of another BCL-2 family member to regulate its availability (Fig. 4A–C). In this regard, we analyzed several BCL-2 family proteins, and found that the sensitizer HRK, which specifically binds to BCL-xL [36], was clearly downregulated in all three cell lines when induced into senescence (Fig. 4A–C). This downregulation observed in cell lines could be partially explained by a decrease in gene transcription (Fig. 4D). Furthermore, SK-MEL-103 xenografts treated with palbociclib also experienced a decrease in HRK protein expression (Fig. 4E). In other words, senescent inducers such as palbociclib and γ-irradiation decreased the expression of HRK, initially bound to BCL-xL, thus liberating this pro-survival protein to protect cells towards apoptosis. Similar results were observed in healthy donors comparing control skin with UV irradiated (Table 1), where the latter showed an increase trend in the senescence markers *CDKN1A* (p21) and *CDKN2A* (p16) (Supplementary Fig. 4), and a decrease trend in *HRK* mRNA expression (Fig. 4F) pointing to a potential BCL-xL adaptation in senescent skin from patients. Furthermore, knocking down HRK using siRNA in SK-MEL-28 and SK-MEL-103 promoted a similar effect regarding apoptotic priming and BCL-xL dependence as palbociclib-induced senescent cells (Supplementary Fig. 5A–C).

**Table 1:** Description of subjects included in the analysis shown in Fig. 4D and their main characteristics.

Volunteer	Age	Phototype	Sex
P002	57	2	Male
P003	55	3	Male
P005	49	3	Female
P006	52	3	Female
P007	50	3	Female
P013	47	2	Male
P014	41	3	Female
P015	54	3	Female
P016	46	3	Female
P017	52	2	Female
P018	50	3	Male
P020	66	3	Female
P021	60	2	Female
P022	58	3	Female

**Fig. 5:**
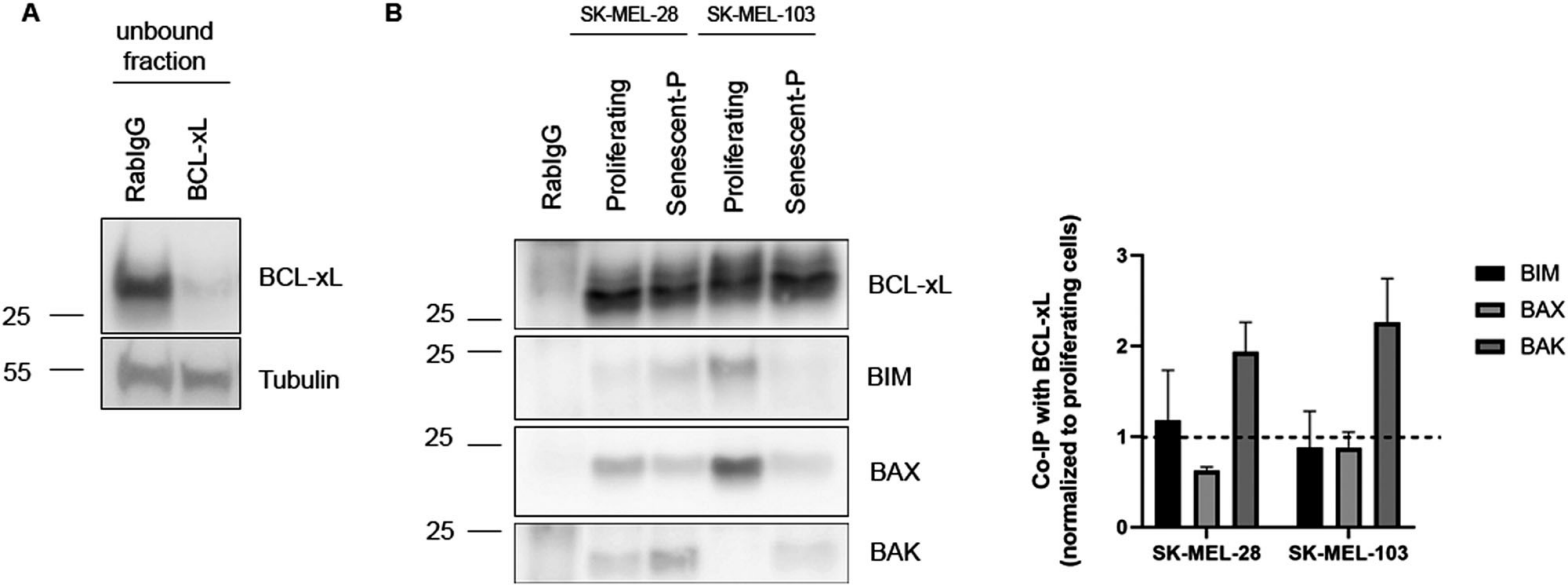
BCL-xL presents higher binding affinity for BAK in senescent melanoma cells. **A** Representative western blot image from unbound fractions after RabIgG or BCL-xL immunoprecipitation. **B** Representative western blot images from the co-immunoprecipitation of BCL-xL with BAK, BIM and BAX in proliferating and palbociclib-induced senescent (senescent-P) SK-MEL-28 and SK-MEL-103 cell lysates. Values indicate mean values ± SEM from at least three independent experiments.

We previously showed that palbociclib treatment promoted a decrease in BIM, HRK and, in some cases, BAX expression, consequently preventing apoptosis. So, our next question was how BCL-xL inhibitors restored apoptotic cell death in senescent cells. The BCL-2 family of proteins represents a complex interactome regulated at multiple levels, including protein expression, posttranslational modifications, and dynamic binding affinities. To further elucidate the role of BCL-xL in TIS, we immunoprecipitated this anti-apoptotic protein with a high efficiency (Fig. 5A) and analyzed its binding to BIM, BAX and BAK in SK-MEL-103 and SK-MEL-28. As shown in Fig. 2, both cell lines exerted distinct BAX/BAK expression changes when senescence was induced with palbociclib. However, in our immunoprecipitation analyses we observed a clear increase in binding between BCL-xL and BAK in senescent cells compared to control cells, while it decreased or remained similar for the other two proteins (Fig. 5B). Similar results were also observed in SK-MEL-28 and SK-MEL-103 cells transfected with siRNA against HRK (Supplementary Fig. 5D). In summary, we describe for the first time that palbociclib-induced senescence promotes different changes in the BCL-2 family proteins’ expression that lead to an increase in BCL-xL:BAK binding that prevents apoptosis and explains why specifically BCL-xL targeted agents are effective senolytics.

## Discussion

One of the hallmarks of senescent cells is their resistance to die by apoptosis [2]. Consequently, our first aim was to study if senescent melanoma cells showed this resistance and specifically how apoptotic cell death was affected. We used three different melanoma cell lines: SK-MEL-103 that presents a mutation in NRAS, and SK-MEL-28 and M16 that have a BRAFV600E mutation [28, 29]. We exposed all three cell types to different well-established senescence inducing factors, palbociclib and γ-irradiation, and positively detected an increase in p16, p21 and SA-β-galactosidase in all cases (Fig. 1A-B and Supplementary Fig. 3). We confirmed senescence induction by electron microscopy where, beyond the increase in cellular size, we identified two commonly observed morphological changes: mitochondria elongation (Figs. 1C, 2A) and an increment in the number of lysosomes (Fig. 1D) [33, 34]. Since mitochondrial permeabilization is a key step in apoptosis induction and could be altered in senescent cells, we next evaluated potential changes in mitochondrial polarization and cytochrome c expression, and we clearly observed by confocal microscopy and flow cytometry an increased intensity on the latter with senescence (Fig. 2A). Consequently, we had to adjust our flow cytometry settings when performing the BH3 profiling analyses on these cells [22, 24, 25]. Interestingly, we observed a different BH3 profiling trend when comparing the NRAS mutant cell line SK-MEL-103 with the BRAF-mutant SK-MEL-28 and M16 cells. When measuring overall priming with the BIM BH3 peptide [14], we observed that senescent SK-MEL-103 cells became slightly primed to apoptosis while SK-MEL-28 and M16 were less primed after palbociclib treatment. When we analyzed the expression of different pro-apoptotic BCL-2 family proteins, we detected that in all cases senescence induction caused a decrease in BIM (that would have a protective effect towards apoptosis), that BAK remained unchanged or increased, and that BAX decreased in the BRAF-mutant cells but increased in SK-MEL-103 (Fig. 2C). Precisely the distinct changes in BAX expression between these melanoma cell lines, could partially explain the differences observed in BH3 profiling overall apoptotic priming with senescence. In summary, the studied melanoma cell lines presented variability in BAX protein expression and overall apoptotic priming when becoming senescent but in all cases, BIM was downregulated and importantly BAK expression remained constant or increased.

The cellular process of senescence has been related to ageing [5] and cancer cells’ survival to therapy [3, 37]. However, how therapy-induced senescent cells avoid apoptosis is not fully understood. Several studies previously described that senescent cells utilize anti-apoptotic BCL-2 family proteins to resist cell death and, because of that, they are particularly sensitive to BH3 mimetics such as navitoclax, that blocks BCL-2, BCL-xL, and BCL-W [7, 11]. Thus, we next sought to further understand how this anti-apoptotic adaptation occurs using BH3 profiling [14, 20, 24–26]. We identified that when exposed to palbociclib or γ-irradiation, melanoma cells clearly became BCL-xL dependent (Fig. 3A). In other words, TIS in these cells promoted a pro-survival adaptation mostly through BCL-xL to prevent cell death. Other anti-apoptotic proteins such as MCL-1 were identified by BH3 profiling but to a lesser extent, indicating a secondary role in TIS pro-survival adaptation. We next used different anti-apoptotic inhibitors or degraders as senolytics to test if we could specifically eliminate these senescent melanoma cells. As anticipated by BH3 profiling, those molecules that specifically inhibited BCL-xL (A-1331852 and navitoclax) or the PROTAC DT2216 showed a significant cytotoxic effect on senescent cells in all cases (Fig. 3B). Other BH3 mimetics directed towards BCL-2 or specially MCL-1 had a minor effect eliminating these cells. These results suggest that despite the observed variability in overall apoptotic priming and BCL-2 family protein expression (Fig. 2), TIS in melanoma leads to a marked BCL-xL dependence in all cases, and its inhibition or degradation efficiently eliminates senescent cells.

**Fig. 6:**
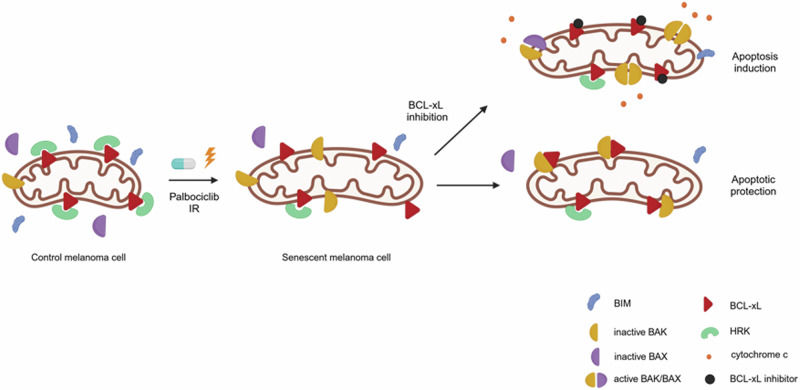
Mechanism of apoptotic protection in senescent melanoma cells. Schematic representation of BCL-2 family proteins interaction in senescent melanoma cells. Therapy-induced senescence with palbociclib or γ-irradiation reduces HRK levels which allow the binding of available BCL-xL to BAK and confers protection against apoptosis induction. When BCL-xL is targeted, BAK could be displaced and apoptosis could be restored.

After identifying the key role of BCL-xL in the survival of melanoma cells upon senescence induction, we next sought to study how precisely this anti-apoptotic protein impedes cell death. We first analyzed its expression when TIS was induced and observed that it increased in SK-MEL-103 and M16, or remained unchanged in SK-MEL-28 (Fig. 4A–C). We were particularly intrigued by the latter since in these cells we also observed a BCL-xL adaptation. We then analyzed other BCL-2 family members, particularly the sensitizer proteins, and found that HRK, that specifically binds and blocks BCL-xL [36], protein expression decreased with TIS in all cases. This protein downregulation could be partially explained by a decrease in transcription as observed by RT-qPCR analyses in all three cell lines (Fig. 4D). Furthermore, we observed in SK-MEL-103 xenografts treated with palbociclib (Fig. 4E) and in photodamaged skin from donors that senescence markers tend to increase while HRK expression tends to decrease (Fig. 4F), correlating with our in vitro observations. In other words, senescence induction in cell lines, tumor xenografts and in UV-damaged skin from donors, promotes a downregulation of HRK that frees BCL-xL to block MOMP and apoptotic cell death (Fig. 6). But at that point a key question was still unanswered: which specific pro-apoptotic proteins were inhibited by BCL-xL and how was mitochondrial permeabilization prevented? To answer this question, we immunoprecipitated BCL-xL and analyzed by western blot which proteins were bound to it. Importantly, we found in SK-MEL-103 and SK-MEL-28 cells that TIS promoted an increase in BAK binding to BCL-xL that blocked MOMP and apoptosis (Figs. 5 and 6).

In summary, TIS is emerging as a key determinant of the survival of cancer cells after anticancer treatment and could explain why some melanoma patients relapse. Senescence has been extensively studied, in part because of its implications in age-related diseases, and navitoclax has been for years one of the most studied senolytics through its capacity to simultaneously inhibit three important anti-apoptotic proteins: BCL-2, BCL-xL and BCL-W [5, 11, 38]. Nevertheless, there is emerging evidence that cell-type-specific features contribute to the efficacy and specificity of senolytic treatments, and that navitoclax is not effective in all cases [3, 18, 39]. Thus, a mechanistic and functional understanding of the anti-apoptotic network in senescent cells will be necessary to effectively include senolytic agents in treatment regimens. In this study, we used a panel of melanoma cell lines to demonstrate the value of BH3 profiling to uncover specific senolytic vulnerabilities. We here report that TIS in melanoma promoted a BCL-xL mediated pro-survival adaptation– distinct from what has been reported in untransformed melanocytes [29]—that could be prevented by using specific inhibitors (also navitoclax) or a PROTAC degrader against BCL-xL. We also showed that targeting other anti-apoptotic proteins such as BCL-2, BCL-W or MCL-1 had a minor senolytic effect, and its concomitant inhibition would only lead to undesired toxicities. We further investigated how this adaptation occurred and found that TIS promoted a consistent decrease of the sensitizer protein HRK that liberated BCL-xL, and that the latter specifically blocked the effector BCL-2 family protein BAK to prevent MOMP and apoptosis. To our knowledge this is the first time that this senescence pro-survival mechanism is described and could help develop new effective senolytics directed to avoid HRK downregulation or disrupt the BCL-xL:BAK binding. We believe that this new senolytic strategy guided by BH3 profiling could prevent cancer therapy endurance and potentially avoid some melanoma relapse cases in the future.

## Materials and methods

### Cell lines and treatments

Melanoma cell line SK-MEL-28 was purchased at ATCC (ATCC^®^ CRL-1772^™^, ATCC, Manassas, VI, USA) and SK-MEL-103 cell line was provided by Dr. Marisol Soengas. Patient-derived cell line M16 was kindly provided by Dr. Susana Puig from Hospital Clínic de Barcelona. SK-MEL-28 cell line was cultured in RPMI 1640 medium (31870, Thermo Fisher, Gibco, Paisley, Scotland) supplemented with 10% heat-inactivated fetal bovine serum (10270, Thermo Fisher, Gibco), 1% of l-glutamine (25030, Thermo Fisher, Gibco), and 1% of penicillin and streptomycin (15140, Thermo Fisher, Gibco). SK-MEL-103 and M16 cells were cultured in DMEM high glucose medium (41965, Thermo Fisher, Gibco) supplemented with 10% heat-inactivated fetal bovine serum, 1% of penicillin and streptomycin and 1% of l-glutamine. All cells were tested for mycoplasma and maintained at 37 °C in a humidified atmosphere of 5% CO_2_.

BH3 mimetics ABT-199 (venetoclax), A-1331852 (A-133), S63845 and ABT-263 (navitoclax) were purchased at Selleckchem (Munich, Germany) and the PROTAC BCL-xL degrader DT2216 was purchased at AbMole BioScience (Houston, Texas, USA). Treatments were performed directly in the culture media at the indicated concentrations.

### Palbociclib-induced senescence

For palbociclib-induced senescence, cells were seeded in a six-well plate and treated with palbociclib (S1116, Selleckchem) at 1 µM dose. After 3 days cells were replenished with fresh media and palbociclib. Seven days after the treatment, senescent cells were used for further analysis.

### Irradiation-induced senescence

For irradiated-induced senescence, cells were seeded in a six-well plate and irradiated with a gamma irradiator MARK I 30 (J.L. Shepherd & Associates, San Fernando, CA, USA) from the Scientific and Technological Centers of the University of Barcelona at 10 Gy. After 3 days, the media was changed and cells were maintained in culture till day 7 after irradiation and used for further analysis.

### Senescence associated β-galactosidase staining

Cells were stained using CellEvent™ Senescence Green Detection Kit (C10851, Thermo Fisher). Briefly, cells were washed with phosphate buffer saline (PBS), tripsinized, fixed with 4% formaldehyde during 10 min at room temperature, washed with PBS + 1% BSA and incubated with the working solution at 37 °C without CO_2_ during 2 h. After incubation, cells were washed with PBS and then imaged using a spectral flow cytometry Cytek AURORA instrument (Cytek Biosciences, California, USA) from the Scientific and Technological Centers of the University of Barcelona.

### BH3 profiling

BH3 profiling was developed at the Letai laboratory, and the experiments were performed as previously described [20, 22, 25]. After inducing senescence for 7 days cells were stained with the viability marker Zombie Violet (423113, BioLegend, Koblenz, Germany) and then washed with PBS and resuspended in MEB (150 mM mannitol, 10 mM hepes–KOH pH 7.5, 150 mM KCl, 1 mM EGTA, 1 mM EDTA, 0.1% BSA, 5 mM succinate) in a final volume of 25 μL. Afterwards, cells were exposed to the following peptides: 25 μL of BIM BH3 peptide (final concentration of 0.01, 0.03, 0.1, 0.3, 1, 3, and 10 μM), 25 μL of BAD BH3 peptide (final concentration of 10 μM), 25 μL of HRK BH3 peptide (final concentration of 100 μM), and 25 μL of MS1 BH3 peptide (final concentration of 10 μM) in MEB with 0.002% digitonin in a 96-well plate (3795, Corning, Madrid, Spain) for 1 h following fixation with formaldehyde. Cells were finally stained with cytochrome c antibody (Alexa Fluor® 647 anti-Cytochrome c—6H2.B4, 612310, BioLegend), p21 antibody (sc-6246 PE, Santa Cruz Biotechnology, Texas, USA) and p16 antibody (Alexa Fluor® 488 CDKN2A/p16INK4a, bs-4592R-A488, Bioss Antibodies, Woburn, Massachusetts, USA). Individual BH3 profiling analyses were performed using triplicates for DMSO, alamethecin (BML-A150-0005, Enzo Life Sciences, Lorrach, Germany), the different BIM BH3 concentrations used, BAD, HRK, and MS1 BH3 peptides. The expressed values stand for the average of three different readings performed with a high-throughput spectral flow cytometry Cytek AURORA instrument (Cytek Biosciences) from the Scientific and Technological Centers of the University of Barcelona. % of cytochrome c released is used to calculate Δ% priming which represents the difference between treated cells minus non-treated cells for a given peptide.

### Flow cytometry analysis

Due to the difference of mitochondrial content between proliferating and senescence cells and therefore a major cytochrome c retained signaling was detected in senescent cells versus control cells, we adjusted the gating for cytochrome c retention with the control condition of each experimental setting (proliferating and senescent). Analyses were performed using FlowJo software.

### Cell death assay

Cells were trypsinized and stained with fluorescent conjugates of Annexin V (Alexa Fluor® 647 Annexin V, 640912, BioLegend) and DAPI (62248, Thermo Fisher) and analyzed on a flow cytometry Gallios instrument (Beckman Coulter, Nyon, Switzerland) from the Scientific and Technological Centers of the University of Barcelona. Viable cells are Annexin V DAPI negative, and cell death is expressed as 100%-viable cells.

### Protein extraction and quantification

Cells were lysed using RIPA buffer (150 mM NaCl, 5 mM EDTA, 50 mM Tris–HCl pH = 8, 1% Triton X-100, 0.1% SDS, EDTA-free Protease Inhibitor Cocktail (4693159001 Roche, Mannkin, Germany)) for 30 min at 4 °C followed by a centrifugation at 16,100 × *g* for 10 min. Frozen tissue from SK-MEL-103 xenografts was homogenized in RIPA buffer using a tissue homogenizer and centrifuged at 16,100 × *g* for 10 min. The supernatant was stored at −20 °C for protein quantification performed using PierceTM BCA Protein Assay Kit (23227, Thermo Fisher).

### Immunoprecipitation

Cells were lysed using Immunoprecipitation buffer (150 mM NaCl, 10 mM Hepes, 2 mM EDTA, 1% Triton, 1.5 mM MgCl_2_, 10% glycerol, and EDTA-free Protease Inhibitor Cocktail (4693159001 Roche)) and centrifuged at 16,100 × *g*, 15 min at 4 °C. Supernatants were incubated with magnetic beads (Dynabeads 10003D, Thermo Fisher) conjugated to 5 µg of rabbit anti-BCL-xL antibody (CST2764, Cell Signaling, Leiden, The Netherlands) or 5 μg of rabbit IgG antibody (CST2729, Cell Signaling) at 4 °C overnight. After magnetization, a part of the supernatant was mixed with half volume of 4× SDS–PAGE sample buffer, heated at 96 °C for 5 min and stored at −80 °C as unbound fractions. The rest of the supernatant was discarded. The resulting pellet was washed and mixed with 60 µL 4× SDS–PAGE sample buffer and heated for 10 min at 70 °C followed by magnetization and collection of the supernatant for further immunoblotting analyses.

### Immunoblotting

Proteins were separated by SDS–PAGE (Mini-Protean TGX Precast Gel 12%, 456–1045, Bio-Rad, Hercules, California, USA) and transferred to PVDF membranes (10600023, Amersham Hybond, Pittsburgh, PA, USA). Membranes were blocked with dry milk dissolved in Tris buffer saline with 1% Tween 20 (TBST) for 1 h and probed overnight at 4 °C with the primary antibodies of interest directed against: rabbit anti-BCL-xL (CST2764, Cell Signaling), rabbit anti-BIM (CST2933, Cell Signaling), rabbit anti-BAK (CST12105, Cell Signaling), rabbit anti-BAX (CST2772, Cell Signaling), rabbit anti-HRK (PRS3771, Sigma-Aldrich, San Luis, Missouri, USA), mouse anti-γ−tubulin (T6557, Sigma-Aldrich), rabbit anti-Tom20 (ab186734, Abcam, Cambridge, UK) followed by anti-rabbit or anti-mouse IgG HRP-linked secondary antibody (CST7074 or CST7076 Cell Signaling) in 3% BSA in TBST for 1 h at room temperature. Immunoblots were developed using Clarity ECL Western substrate (1705060, Bio-Rad), visualized with LAS4000 imager (GE Healthcare Bio-Sciences AB, Uppsala, Sweden) and ImageJ was then used to measure the integrated optical density of bands.

### Transmission electron microscopy procedure for flat embedding preparation of cell monolayers

For transmission electron microscopy (TEM) preparation and analysis of melanoma cells (proliferating and senescence) growing on coverslips, the cells were first washed with 0.1 M phosphate buffer (PB) to remove the excess of culture medium and fixed with a freshly made 3% glutaraldehyde solution in 0.1 M PB for 1 h at room temperature. Subsequently, the fixative was removed, and the samples were maintained at 4 °C in a fresh fixative solution until processing.

The post-fixation procedure was conducted using 1% OsO_4_ for a period of 90 min at 4 °C. Then, samples were dehydrated in increasing ethanol solutions (in accordance with the standard procedures). Coverslips with cells were then inverted (with the cells facing downwards) and placed on top of BEEM® capsules filled with Spurr resin (Electron Microscopy Sciences, Hatfield, PA, USA). The polymerization of the resin was carried out at a temperature of 60 °C in a stove for a period of three days. At this point, the glass was removed by thermal contrast switch while the cell monolayer remained at the top of the polymerized block. A Leica ultramicrotome EM UC7 (Leica Microsystems, Wetzlar, Germany) was employed for sectioning. Ultra-thin sections (60–70 nm) were mounted on cooper grids and stained with 2% Uranyl-less solution for 10 min and with a lead-staining solution for 5 min. The sections were observed using a JEOL JEM-1010 transmission electron microscope (JEOL Ltd, Akishima, Tokyo, Japan) coupled with an Orius SC1000 CCD camera (model 832) (Gatan Inc., Pleasanton, CA, USA) at the Electron Microscopy Unit from the Scientific and Technological Centers of the University of Barcelona.

The circularity of mitochondria was analyzed using the follow statement: A circularity value of 1.0 indicates a perfect circle. As the value approaches zero, it indicates an increasingly elongated polygon. The ImageJ (Fiji) software incorporates the capacity to perform circularity calculations. Quantification was performed based on the analysis of 30–60 mitochondria analyzed from 17–20 cells, comprising both control and senescent cells, across three independent experiments.

To quantify and compare the number of lysosomes in control and senescent cells we used images captured always at the same magnification (×30.000), with each field representing an area of 25 μm^2^. The total number of lysosomes counted ranged from 49 in the control to 183 in the senescent cells.

### Immunofluorescence

Cells were gently washed once with cold PBS and then fixed for 20 min with 4% paraformaldehyde in PBS. Cells were then washed three times with PBS and permeabilized for 10 min with 0.1% saponin in PBS. Blocking was performed for 5 min with 0.02% saponin and 1% BSA in PBS. For primary antibody incubation, cells were incubated with anti-Tom20 (ab186734, Abcam) diluted in 0.02% saponin and 0.1% BSA solution for 1 h at room temperature. After primary antibody incubation, cells were washed three times with PBS. Secondary antibody incubation was carried out by incubating cells with anti-Mouse IgG (H + L), Superclonal™ Recombinant Secondary Antibody Alexa Fluor™ 488 (A28175, Thermo Fisher) and anti-Rabbit IgG (H + L) Cross-Adsorbed Secondary Antibody, Alexa Fluor 555 (A21428, Thermo Fisher) for 45 min in 0.02% saponin and 0.1% BSA solution at RT. For DAPI staining, cells were incubated with diluted DAPI stock solution (10374168, Thermo Fisher) to a final concentration of 300 nM in PBS for 5 min. Following staining, cells were washed three times with PBS, rinsed in water and mounted in Mowiol^®^ 4–88 (17951–500, Polysciences, Warrington, PA, USA).

### siRNA-mediated gene knockdown

For knockdown experiments, cells were transfected with HRK siRNA SMARTpool (77L-HUMAN-XX-0005, Horizon Discovery, Cambridge, UK) or non-targeting siRNA pool (77D-001810-10-7505, Horizon Discovery) using Lipofectamine® RNAiMax (13778-075, Thermo Fisher). Briefly, 10^5^ cells per condition were seeded in 12-well plates. Lipofectamine® RNAiMax and siRNAs were diluted in Opti-MEM® medium (31985062, Thermo Fisher) according to the manufacturer’s instructions, and the siRNA-lipid complex was added to the cells. The following day, cells were replenished with fresh media and maintained in culture for 72 h for further analysis.

### Bioinformatic analysis of SK-MEL-103 data

In the present study, GEO2R (www.ncbi.nlm.nih.gov/geo/geo2r/) was utilized to download the raw counts of the GSE246690 series to identify genes differentially expressed across experimental conditions of the SK-MEL-103 cell line (control and palbociclib) [34]. The data was analyzed using the edgeR (v.4.0.16) package from Bioconductor in the R environment (v.4.3.3) [40]. The sva (v. 3.50.0) package was used to remove batch effects and other unwanted variation [41]. Subsequently, differential expression analysis was conducted using the quasi-likelihood negative binomial generalized log-linear model (GLM) functions provided by the edgeR package. For visualization purposes Log2CPM expression values were converted to Z-scores and statistical significance was represented as Fold Discovery Rate (FDR) < 0.05.

### Patients and samples for molecular validation

Fourteen healthy subjects, comprising 10 female and 4 male, mean age 52.6 years (range 41–66) were recruited in Hospital Clínic de Barcelona (Table 1). Two distinct areas on the forearm were identified for biopsy: one area of photodamaged skin (L01) and one area of less sun-damaged (naturally protected) skin on the inner forearm (L02). These samples were obtained as part of another project led by Dr. Susana Puig following all ethical rules.

Subsequently, two 3-mm punch biopsies were taken on L01 and L02 areas. Each biopsy was included in RNAlater (RNAprotect Tissue Reagent, Qiagen, The Netherlands) and frozen to extract RNA and perform sequencing.

### RNA extraction and RNA-sequencing analysis

Total RNA was isolated using the RNeasy Mini kit (74104, Qiagen) following the manufacturer’s instructions. One microgram of RNA was reverse transcribed using the High-Capacity cDNA Reverse Transcription Kit (4368814, Applied Biosystems, Thermo) with random hexamer primers. A 1:10 dilution of the resulting cDNA was used for quantitative real-time PCR (qPCR) on the Lightcycler 96 instrument (05815916001, Roche) with the Brilliant SYBR® Green QPCR Master Mix (600548, Stratagene, Sigma-Aldrich).

Data was normalized to the expression of the housekeeping gene RPL13, and the difference between treated and untreated cells was calculated as ΔΔCt. For visualization, fold changes were determined using the formula 2-ΔΔCt. The qPCR primers used for mRNA level analysis included HRK Rv-GCTTTCTCCAAGGACACAGGG, Fw-ACCTACTGGCCTTGGCTGTG, and RPL13 Rv- CTCGGGAGGGGTTGGTATTCATC, Fw- ATGGCGGAGGGGCAGGTTCT.

### SK-MEL-103 tumor xenografts

Tumor xenografts from SK-MEL-103 cells were generated as previously described [35]. Briefly, a suspension of 2×10^5^ SK-MEL-103 cells in PBS and matrigel was subcutaneously injected in the flank of athymic nude mice. Once tumors were visible, a group of mice received 100 mg/kg of palbociclib by oral gavage in 50 mM sodium lactate every day for 9 days, and the other group received vehicle. Tumor growth was monitored by caliper measurements and was then extracted and frozen for western blot analysis.

### Statistical analysis

Data were analyzed using GraphPad Prism v.9.3.0 software and was represented as mean ± SEM of independent biological replicates. The statistical significance of the results was analyzed using Student’s *t*-tail test. *p < 0.05 and **p  <  0.01 were considered significant.

Specifically, for RNA-Seq data analysis of patient samples, the R package LIMMA was used for data normalization, specifically employing the Variance Modeling at the Observational Level (VOOM). After normalization of expression data, the genes of interest for validation (CDKN2A, CDKN1A, and HRK) were selected. Global results of the RNA-sequencing analysis are under revision for their publication. Comparisons between the expression between L01 and L02 samples from the same patient were conducted using a paired Student’s *t*-test. Assumptions of normality and homogeneity of variances were checked and appropriately addressed. All analyses were performed using R version 3.3.0.

## Supplementary information

Supplementary information

Supplementary original blots

Supplementary figure legend

## Data Availability

All data are available from the corresponding authors upon reasonable request.

## References

[1] Campisi J. Cellular senescence as a tumor-suppressor mechanism. Trends Cell Biol. 2001;11:S27–31. 11684439 10.1016/s0962-8924(01)02151-1

[2] Schmitt CA, Wang B, Demaria M. Senescence and cancer - role and therapeutic opportunities. Nat Rev Clin Oncol. 2022;19:619–36. 36045302 10.1038/s41571-022-00668-4 PMC9428886

[3] Demaria M, O’Leary MN, Chang J, Shao L, Liu S, Alimirah F, et al. Cellular senescence promotes adverse effects of chemotherapy and cancer relapse. Cancer Discov. 2017;7:165–76. 27979832 10.1158/2159-8290.CD-16-0241 PMC5296251

[4] Zhang L, Pitcher LE, Yousefzadeh MJ, Niedernhofer LJ, Robbins PD, Zhu Y. Cellular senescence: a key therapeutic target in aging and diseases. J Clin Invest. 2022;132:e158450. 10.1172/JCI158450 PMC9337830 35912854

[5] Munoz-Espin D, Serrano M. Cellular senescence: from physiology to pathology. Nat Rev Mol Cell Biol. 2014;15:482–96. 24954210 10.1038/nrm3823

[6] Ewald JA, Desotelle JA, Wilding G, Jarrard DF. Therapy-induced senescence in cancer. J Natl Cancer Inst. 2010;102:1536–46. 20858887 10.1093/jnci/djq364 PMC2957429

[7] Maggiorani D, Le O, Lisi V, Landais S, Moquin-Beaudry G, Lavallee VP, et al. Senescence drives immunotherapy resistance by inducing an immunosuppressive tumor microenvironment. Nat Commun. 2024;15:2435. 38499573 10.1038/s41467-024-46769-9 PMC10948808

[8] Watt AC, Cejas P, DeCristo MJ, Metzger-Filho O, Lam EYN, Qiu X, et al. CDK4/6 inhibition reprograms the breast cancer enhancer landscape by stimulating AP-1 transcriptional activity. Nat Cancer. 2021;2:34–48. 33997789 10.1038/s43018-020-00135-y PMC8115221

[9] Chaib S, Tchkonia T, Kirkland JL. Cellular senescence and senolytics: the path to the clinic. Nat Med. 2022;28:1556–68. 35953721 10.1038/s41591-022-01923-y PMC9599677

[10] Gasek NS, Kuchel GA, Kirkland JL, Xu M. Strategies for targeting senescent cells in human disease. Nat Aging. 2021;1:870–9. 34841261 10.1038/s43587-021-00121-8 PMC8612694

[11] Zhu Y, Tchkonia T, Fuhrmann-Stroissnigg H, Dai HM, Ling YY, Stout MB, et al. Identification of a novel senolytic agent, navitoclax, targeting the Bcl-2 family of anti-apoptotic factors. Aging Cell. 2016;15:428–35. 26711051 10.1111/acel.12445 PMC4854923

[12] Zhu Y, Tchkonia T, Pirtskhalava T, Gower AC, Ding H, Giorgadze N, et al. The Achilles’ heel of senescent cells: from transcriptome to senolytic drugs. Aging Cell. 2015;14:644–58. 25754370 10.1111/acel.12344 PMC4531078

[13] Childs BG, Baker DJ, Kirkland JL, Campisi J, van Deursen JM. Senescence and apoptosis: dueling or complementary cell fates? EMBO Rep. 2014;15:1139–53. 25312810 10.15252/embr.201439245 PMC4253488

[14] Montero J, Haq R. Adapted to survive: targeting cancer cells with BH3 mimetics. Cancer Discov. 2022;12:1217–32. 35491624 10.1158/2159-8290.CD-21-1334 PMC9306285

[15] Debrincat MA, Pleines I, Lebois M, Lane RM, Holmes ML, Corbin J, et al. BCL-2 is dispensable for thrombopoiesis and platelet survival. Cell Death Dis. 2015;6:e1721. 25880088 10.1038/cddis.2015.97 PMC4650559

[16] Khan S, Zhang X, Lv D, Zhang Q, He Y, Zhang P, et al. A selective BCL-X(L) PROTAC degrader achieves safe and potent antitumor activity. Nat Med. 2019;25:1938–47. 31792461 10.1038/s41591-019-0668-z PMC6898785

[17] Singh R, Letai A, Sarosiek K. Regulation of apoptosis in health and disease: the balancing act of BCL-2 family proteins. Nat Rev Mol Cell Biol. 2019;20:175–93. 30655609 10.1038/s41580-018-0089-8 PMC7325303

[18] Kohli J, Ge C, Fitsiou E, Doepner M, Brandenburg SM, Faller WJ, et al. Targeting anti-apoptotic pathways eliminates senescent melanocytes and leads to nevi regression. Nat Commun. 2022;13:7923. 36564381 10.1038/s41467-022-35657-9 PMC9789033

[19] Certo M, Del Gaizo Moore V, Nishino M, Wei G, Korsmeyer S, Armstrong SA, et al. Mitochondria primed by death signals determine cellular addiction to antiapoptotic BCL-2 family members. Cancer Cell. 2006;9:351–65. 16697956 10.1016/j.ccr.2006.03.027

[20] Ryan J, Letai A. BH3 profiling in whole cells by fluorimeter or FACS. Methods. 2013;61:156–64. 23607990 10.1016/j.ymeth.2013.04.006 PMC3686919

[21] Ni Chonghaile T, Sarosiek KA, Vo TT, Ryan JA, Tammareddi A, Moore Vdel G, et al. Pretreatment mitochondrial priming correlates with clinical response to cytotoxic chemotherapy. Science. 2011;334:1129–33. 22033517 10.1126/science.1206727 PMC3280949

[22] Ryan J, Montero J, Rocco J, Letai A. iBH3: simple, fixable BH3 profiling to determine apoptotic priming in primary tissue by flow cytometry. Biol Chem. 2016;397:671–8. 26910743 10.1515/hsz-2016-0107

[23] Montero J, Sarosiek KA, DeAngelo JD, Maertens O, Ryan J, Ercan D, et al. Drug-induced death signaling strategy rapidly predicts cancer response to chemotherapy. Cell. 2015;160:977–89. 25723171 10.1016/j.cell.2015.01.042 PMC4391197

[24] Montero J, Gstalder C, Kim DJ, Sadowicz D, Miles W, Manos M, et al. Destabilization of NOXA mRNA as a common resistance mechanism to targeted therapies. Nat Commun. 2019;10:5157. 31727958 10.1038/s41467-019-12477-y PMC6856172

[25] Alcon C, Manzano-Munoz A, Prada E, Mora J, Soriano A, Guillen G, et al. Sequential combinations of chemotherapeutic agents with BH3 mimetics to treat rhabdomyosarcoma and avoid resistance. Cell Death Dis. 2020;11:634. 32801295 10.1038/s41419-020-02887-y PMC7429859

[26] Montero J, Letai A. Why do BCL-2 inhibitors work and where should we use them in the clinic? Cell Death Differ. 2018;25:56–64. 29077093 10.1038/cdd.2017.183 PMC5729538

[27] Selt F, Sigaud R, Valinciute G, Sievers P, Zaman J, Alcon C, et al. BH3 mimetics targeting BCL-XL impact the senescent compartment of pilocytic astrocytoma. Neuro Oncol. 2023;25:735–47. 35977048 10.1093/neuonc/noac199 PMC10076946

[28] Verhaegen M, Bauer JA, Martin de la Vega C, Wang G, Wolter KG, Brenner JC, et al. A novel BH3 mimetic reveals a mitogen-activated protein kinase-dependent mechanism of melanoma cell death controlled by p53 and reactive oxygen species. Cancer Res. 2006;66:11348–59. 17145881 10.1158/0008-5472.CAN-06-1748

[29] Vilella R, Benitez D, Mila J, Vilalta A, Rull R, Cuellar F, et al. Treatment of patients with progressive unresectable metastatic melanoma with a heterologous polyvalent melanoma whole cell vaccine. Int J Cancer. 2003;106:626–31. 12845663 10.1002/ijc.11242

[30] Kovatcheva M, Liu DD, Dickson MA, Klein ME, O’Connor R, Wilder FO, et al. MDM2 turnover and expression of ATRX determine the choice between quiescence and senescence in response to CDK4 inhibition. Oncotarget. 2015;6:8226–43. 25803170 10.18632/oncotarget.3364 PMC4480747

[31] Hernandez-Segura A, Rubingh R, Demaria M. Identification of stable senescence-associated reference genes. Aging Cell. 2019;18:e12911. 30710410 10.1111/acel.12911 PMC6413663

[32] Freedberg DE, Rigas SH, Russak J, Gai W, Kaplow M, Osman I, et al. Frequent p16-independent inactivation of p14ARF in human melanoma. J Natl Cancer Inst. 2008;100:784–95. 18505964 10.1093/jnci/djn157 PMC4410798

[33] Sebastian D, Palacin M, Zorzano A. Mitochondrial dynamics: coupling mitochondrial fitness with healthy aging. Trends Mol Med. 2017;23:201–15. 28188102 10.1016/j.molmed.2017.01.003

[34] Rovira M, Sereda R, Pladevall-Morera D, Ramponi V, Marin I, Maus M, et al. The lysosomal proteome of senescent cells contributes to the senescence secretome. Aging Cell. 2022;21:e13707. 36087066 10.1111/acel.13707 PMC9577959

[35] Lopez-Polo V, Maus M, Zacharioudakis E, Lafarga M, Attolini CS, Marques FDM, et al. Release of mitochondrial dsRNA into the cytosol is a key driver of the inflammatory phenotype of senescent cells. Nat Commun. 2024;15:7378. 39191740 10.1038/s41467-024-51363-0 PMC11349883

[36] Chen L, Willis SN, Wei A, Smith BJ, Fletcher JI, Hinds MG, et al. Differential targeting of prosurvival Bcl-2 proteins by their BH3-only ligands allows complementary apoptotic function. Mol Cell. 2005;17:393–403. 15694340 10.1016/j.molcel.2004.12.030

[37] Saleh T, Tyutyunyk-Massey L, Gewirtz DA. Tumor cell escape from therapy-induced senescence as a model of disease recurrence after dormancy. Cancer Res. 2019;79:1044–6. 30803994 10.1158/0008-5472.CAN-18-3437

[38] Campisi J. Aging, cellular senescence, and cancer. Annu Rev Physiol. 2013;75:685–705. 23140366 10.1146/annurev-physiol-030212-183653 PMC4166529

[39] Jochems F, Thijssen B, De Conti G, Jansen R, Pogacar Z, Groot K, et al. The Cancer SENESCopedia: a delineation of cancer cell senescence. Cell Rep. 2021;36:109441. 34320349 10.1016/j.celrep.2021.109441 PMC8333195

[40] Robinson MD, McCarthy DJ, Smyth GK. edgeR: a bioconductor package for differential expression analysis of digital gene expression data. Bioinformatics. 2010;26:139–40. 19910308 10.1093/bioinformatics/btp616 PMC2796818

[41] Robinson MD, Oshlack A. A scaling normalization method for differential expression analysis of RNA-seq data. Genome Biol. 2010;11:R25. 20196867 10.1186/gb-2010-11-3-r25 PMC2864565

