# Supplementary material for: HRK downregulation and augmented BCL-xL binding to BAK confer apoptotic protection to therapy-induced senescent melanoma cells
Source: Cell Death Differ. 2024 Dec 3;32(4):646–56. doi: 10.1038/s41418-024-01417-z (PMC11982230; doi:10.1038/s41418-024-01417-z)

**Supplementary information**

**Supplementary Figure 1**


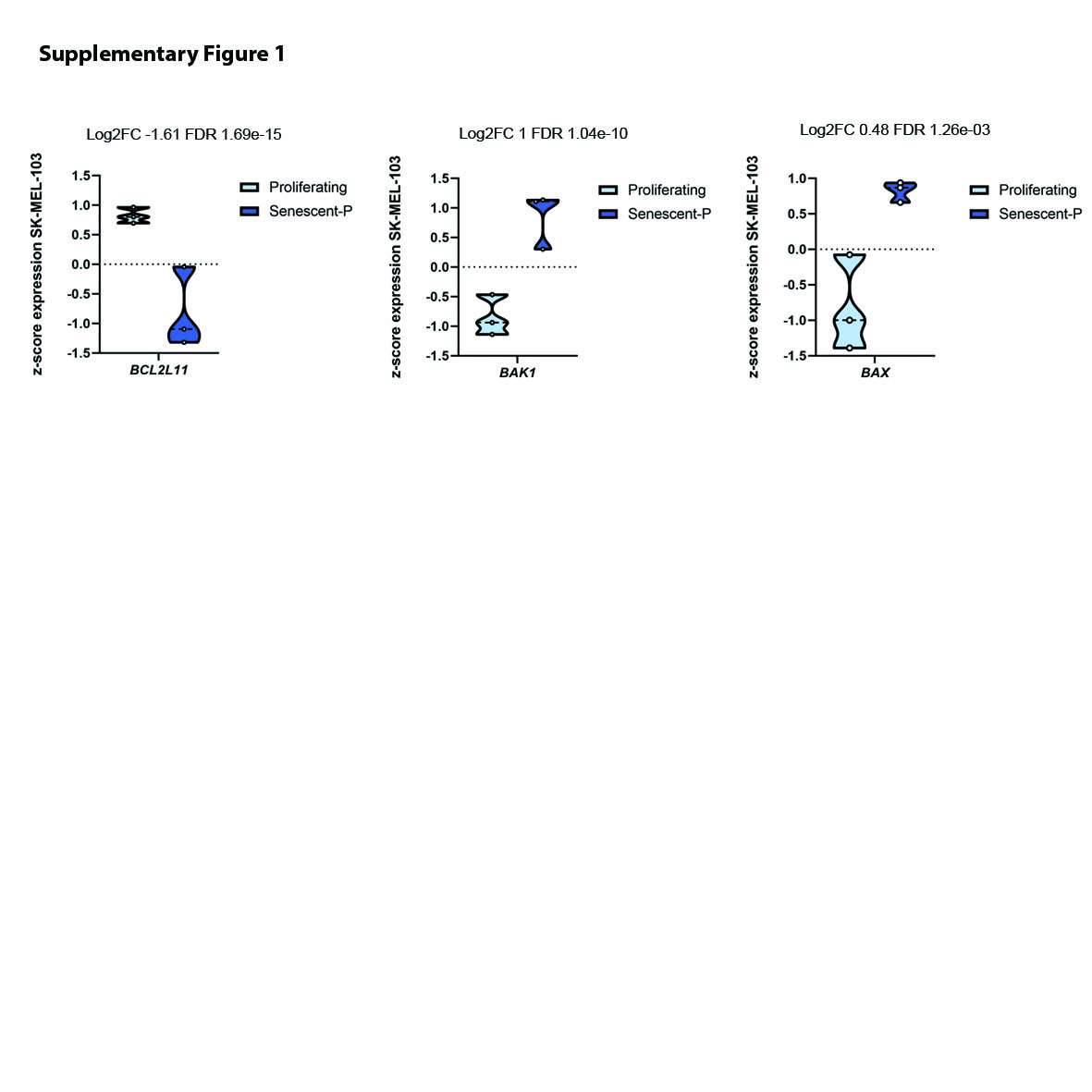


**Supplementary Figure 2**
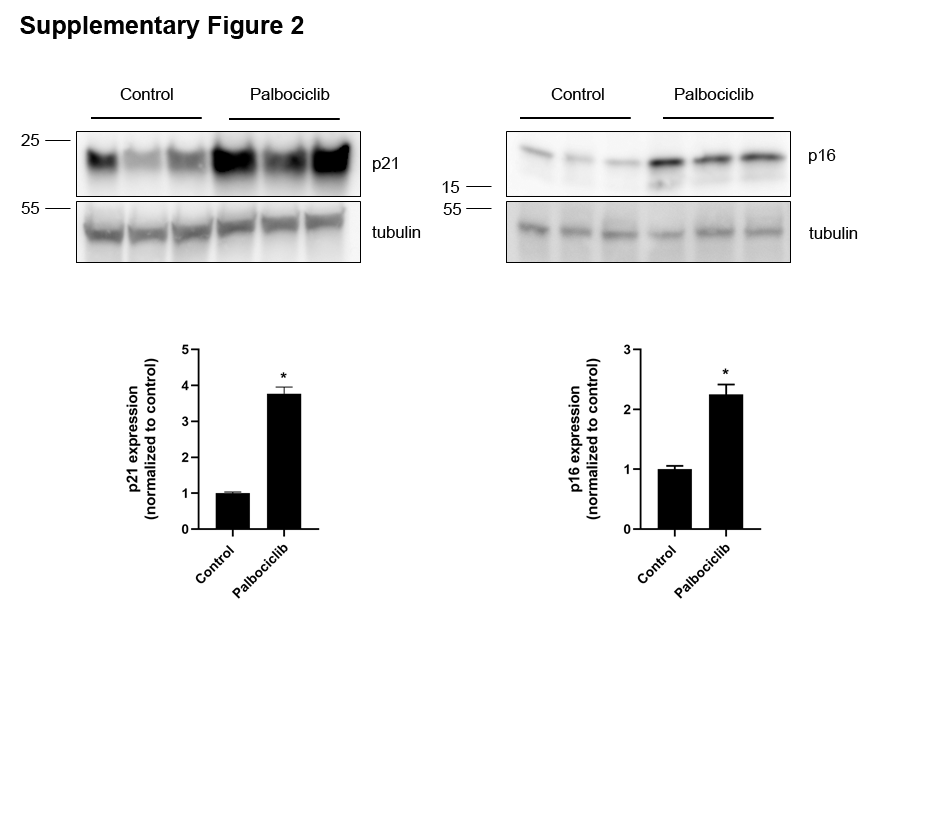


**Supplementary Figure 3**


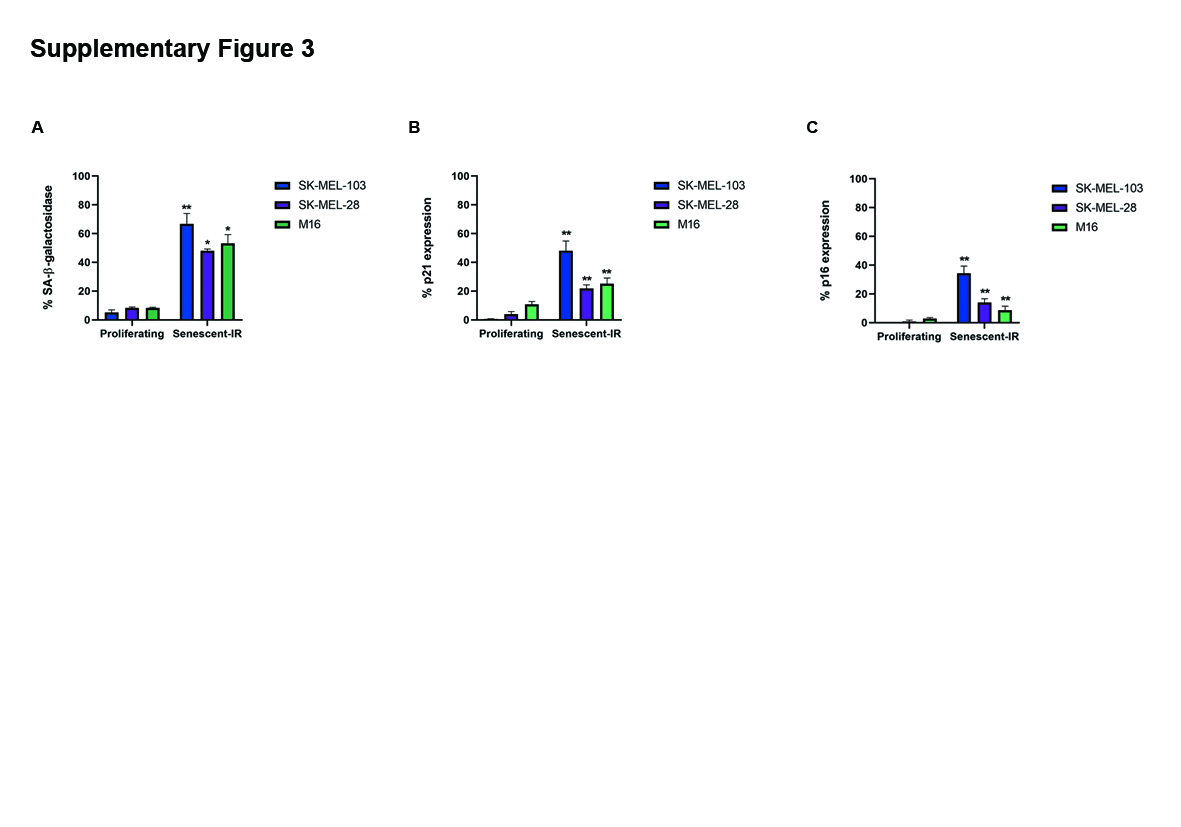


**Supplementary Figure 4**


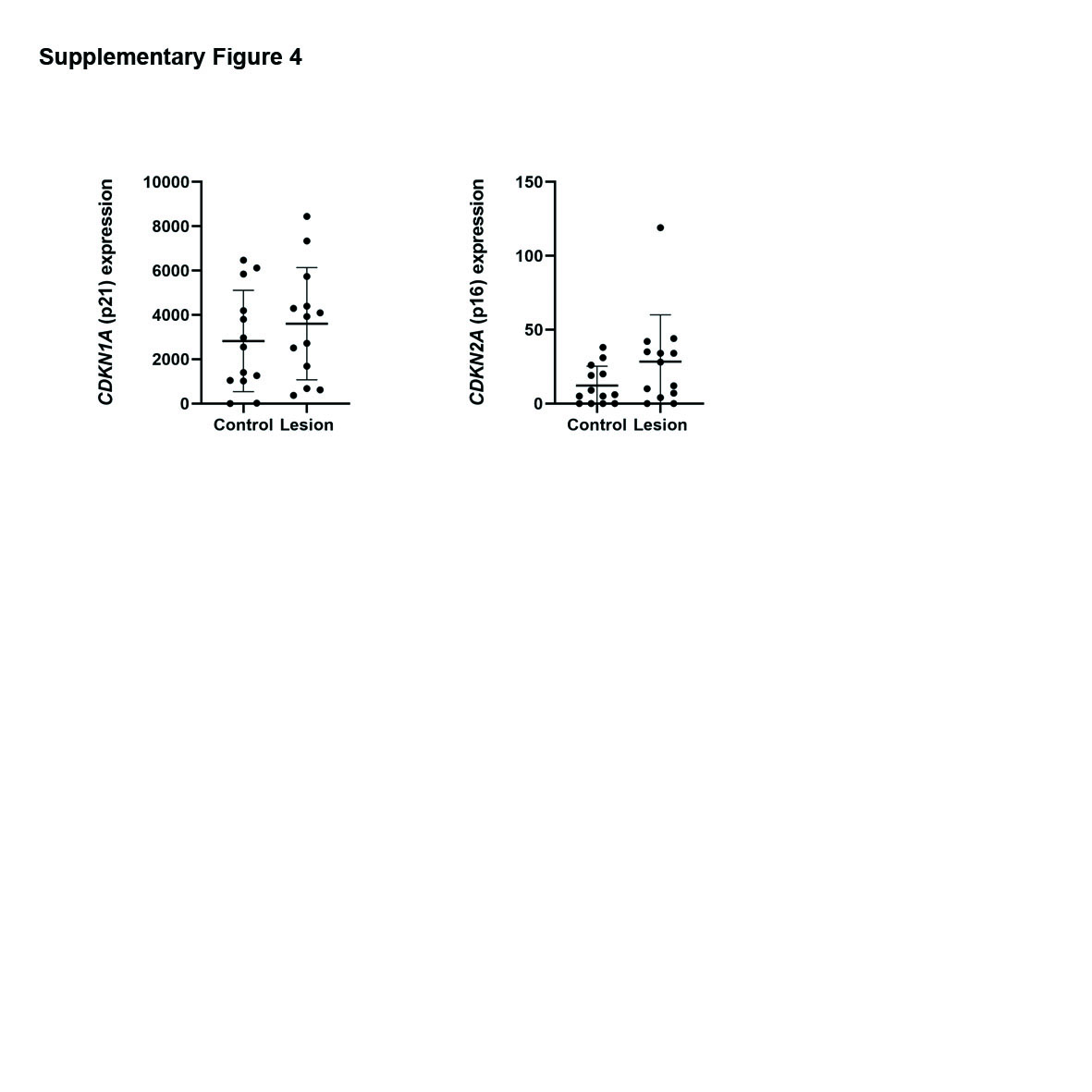


**Supplementary Figure 5**


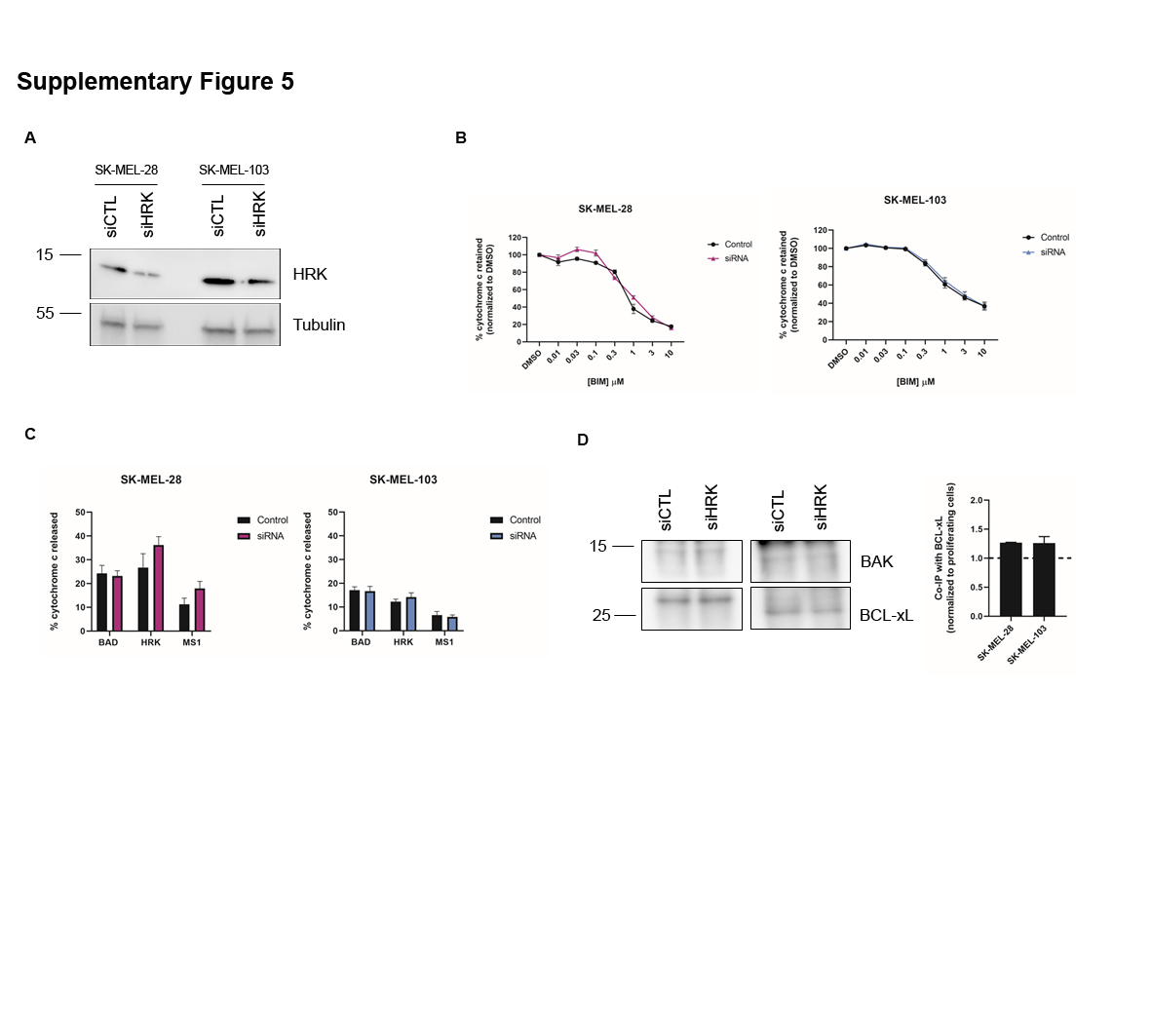

Supplement: Supplementary file 1 — Supplementary information [file 41418_2024_1417_MOESM1_ESM.docx]
