# Supplementary material for: HRK downregulation and augmented BCL-xL binding to BAK confer apoptotic protection to therapy-induced senescent melanoma cells
Source: Cell Death Differ. 2024 Dec 3;32(4):646–56. doi: 10.1038/s41418-024-01417-z (PMC11982230; doi:10.1038/s41418-024-01417-z)
Supplement: Supplementary file 2 — Supplementary original blots [file 41418_2024_1417_MOESM2_ESM.pptx]

## Slide 1
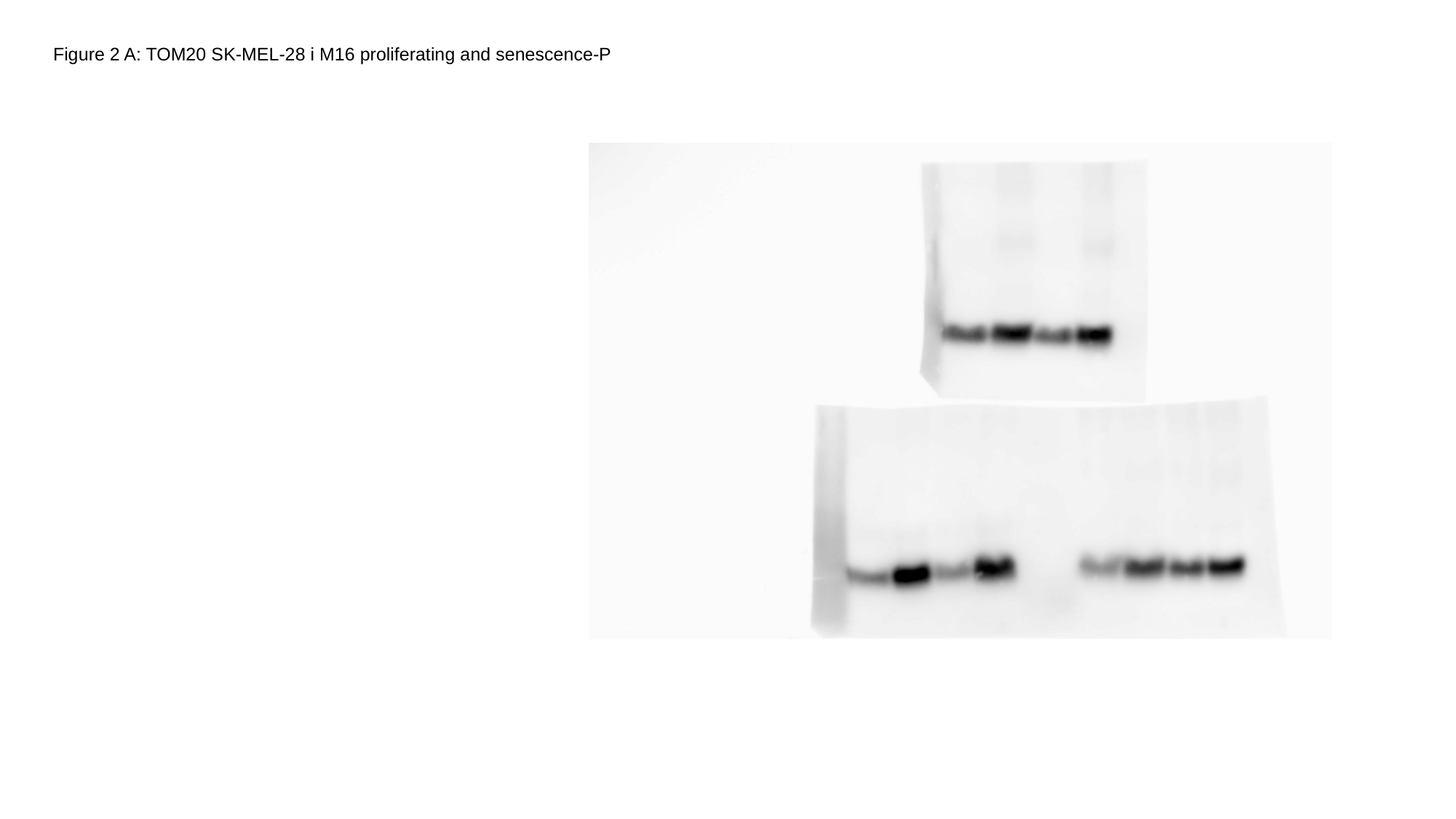

Figure 2 A: TOM20 SK-MEL-28 i M16 proliferating and senescence-P

## Slide 2
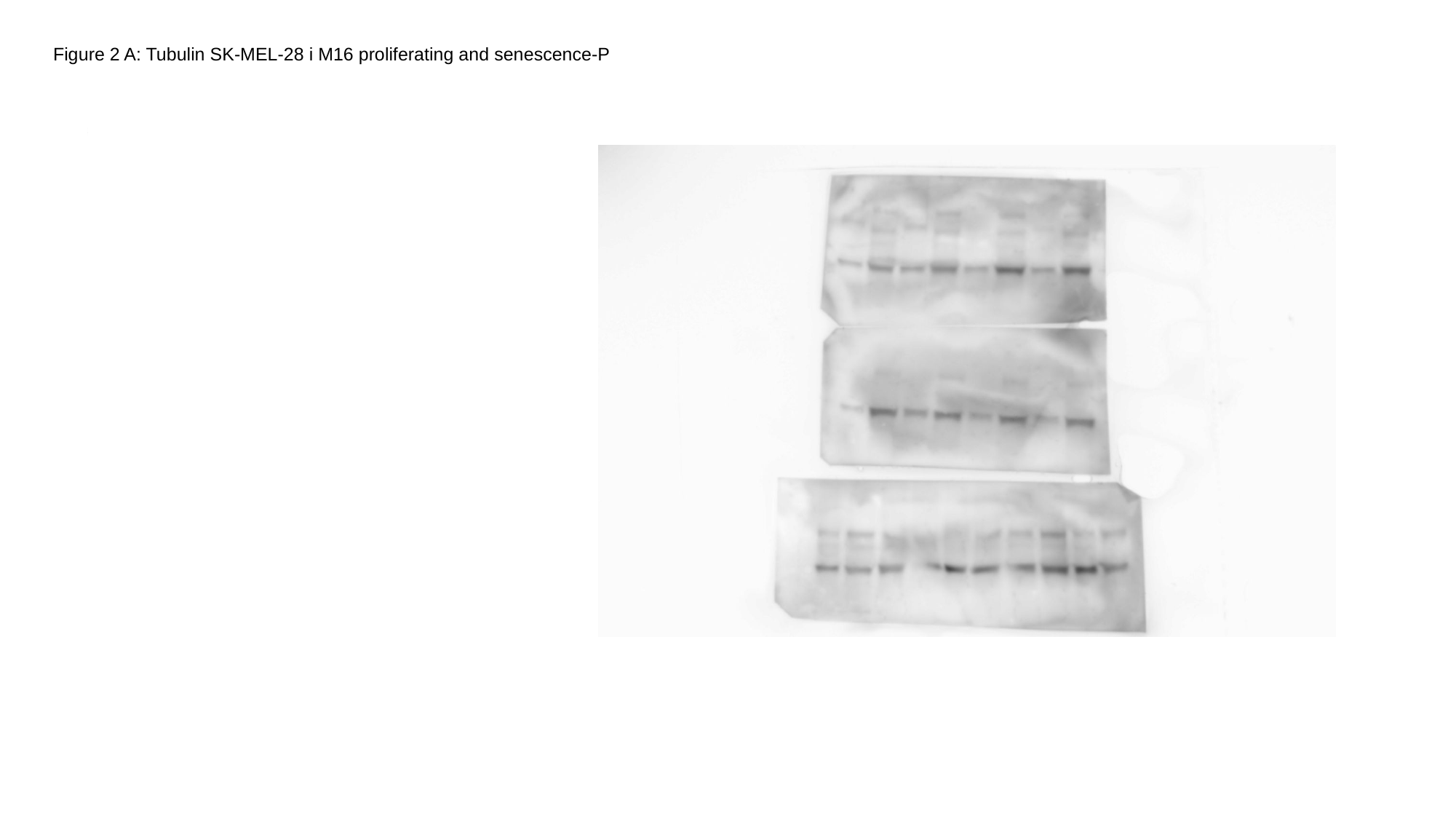

Figure 2 A: Tubulin SK-MEL-28 i M16 proliferating and senescence-P

## Slide 3
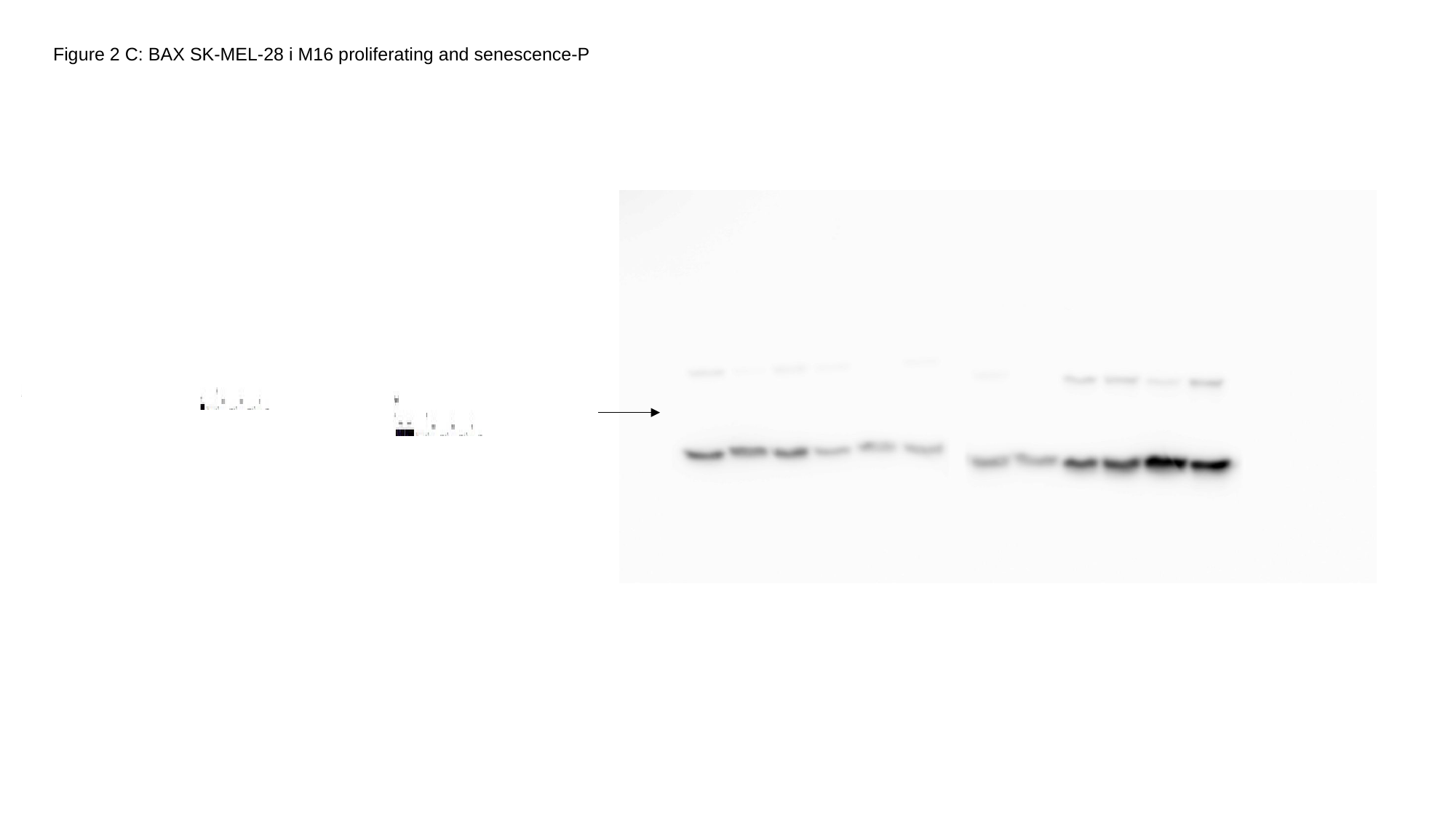

Figure 2 C: BAX SK-MEL-28 i M16 proliferating and senescence-P

## Slide 4
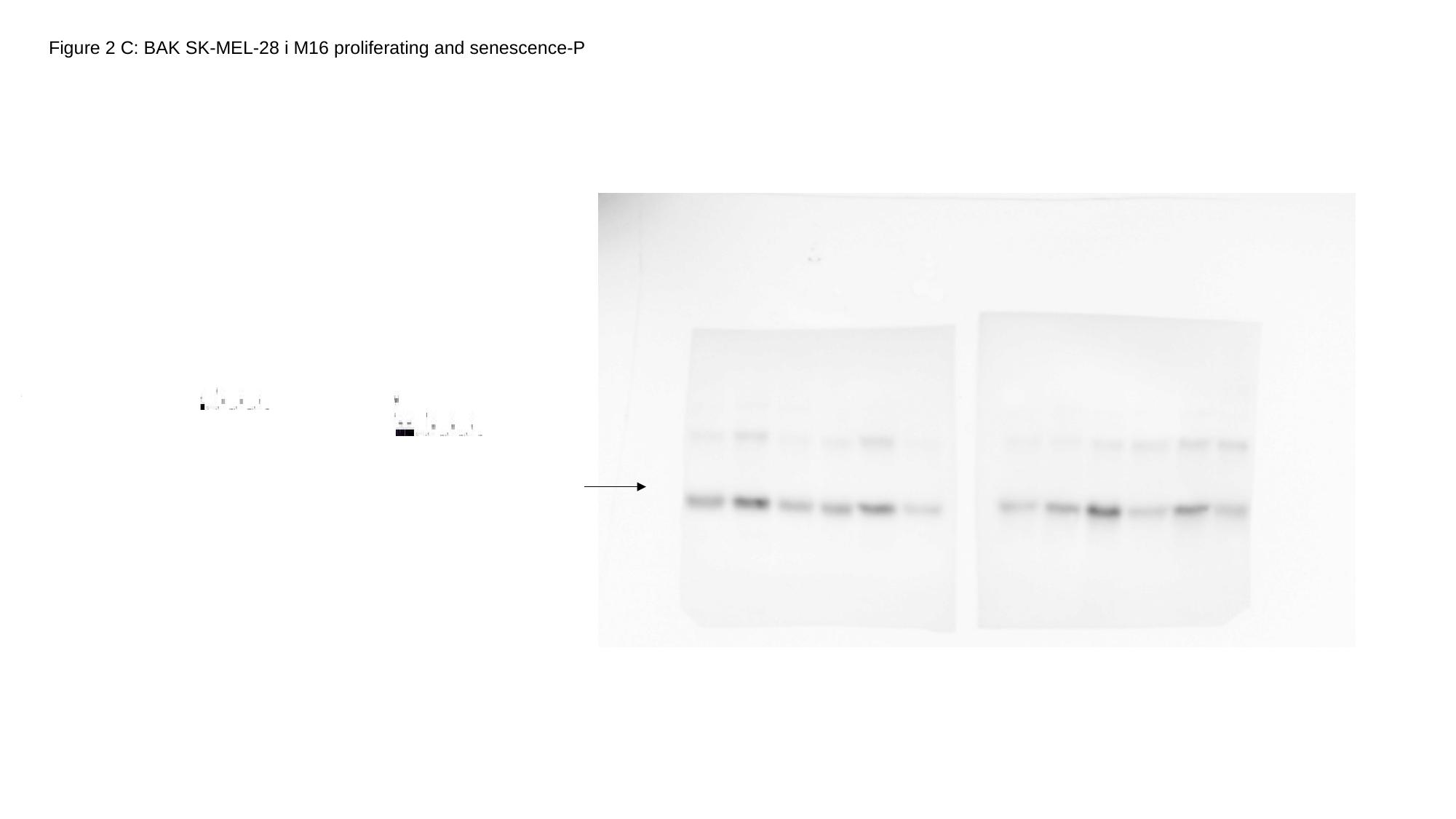

Figure 2 C: BAK SK-MEL-28 i M16 proliferating and senescence-P

## Slide 5
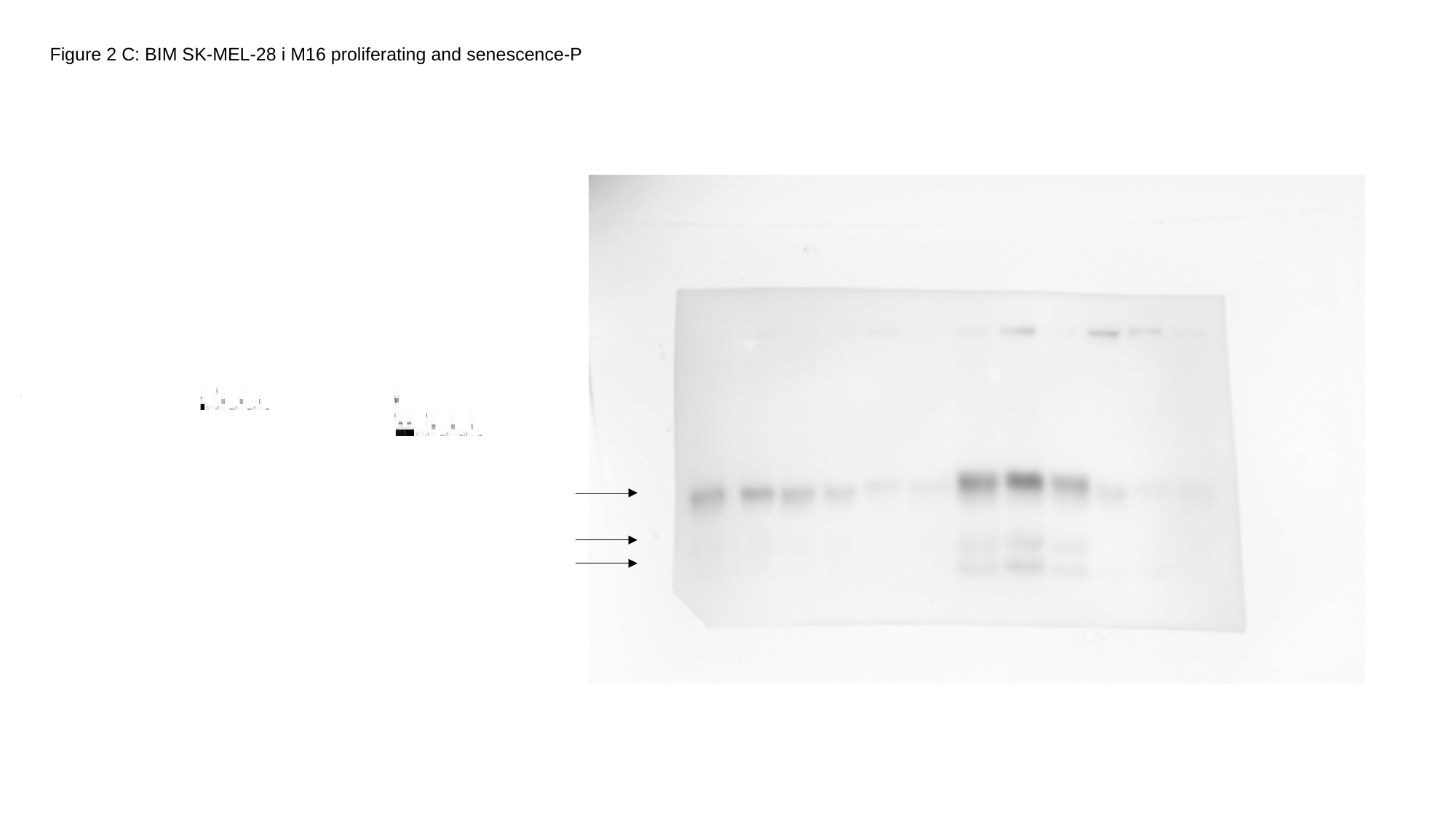

Figure 2 C: BIM SK-MEL-28 i M16 proliferating and senescence-P

## Slide 6
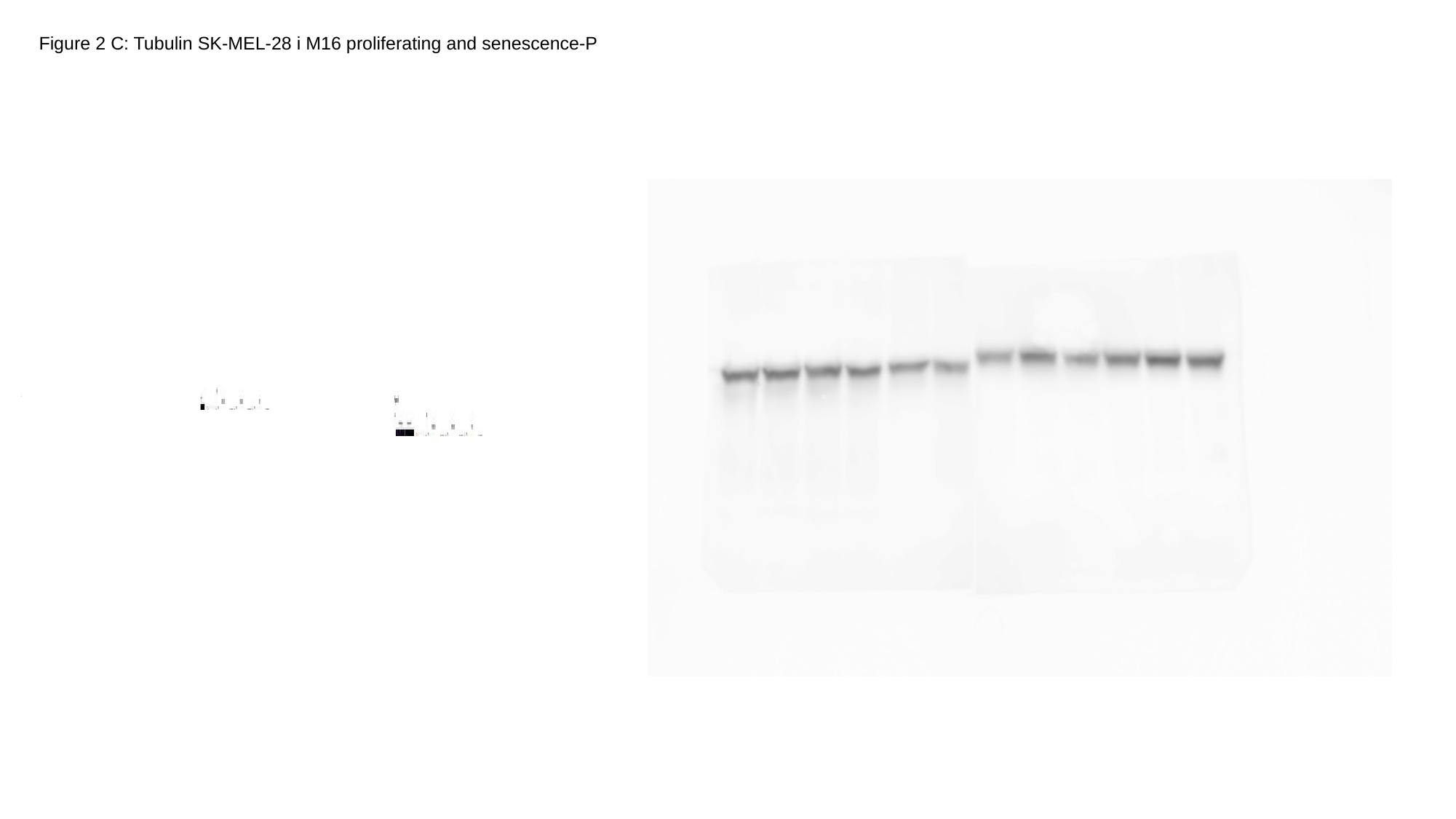

Figure 2 C: Tubulin SK-MEL-28 i M16 proliferating and senescence-P

## Slide 7
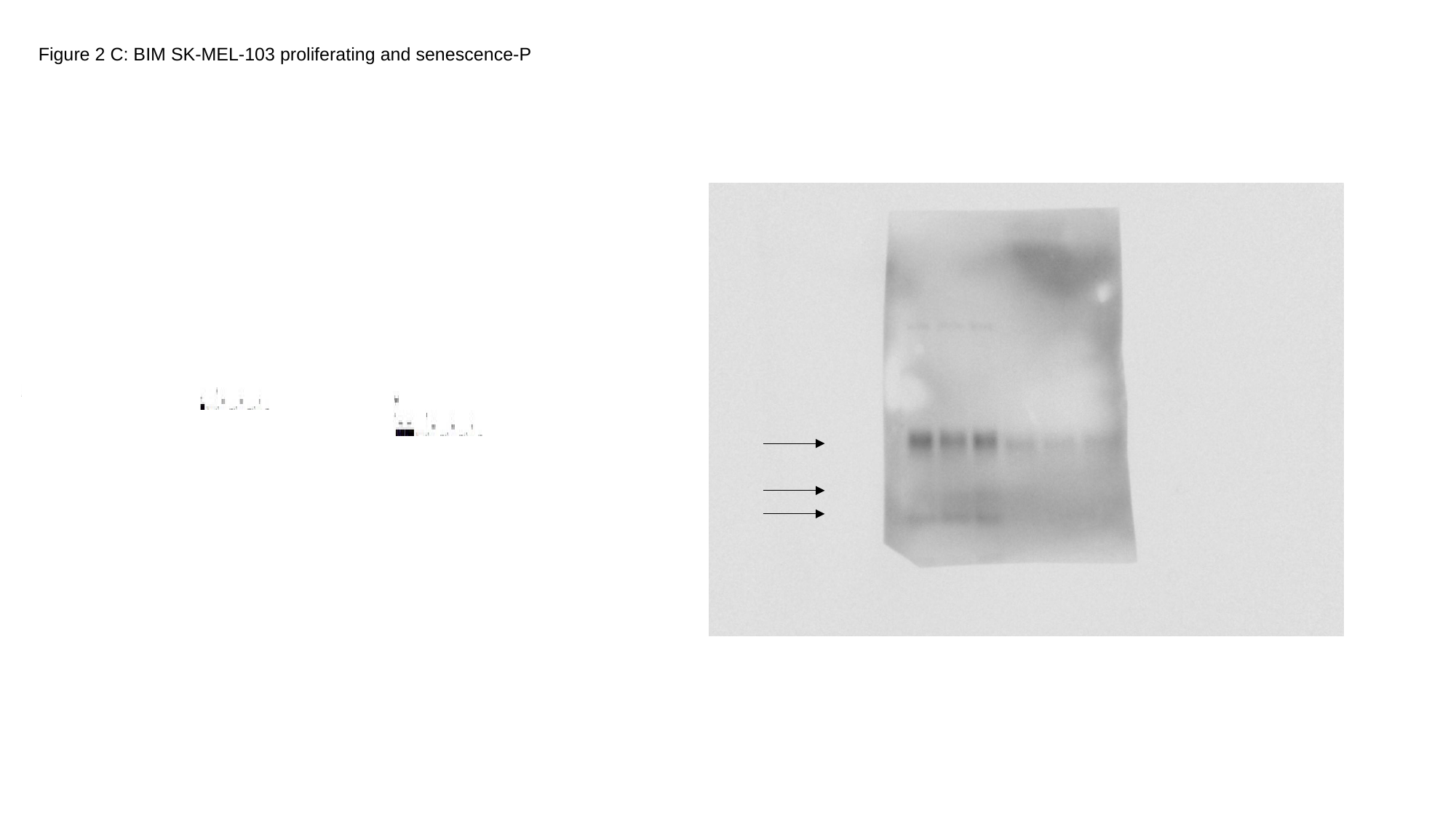

Figure 2 C: BIM SK-MEL-103 proliferating and senescence-P

## Slide 8
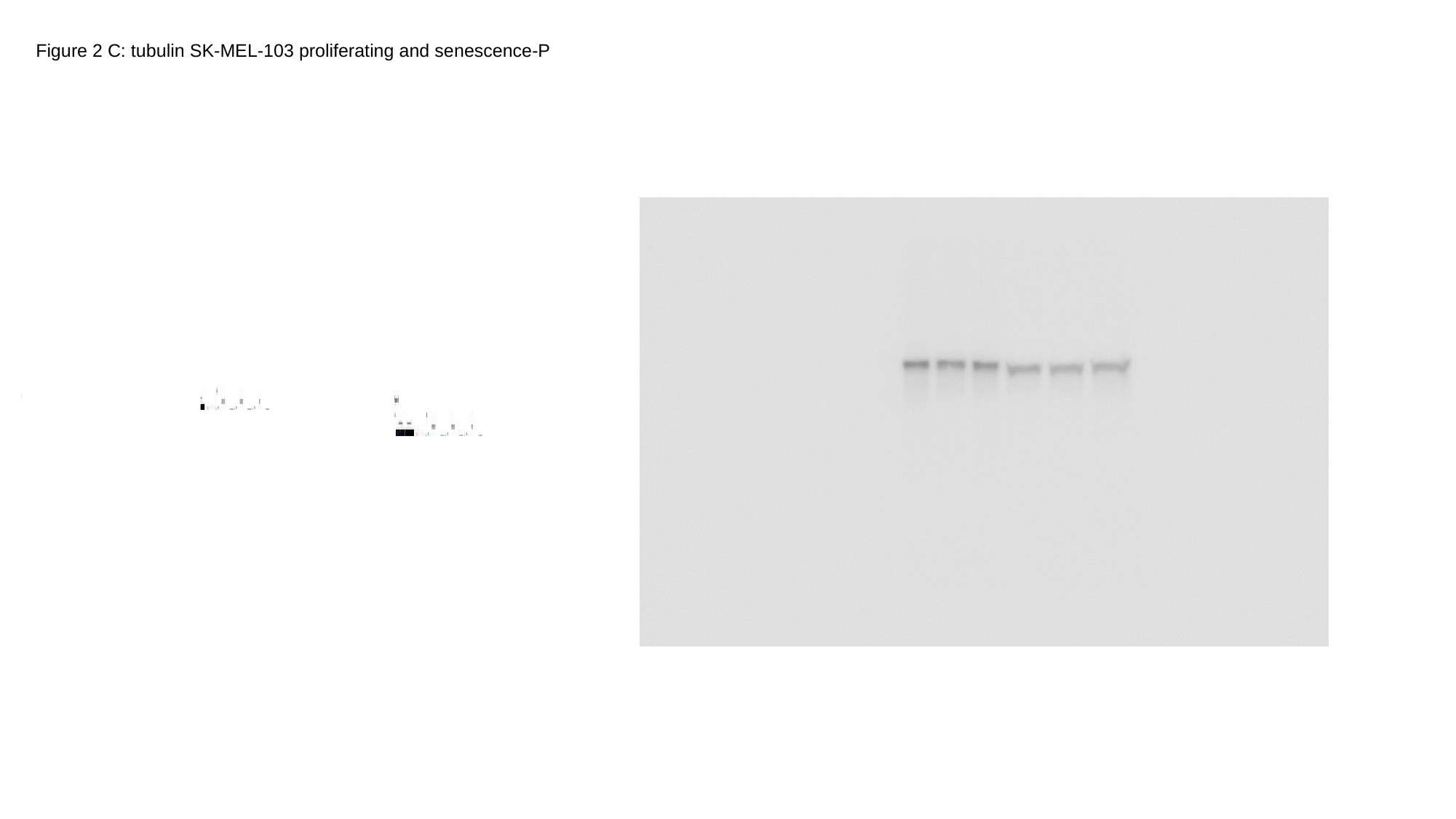

Figure 2 C: tubulin SK-MEL-103 proliferating and senescence-P

## Slide 9
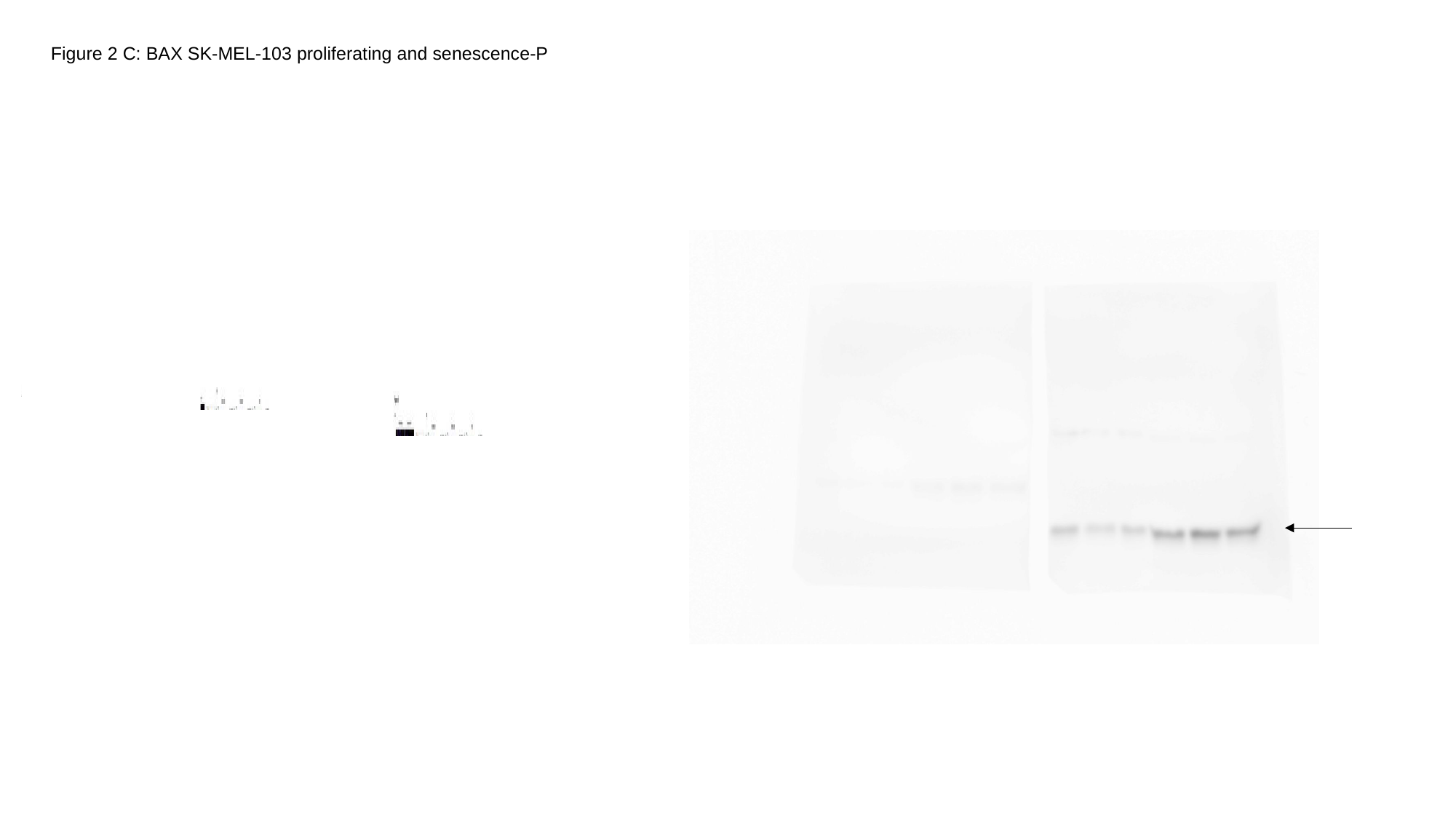

Figure 2 C: BAX SK-MEL-103 proliferating and senescence-P

## Slide 10
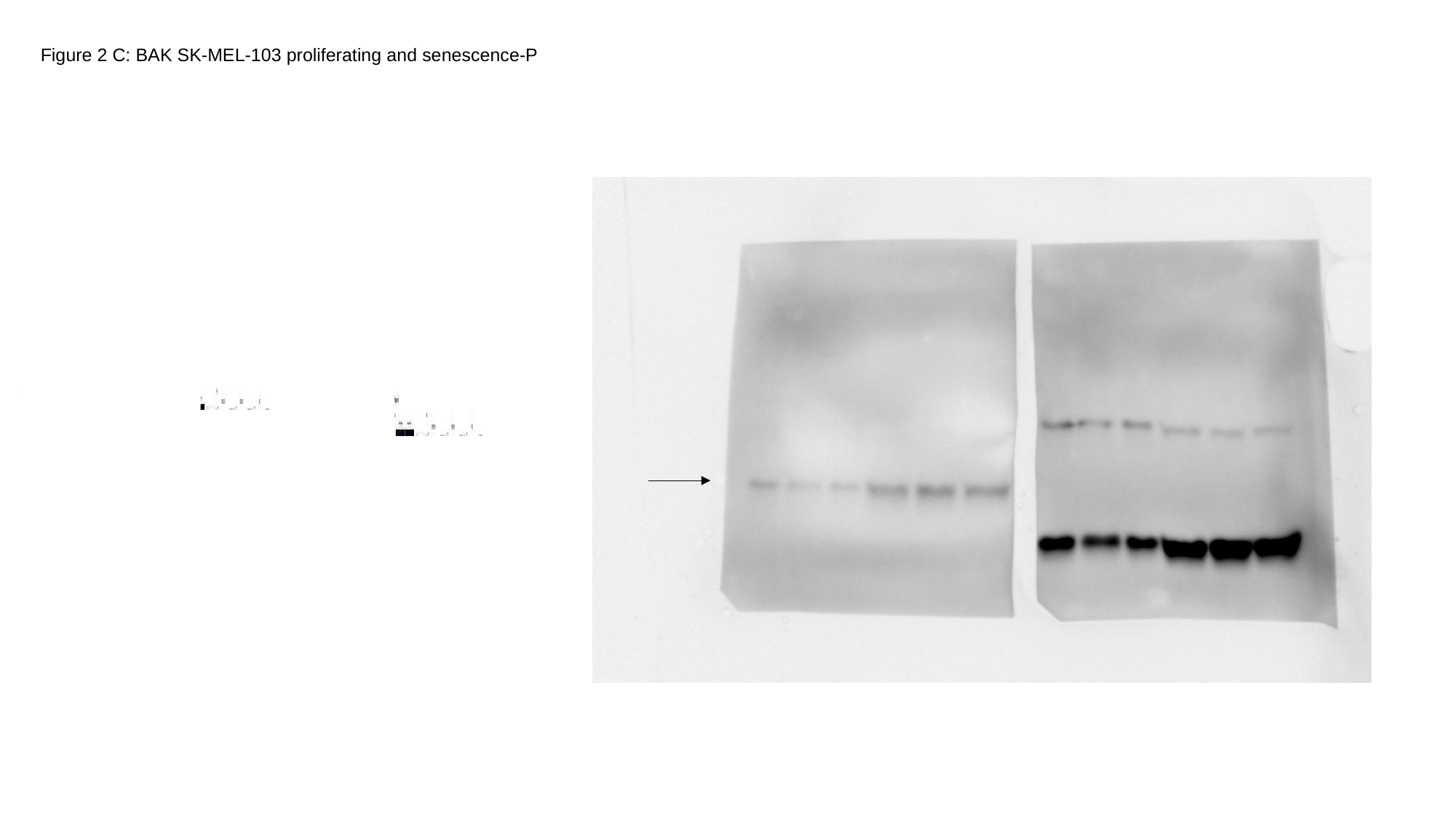

Figure 2 C: BAK SK-MEL-103 proliferating and senescence-P

## Slide 11
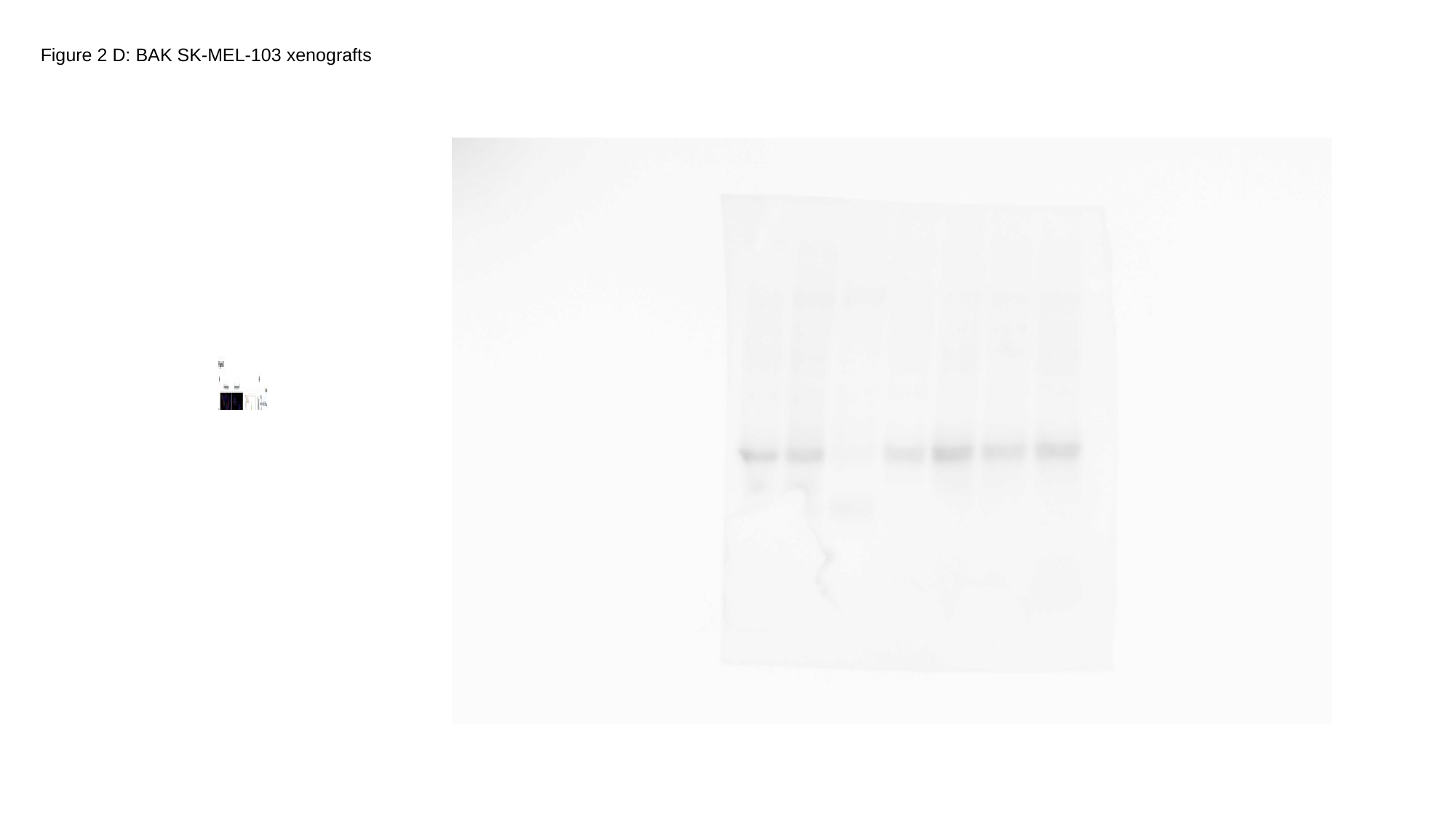

Figure 2 D: BAK SK-MEL-103 xenografts

## Slide 12
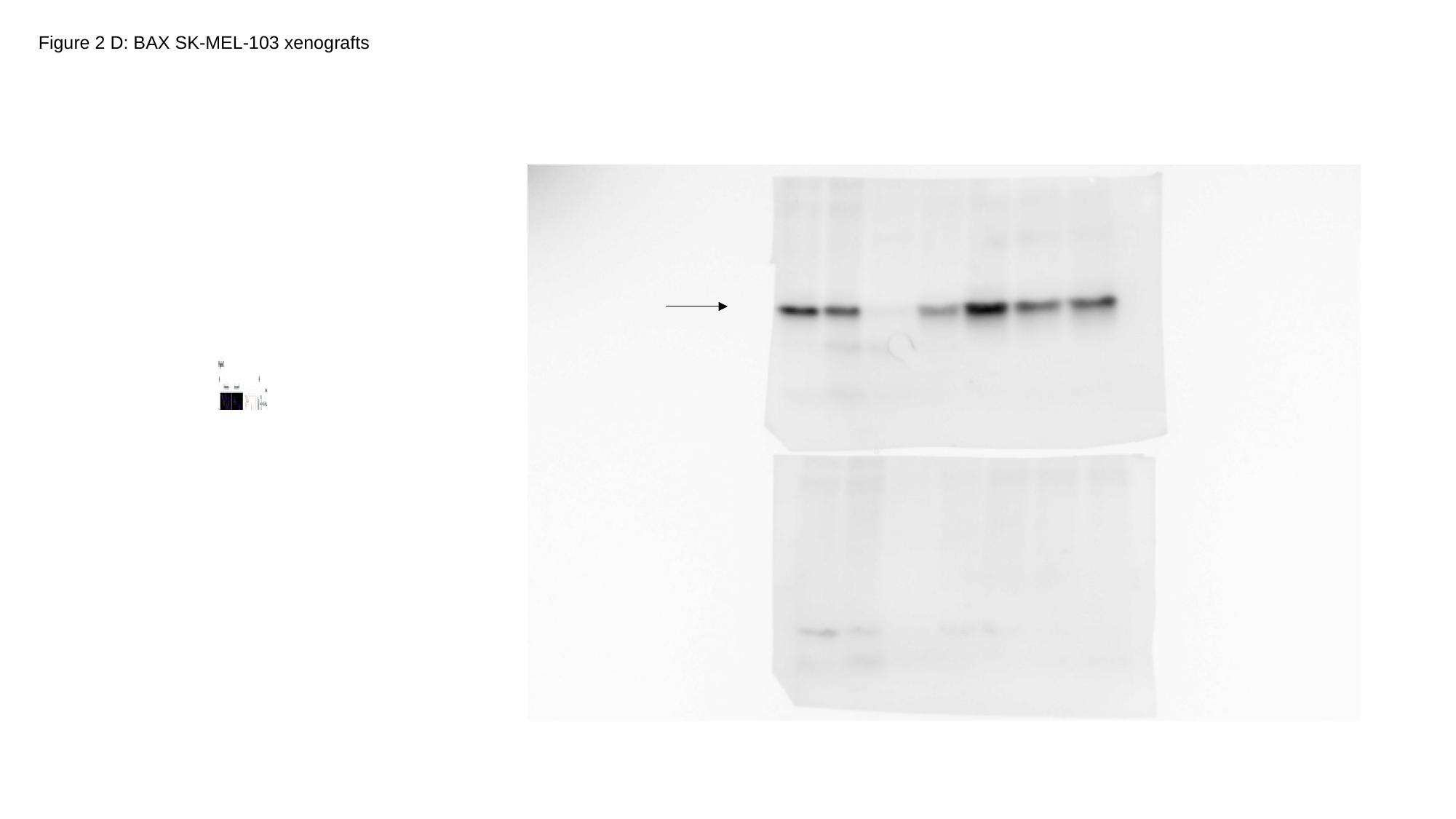

Figure 2 D: BAX SK-MEL-103 xenografts

## Slide 13
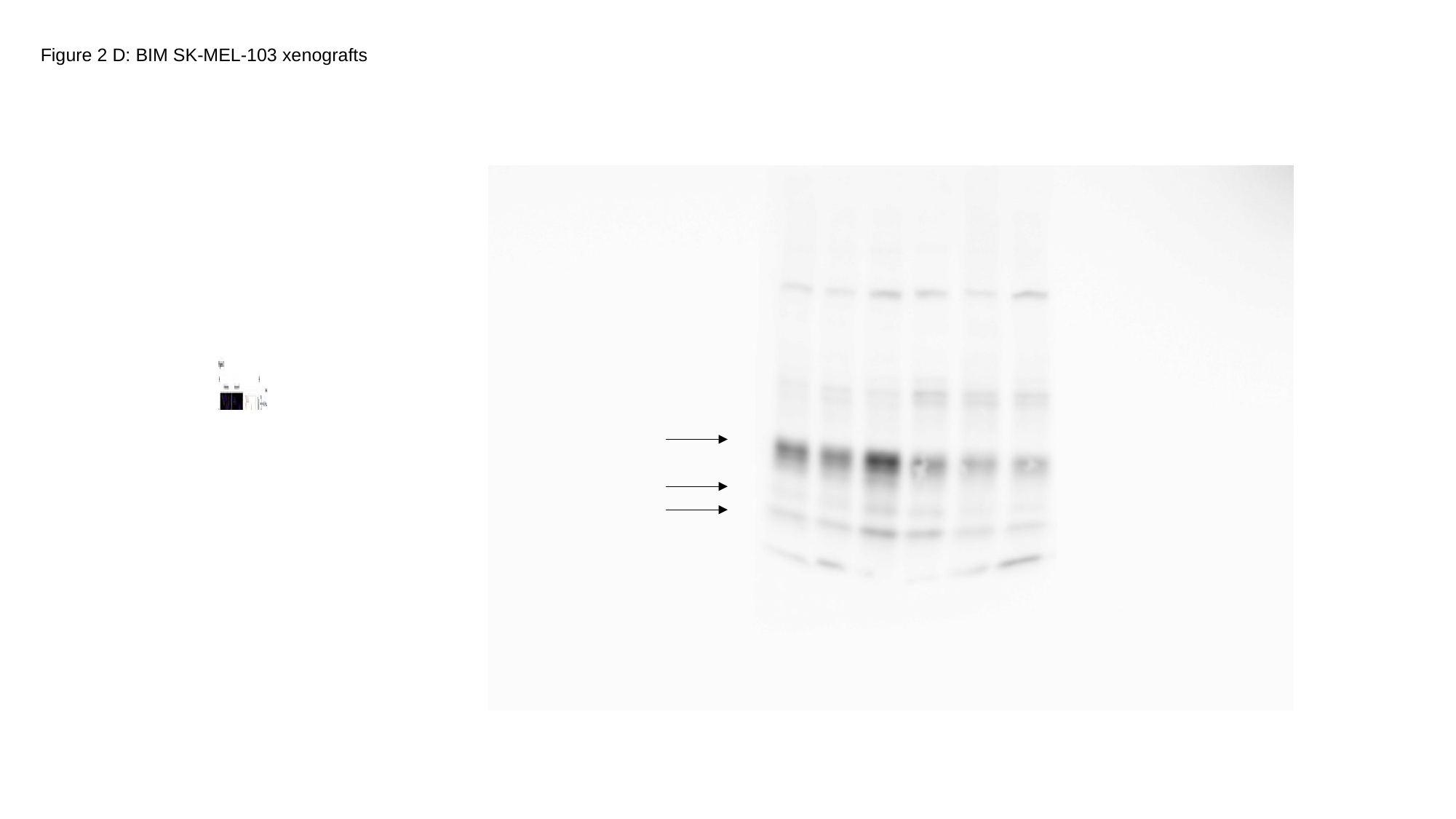

Figure 2 D: BIM SK-MEL-103 xenografts

## Slide 14
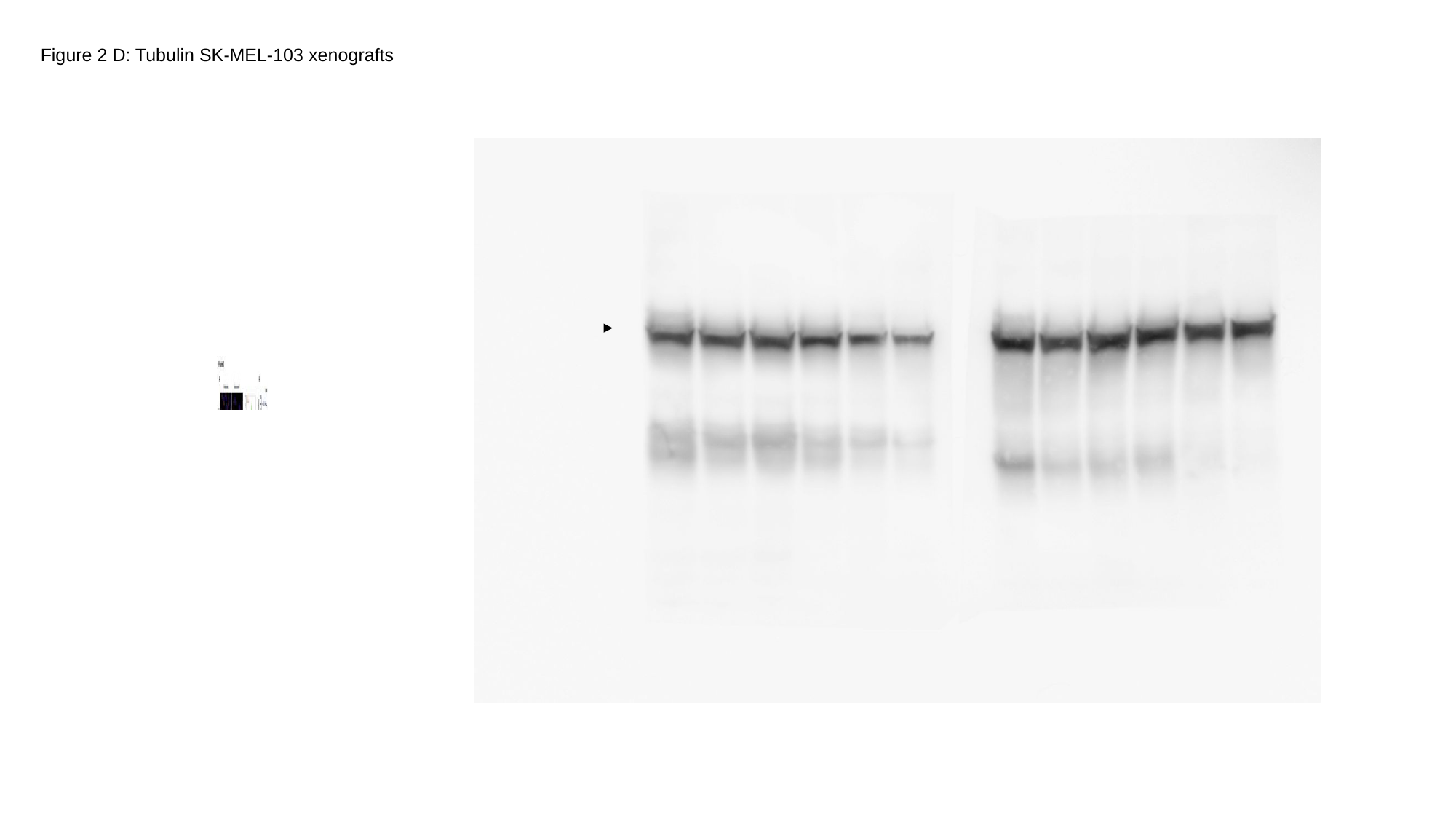

Figure 2 D: Tubulin SK-MEL-103 xenografts

## Slide 15
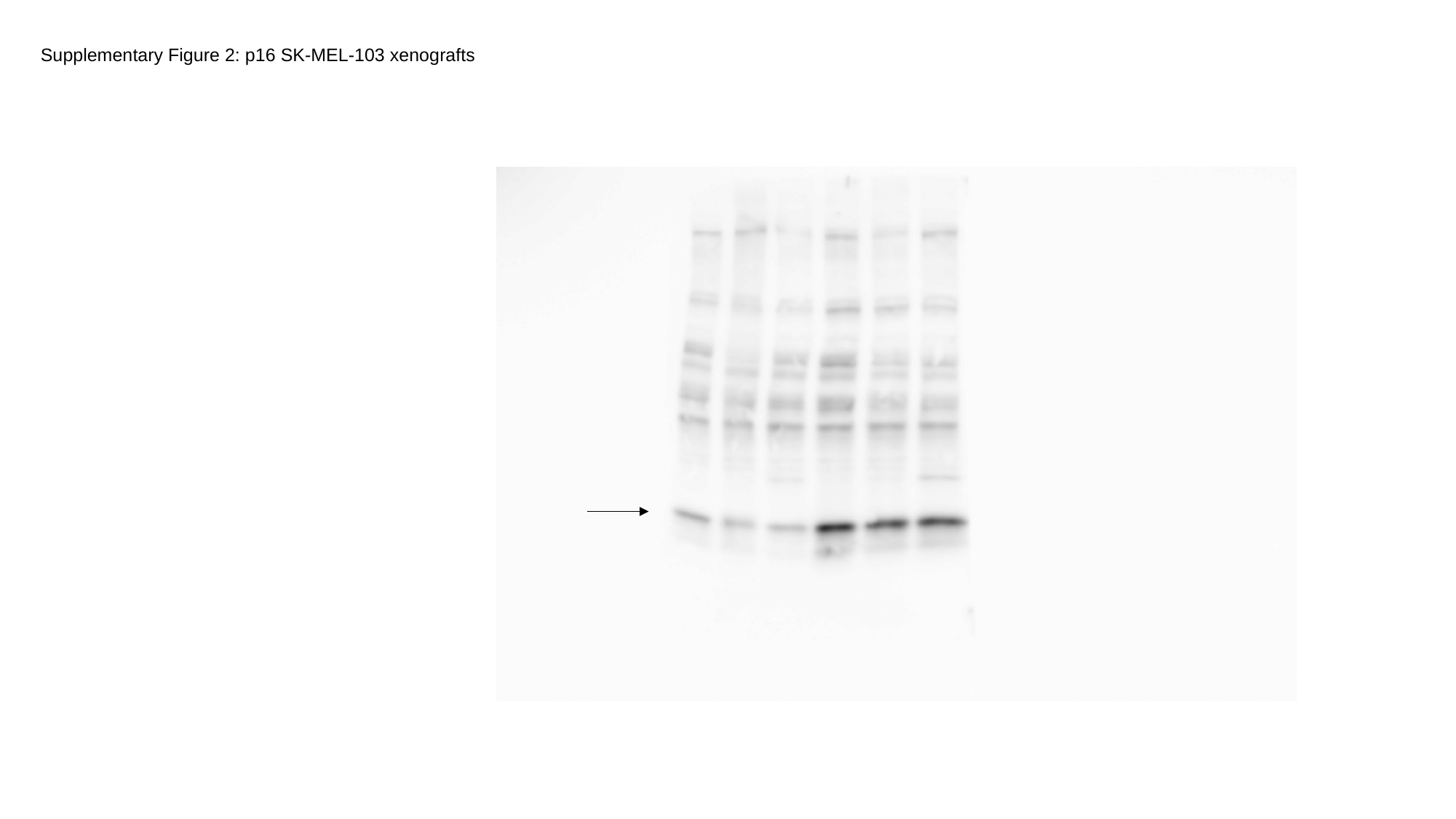

Supplementary Figure 2: p16 SK-MEL-103 xenografts

## Slide 16
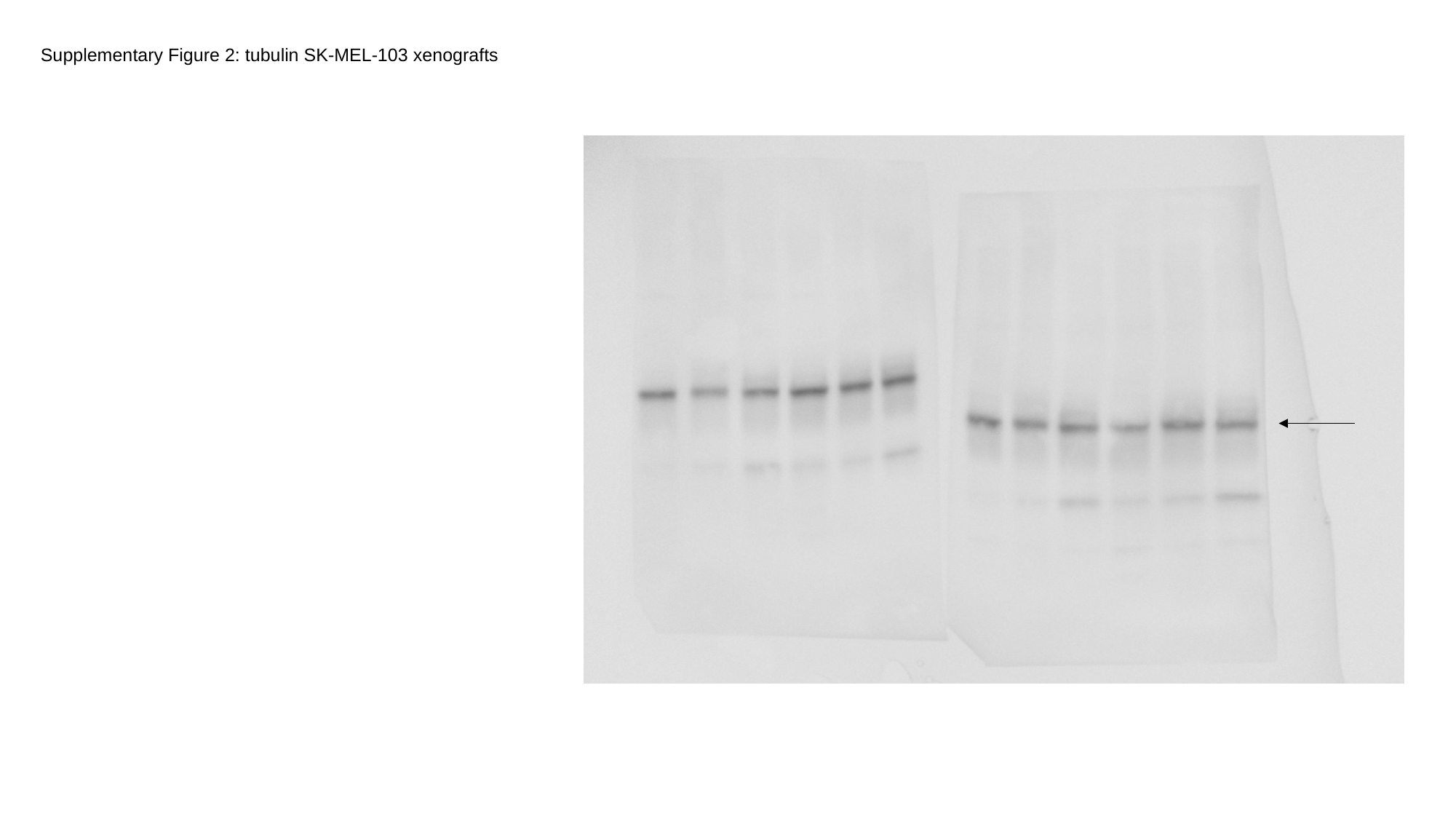

Supplementary Figure 2: tubulin SK-MEL-103 xenografts

## Slide 17
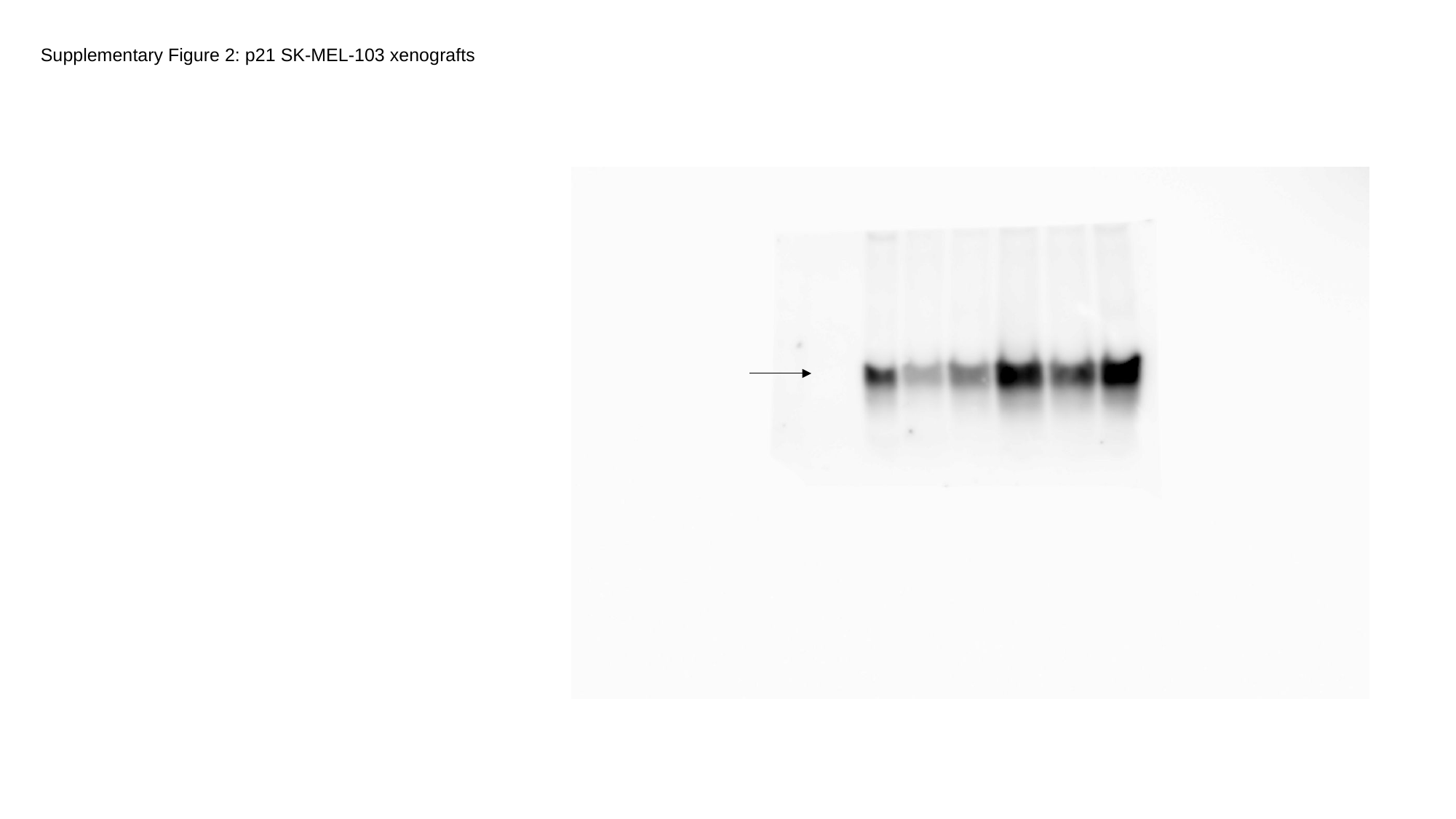

Supplementary Figure 2: p21 SK-MEL-103 xenografts

## Slide 18
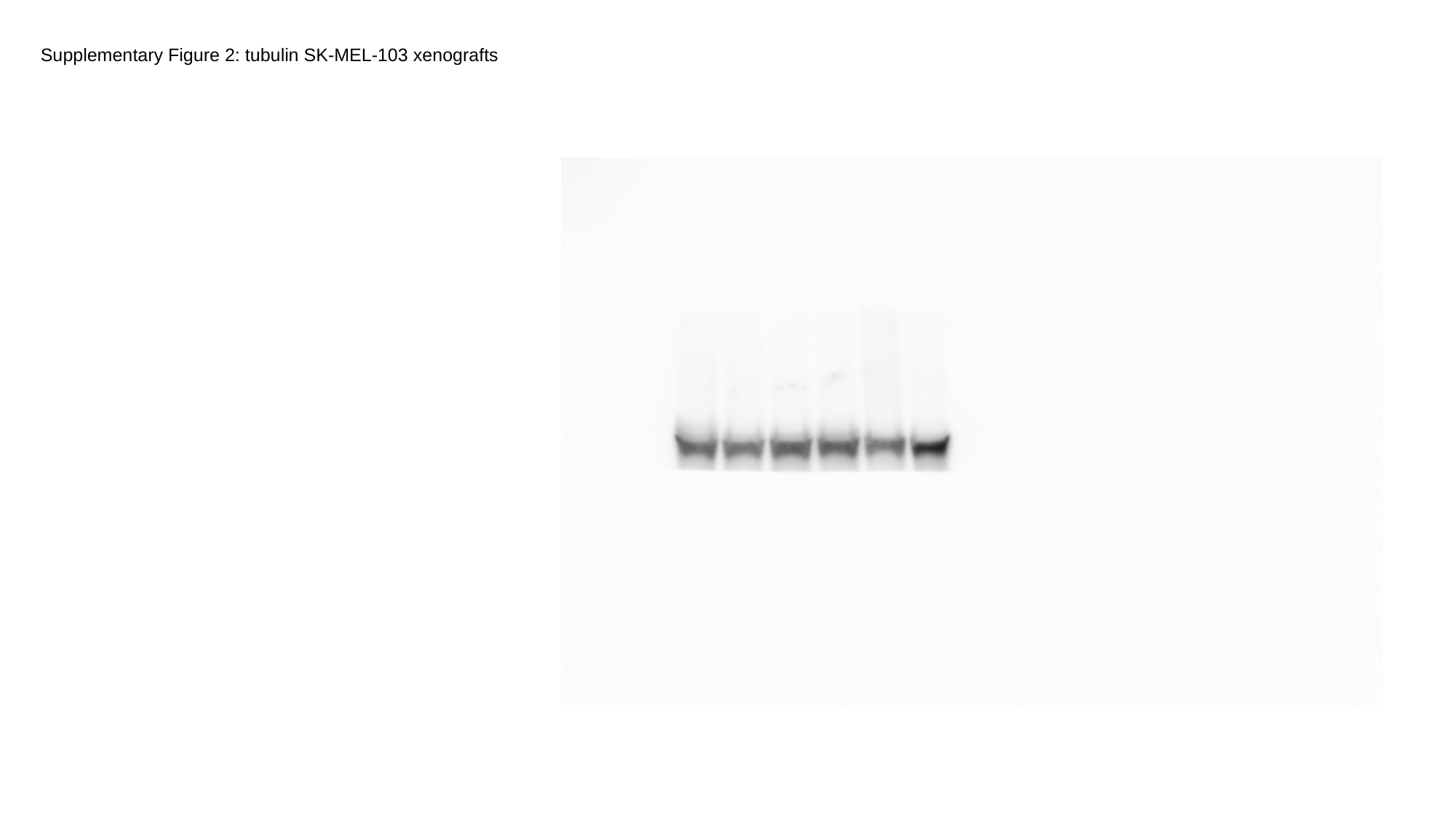

Supplementary Figure 2: tubulin SK-MEL-103 xenografts

## Slide 19
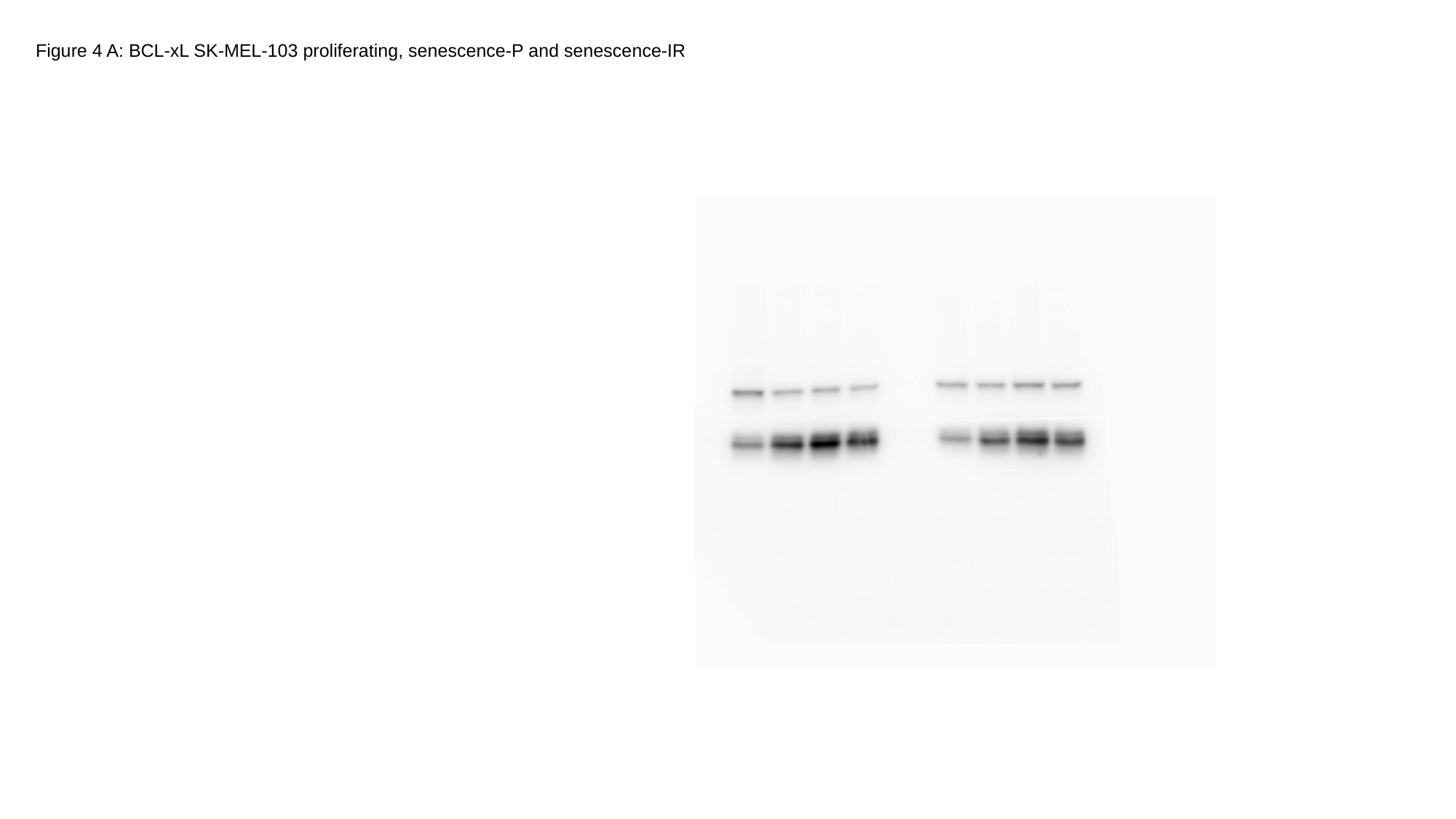

Figure 4 A: BCL-xL SK-MEL-103 proliferating, senescence-P and senescence-IR

## Slide 20
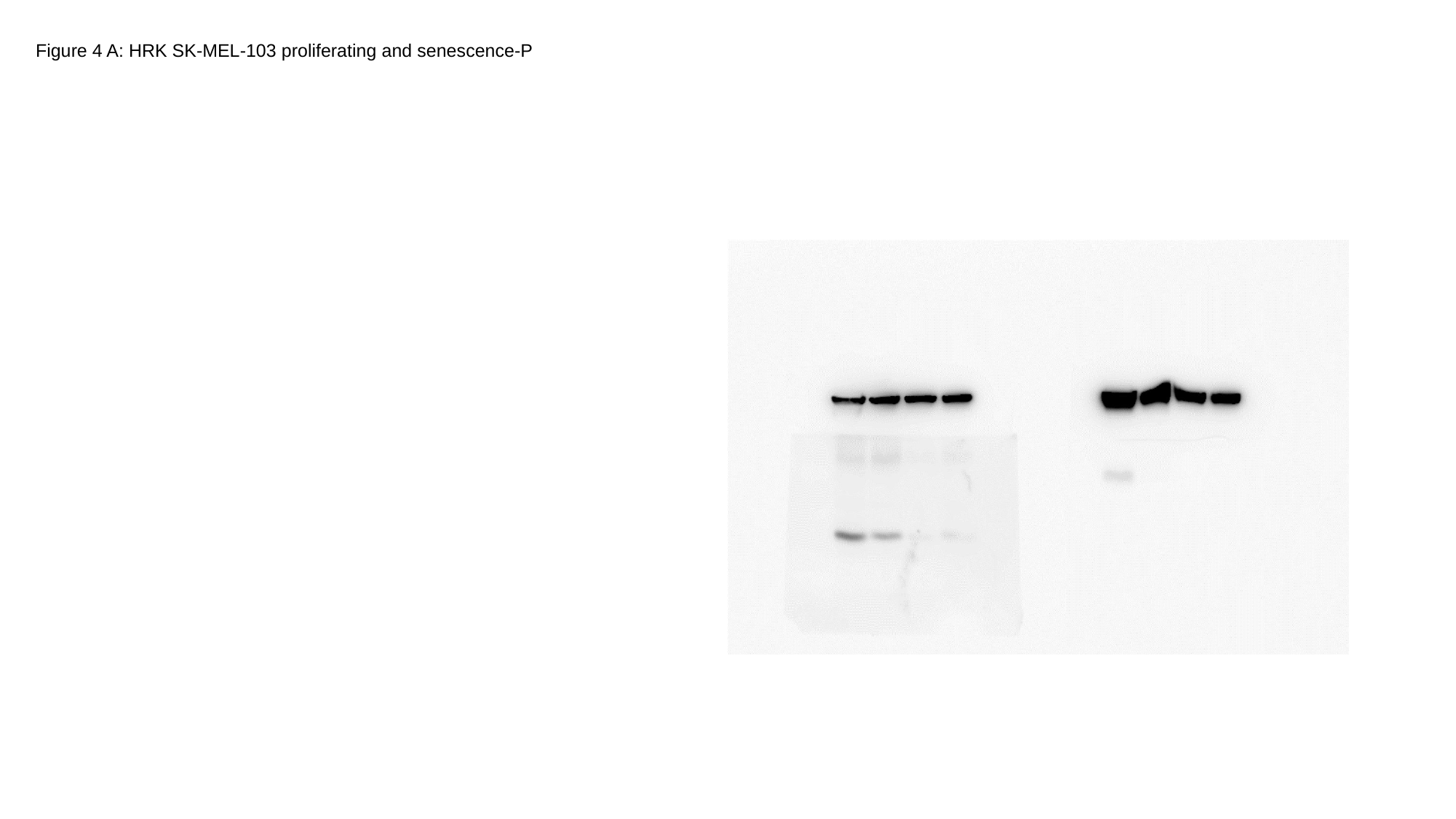

Figure 4 A: HRK SK-MEL-103 proliferating and senescence-P

## Slide 21
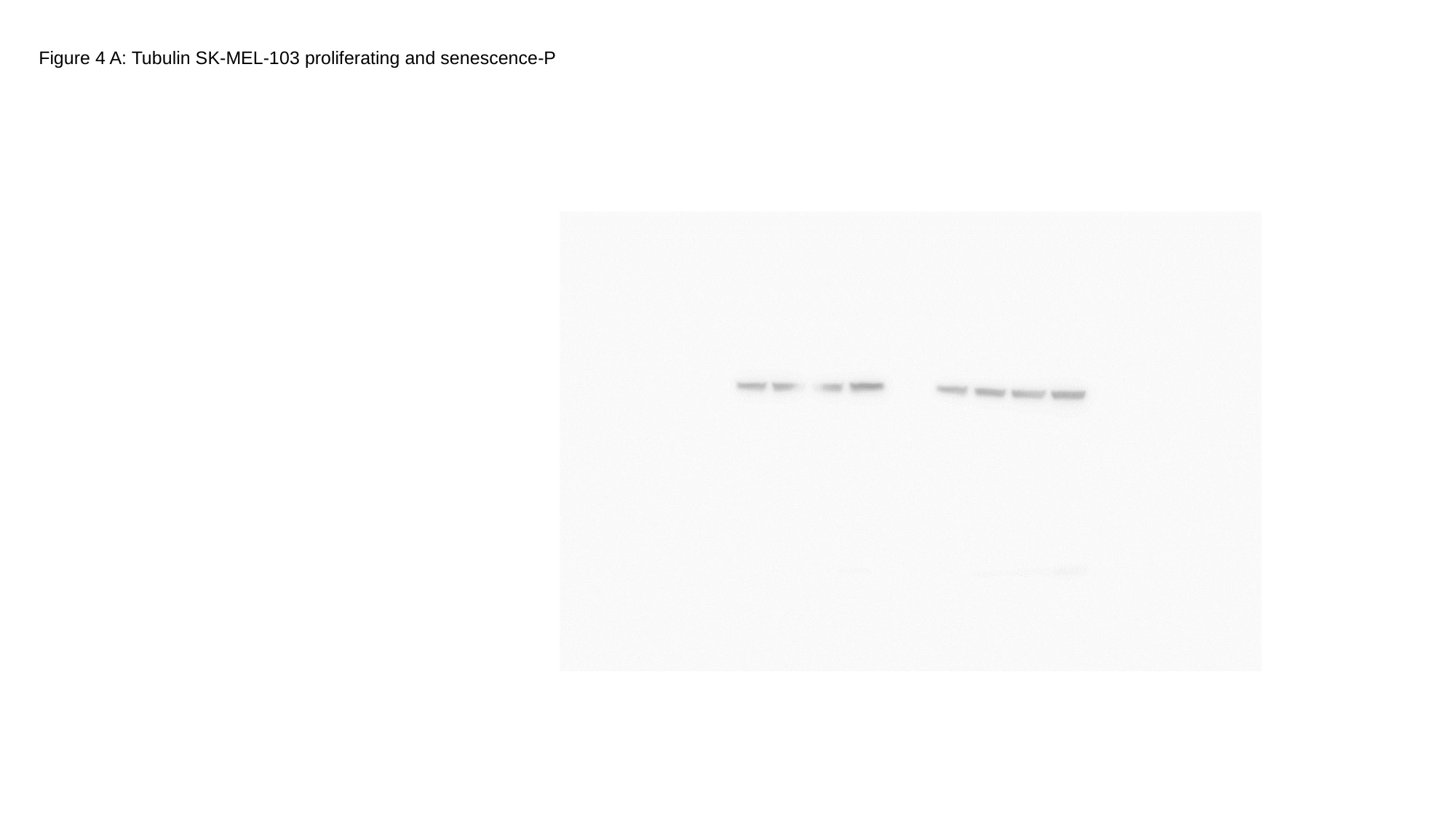

Figure 4 A: Tubulin SK-MEL-103 proliferating and senescence-P

## Slide 22
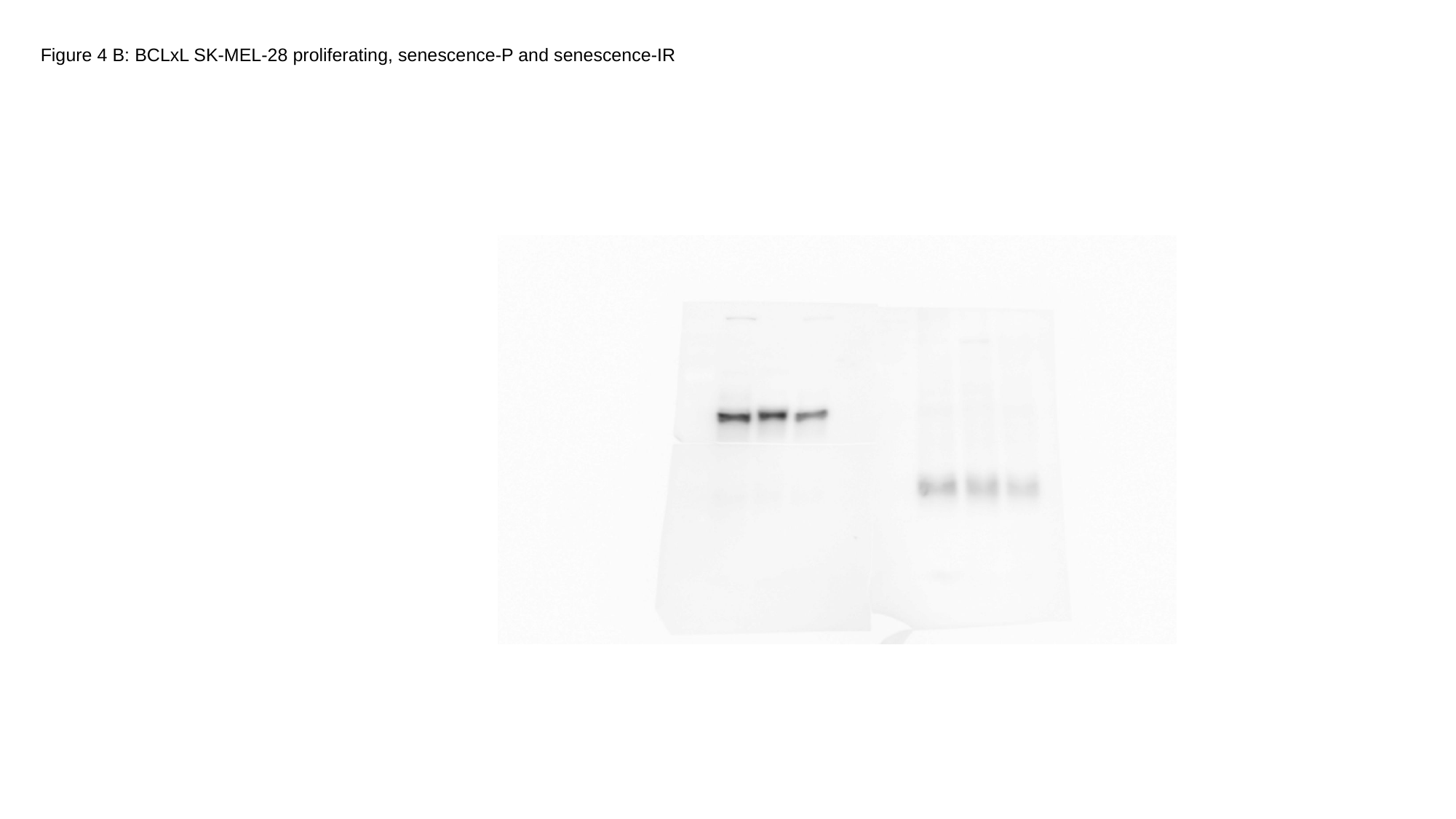

Figure 4 B: BCLxL SK-MEL-28 proliferating, senescence-P and senescence-IR

## Slide 23
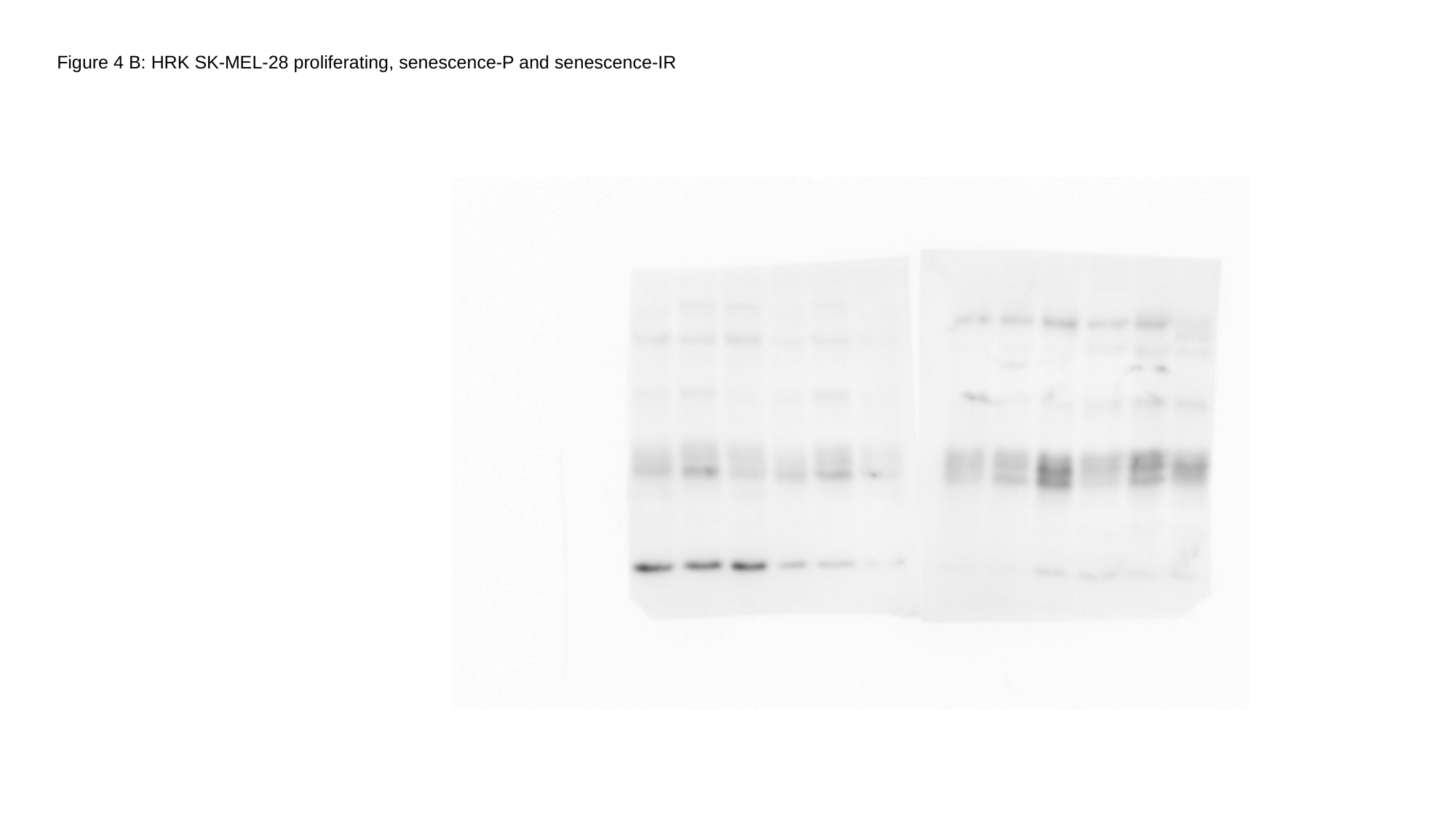

Figure 4 B: HRK SK-MEL-28 proliferating, senescence-P and senescence-IR

## Slide 24
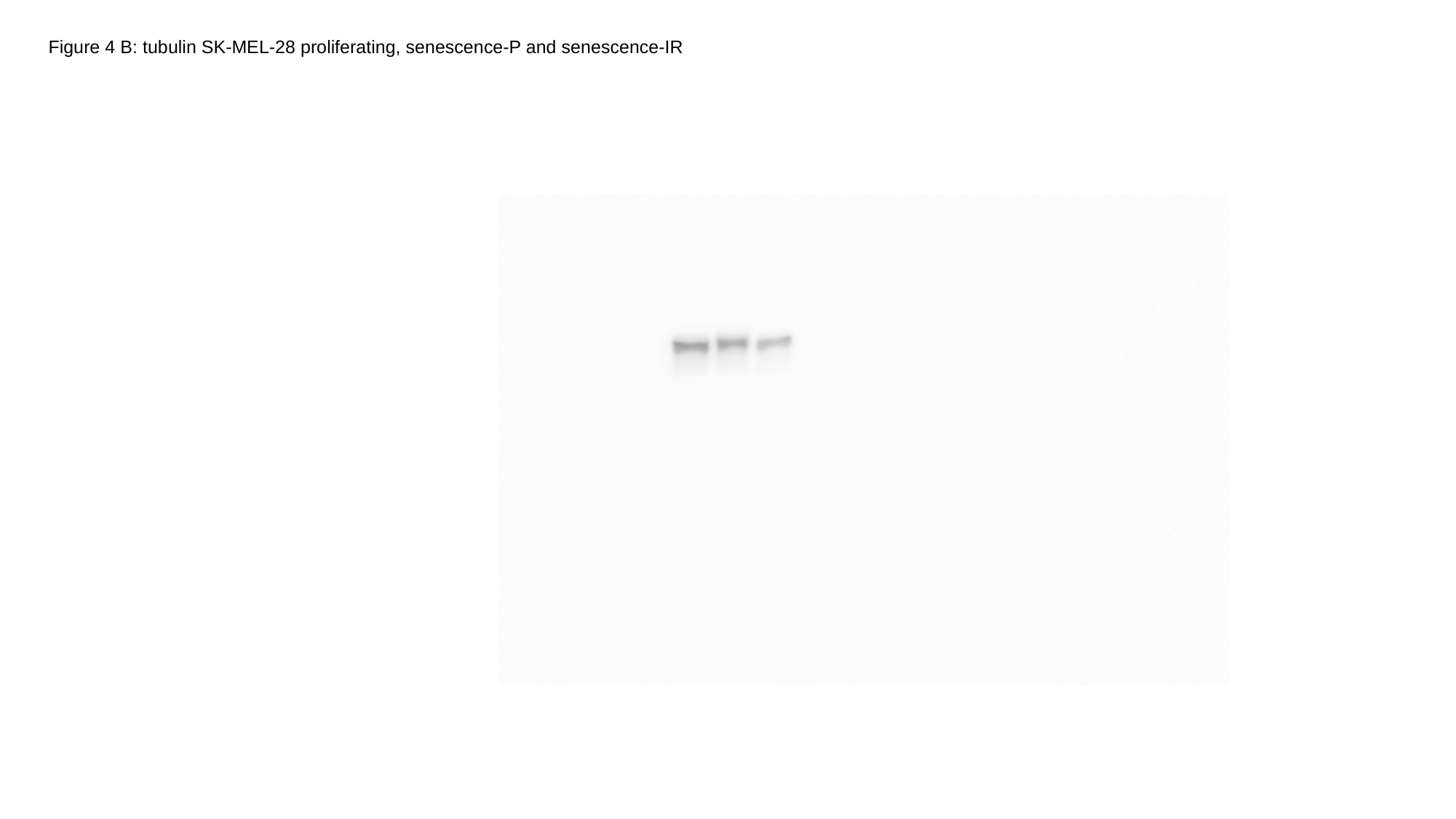

Figure 4 B: tubulin SK-MEL-28 proliferating, senescence-P and senescence-IR

## Slide 25
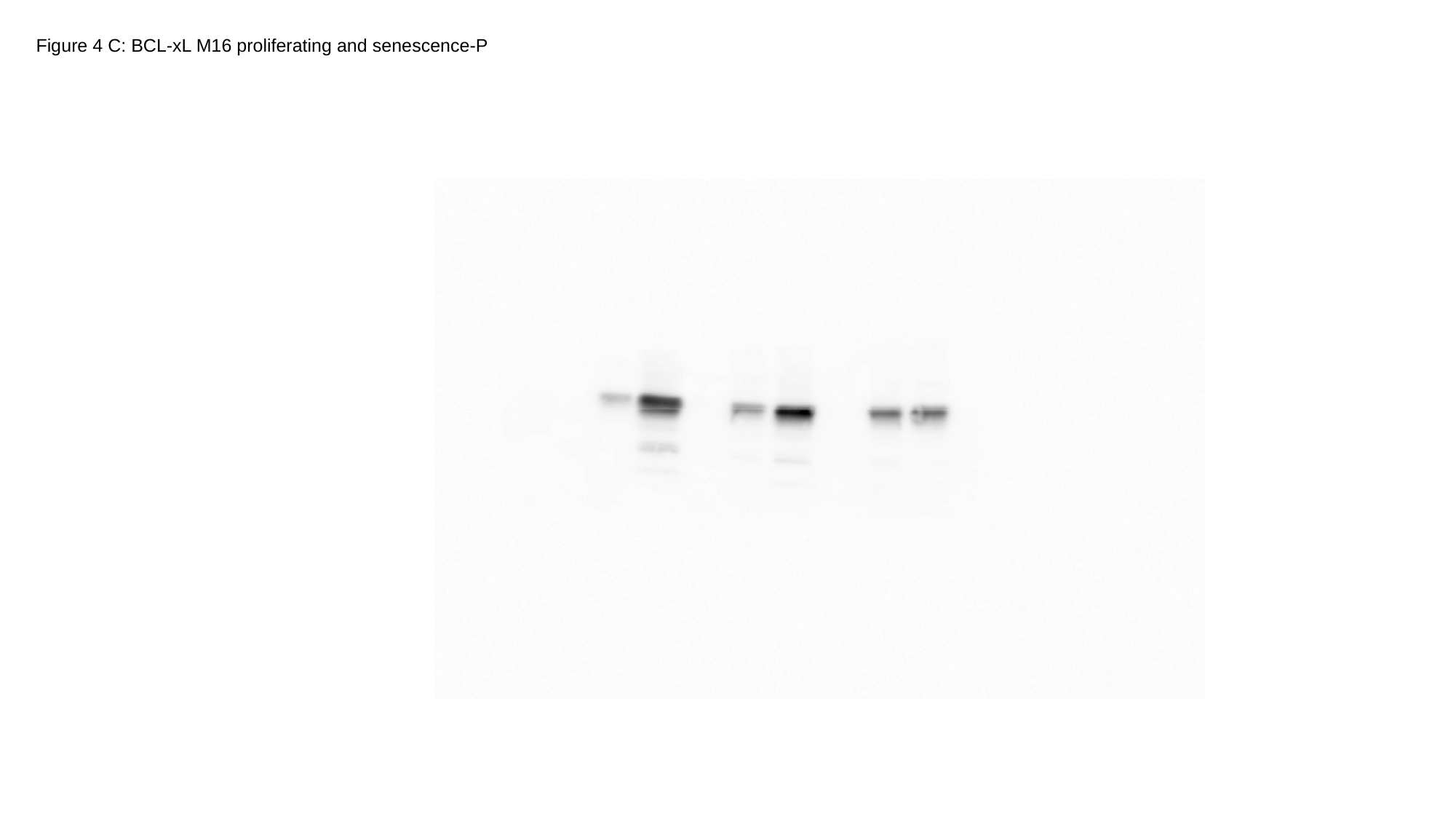

Figure 4 C: BCL-xL M16 proliferating and senescence-P

## Slide 26
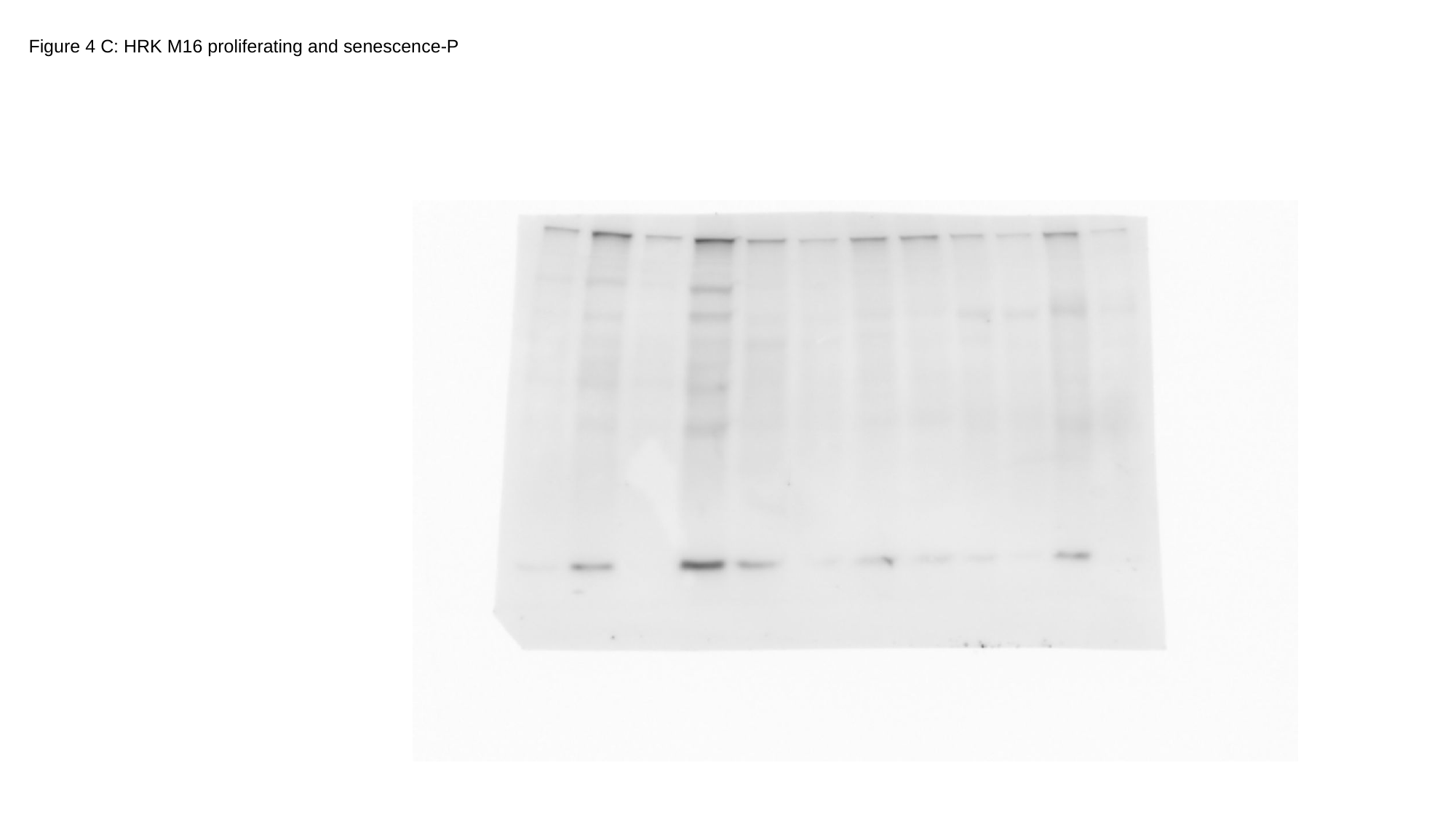

Figure 4 C: HRK M16 proliferating and senescence-P

## Slide 27
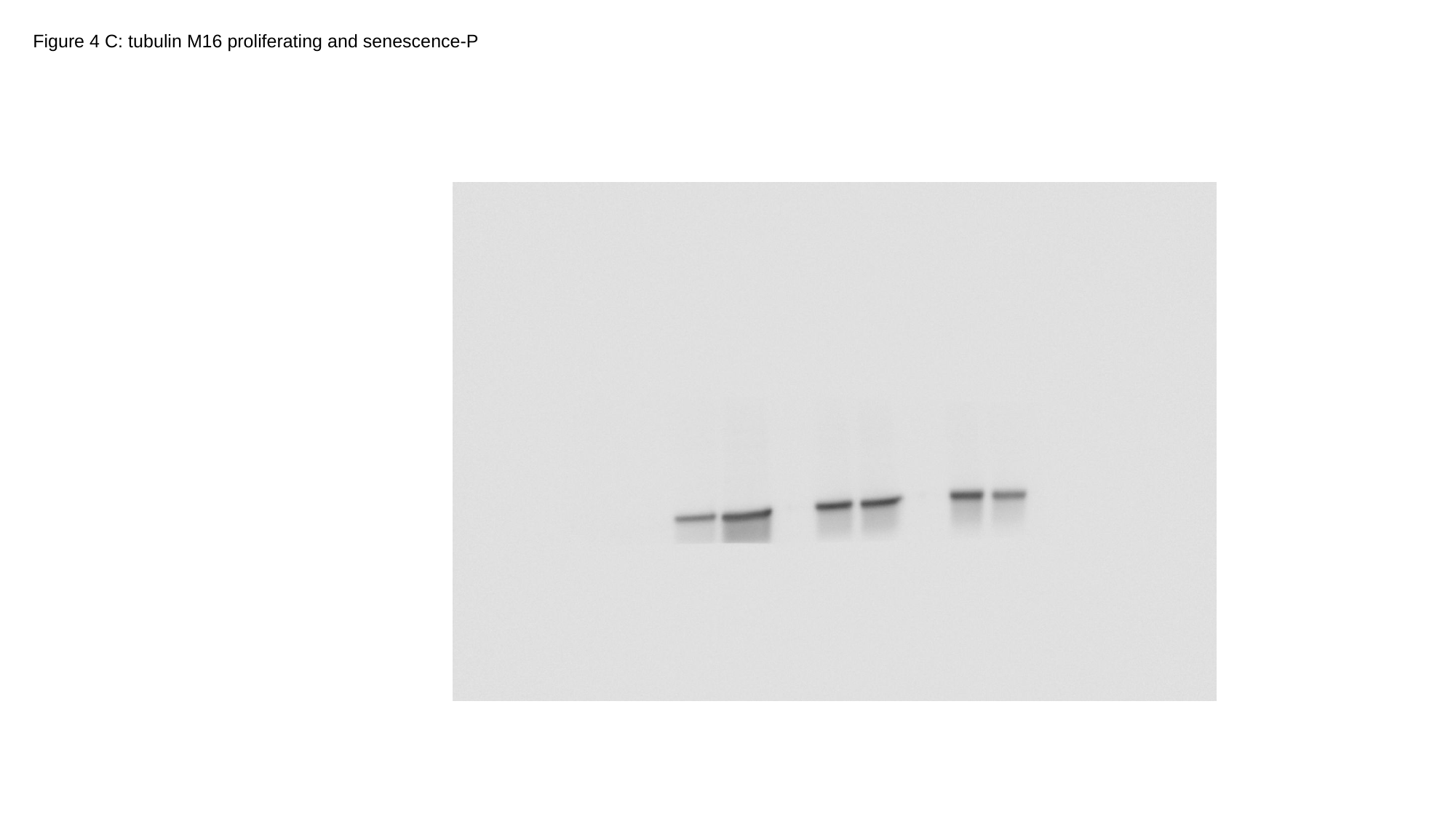

Figure 4 C: tubulin M16 proliferating and senescence-P

## Slide 28
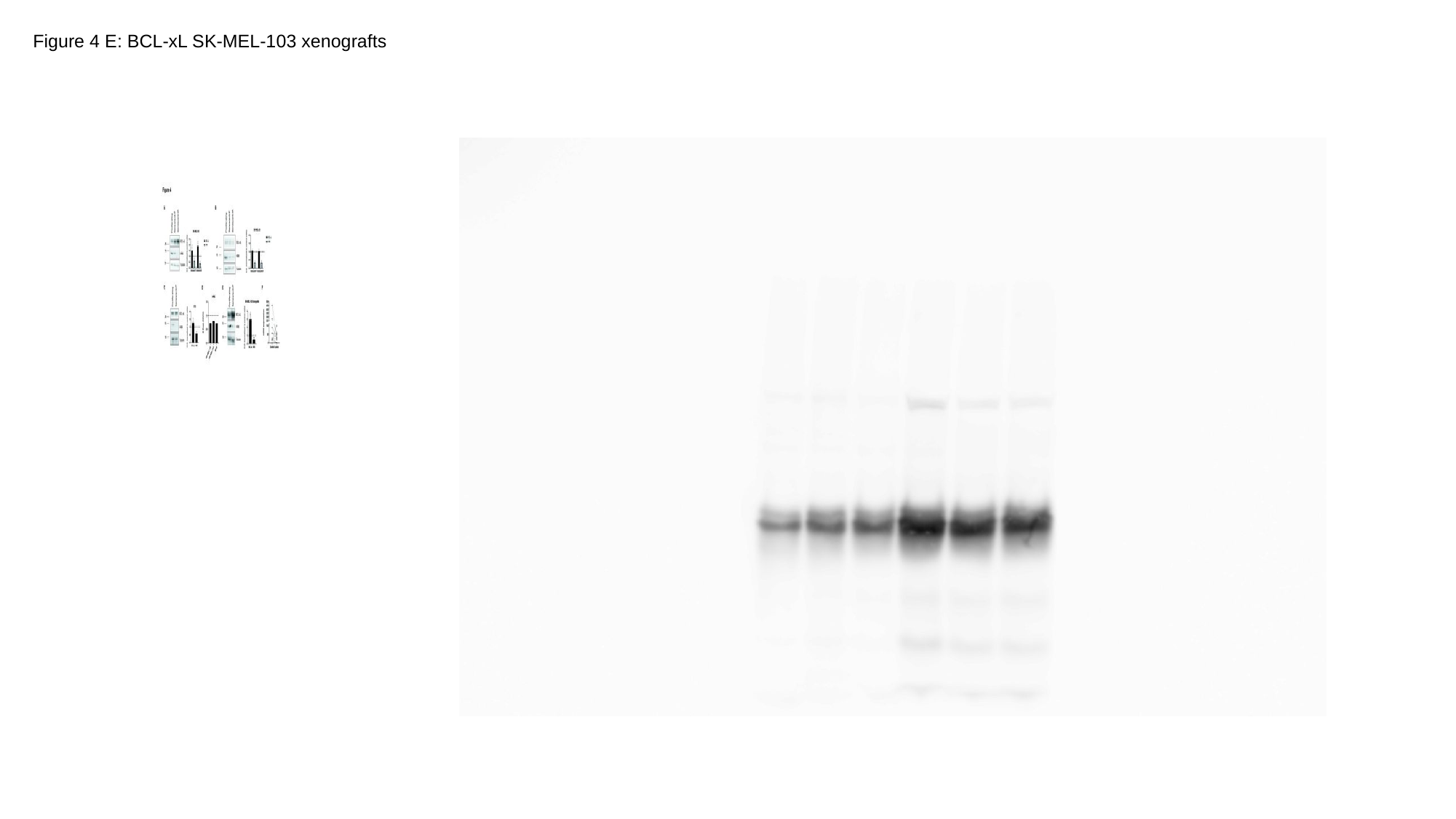

Figure 4 E: BCL-xL SK-MEL-103 xenografts

## Slide 29
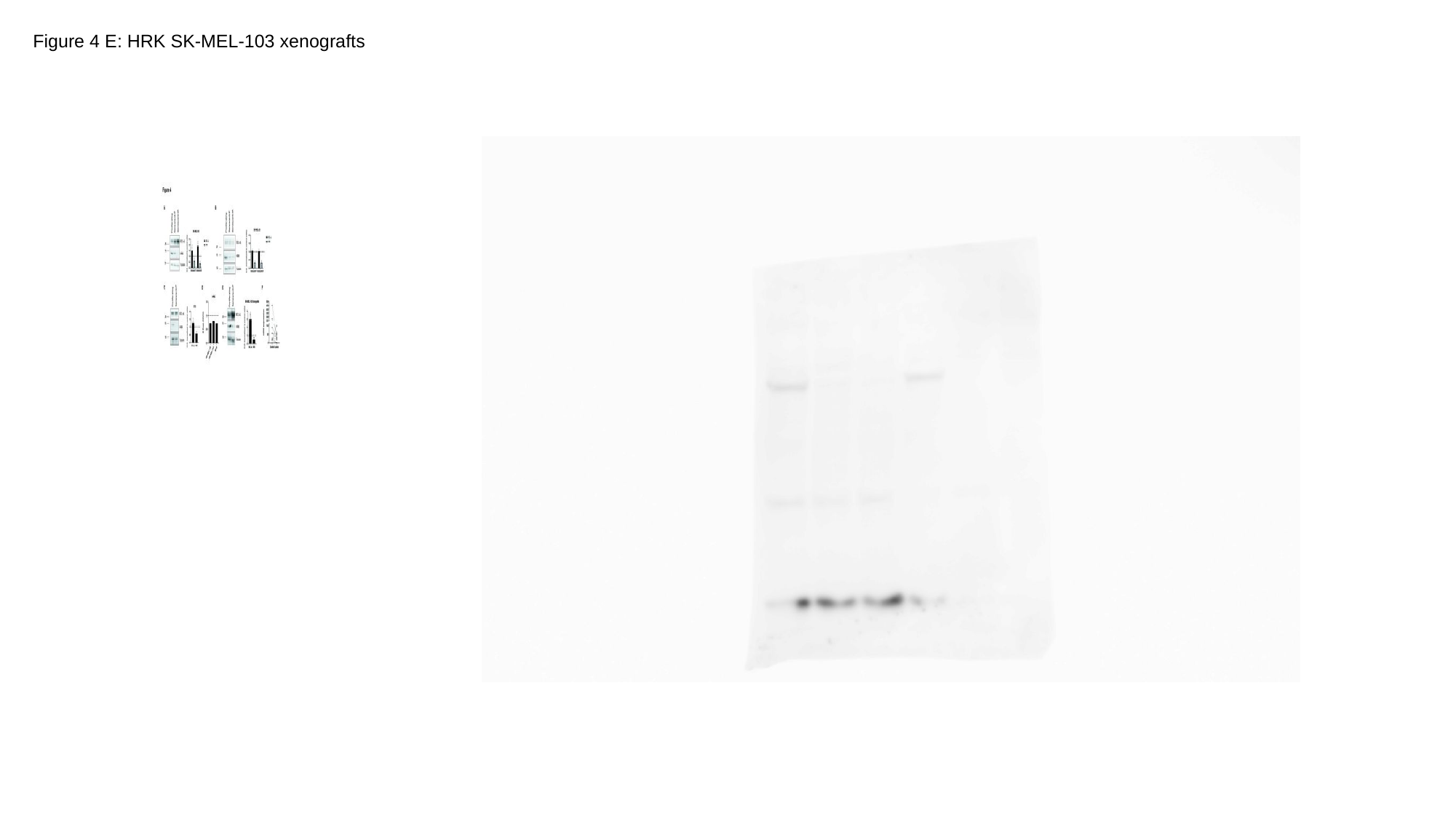

Figure 4 E: HRK SK-MEL-103 xenografts

## Slide 30
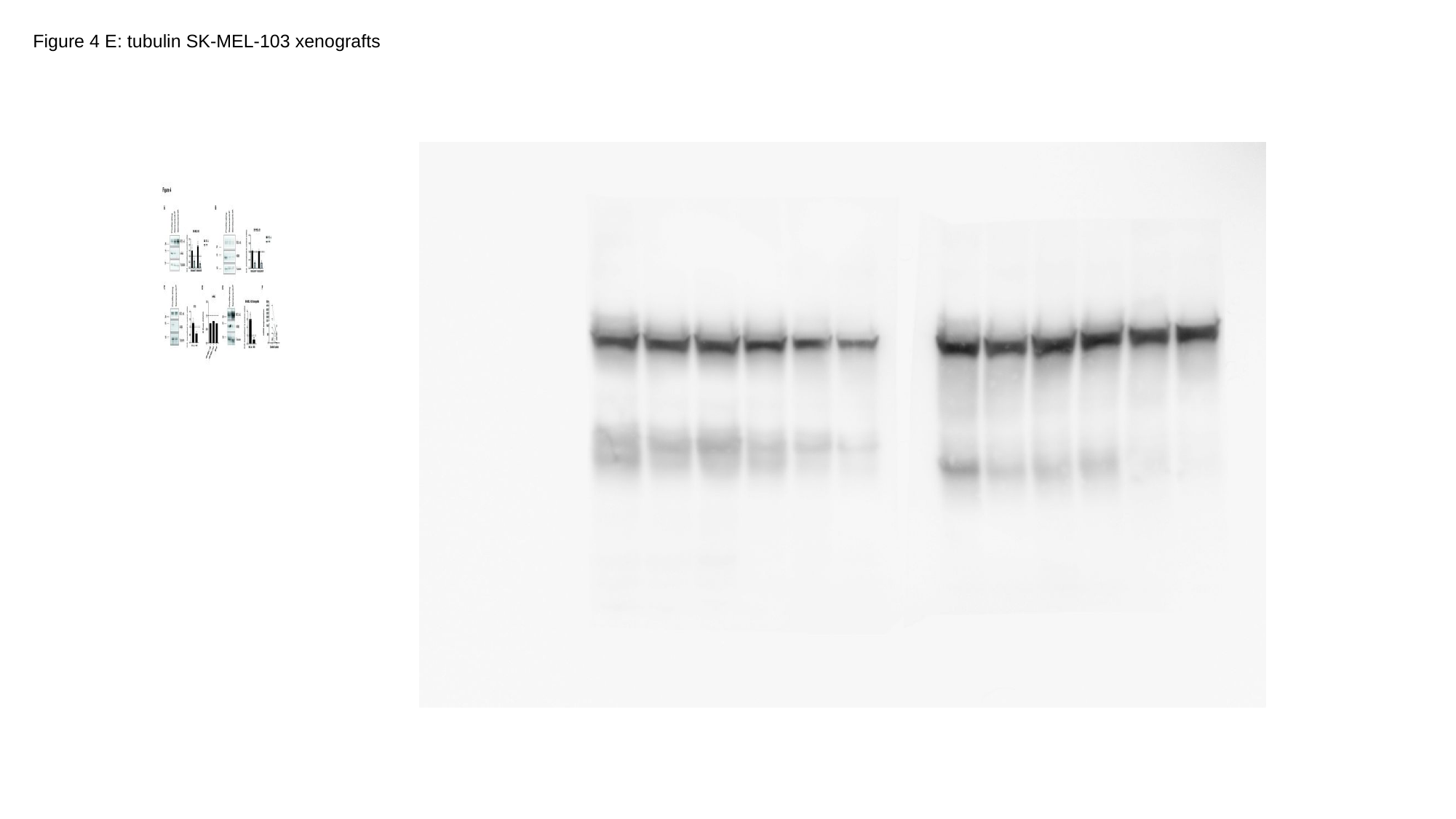

Figure 4 E: tubulin SK-MEL-103 xenografts

## Slide 31
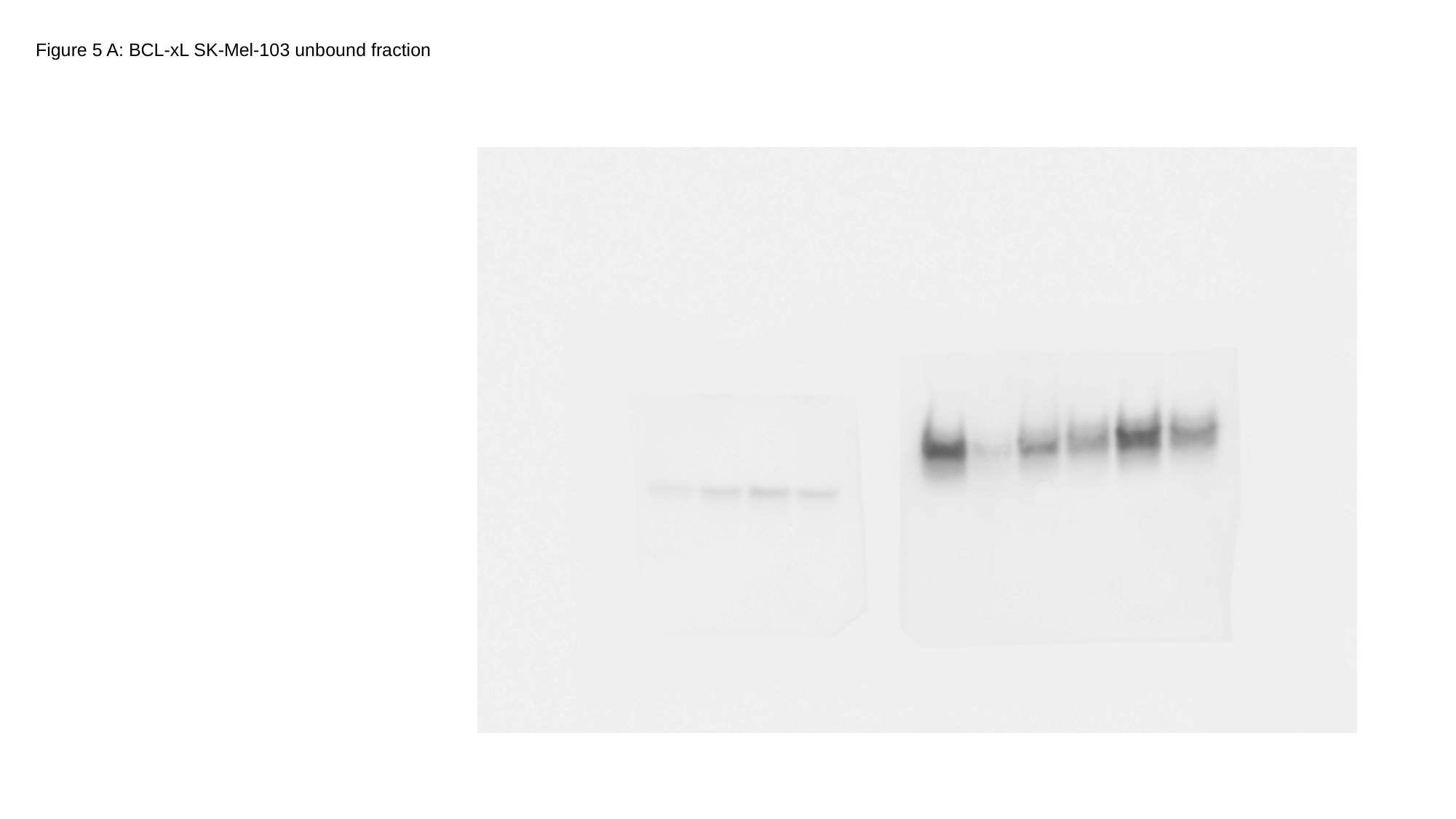

Figure 5 A: BCL-xL SK-Mel-103 unbound fraction

## Slide 32
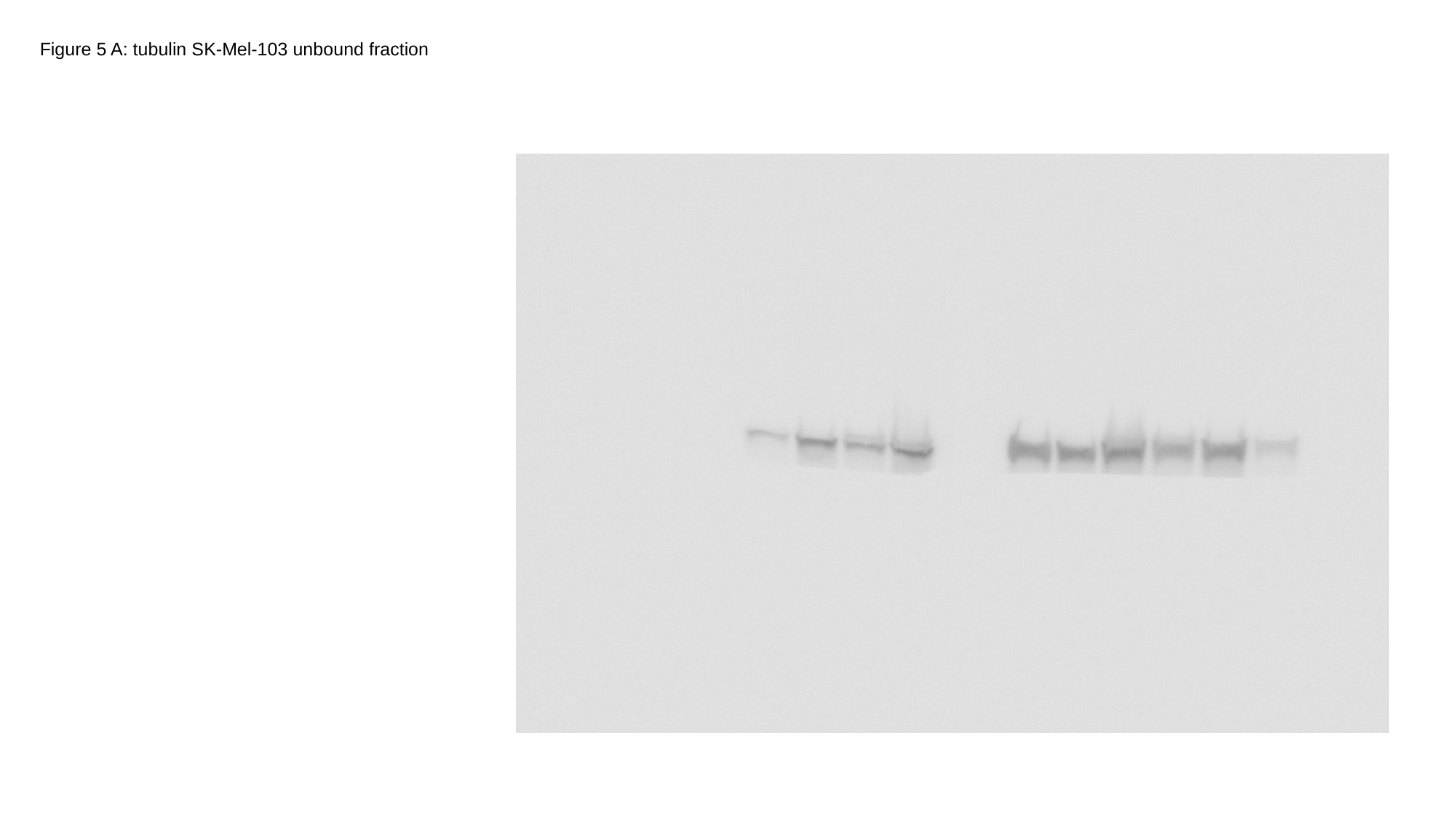

Figure 5 A: tubulin SK-Mel-103 unbound fraction

## Slide 33
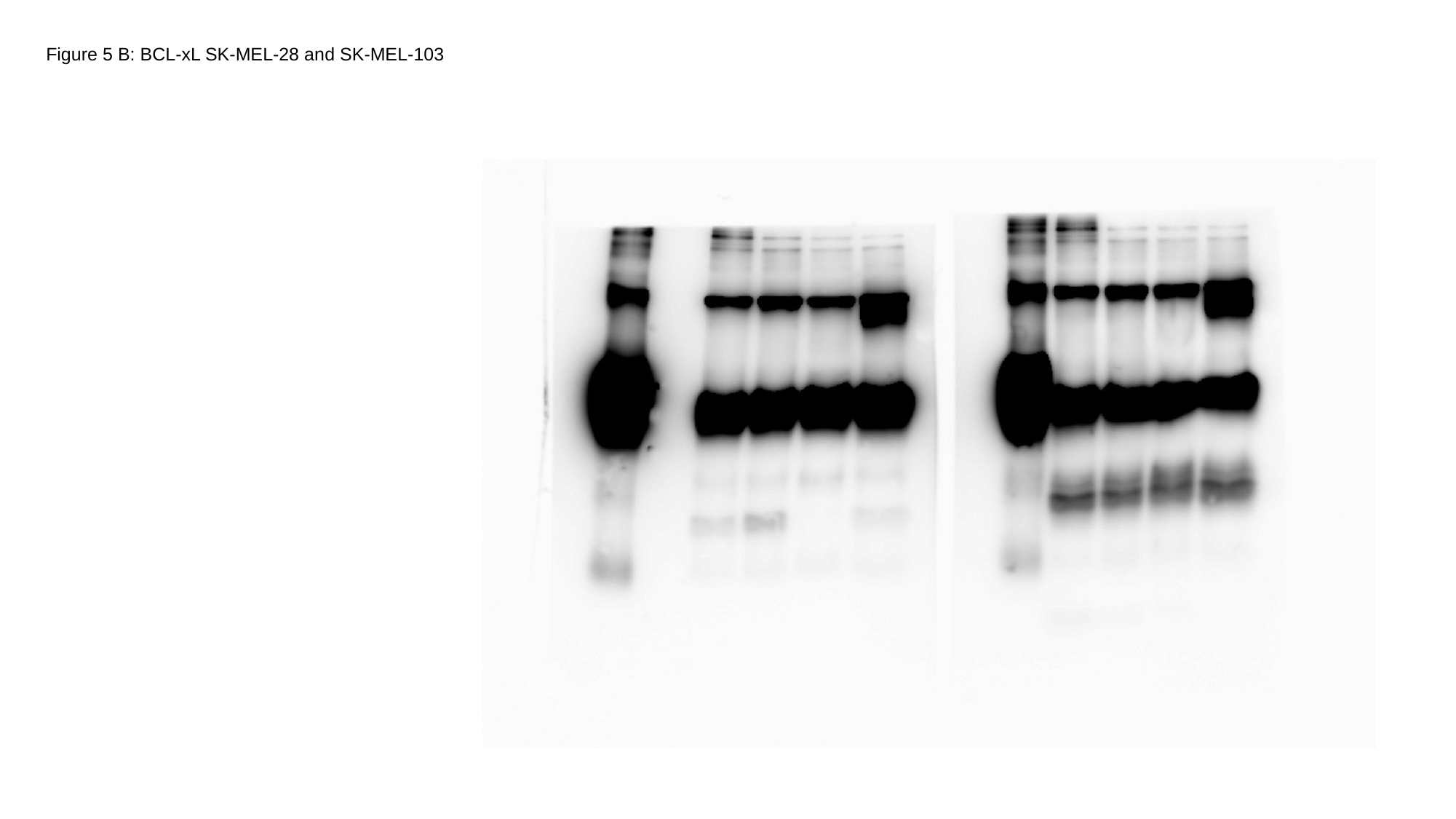

Figure 5 B: BCL-xL SK-MEL-28 and SK-MEL-103

## Slide 34
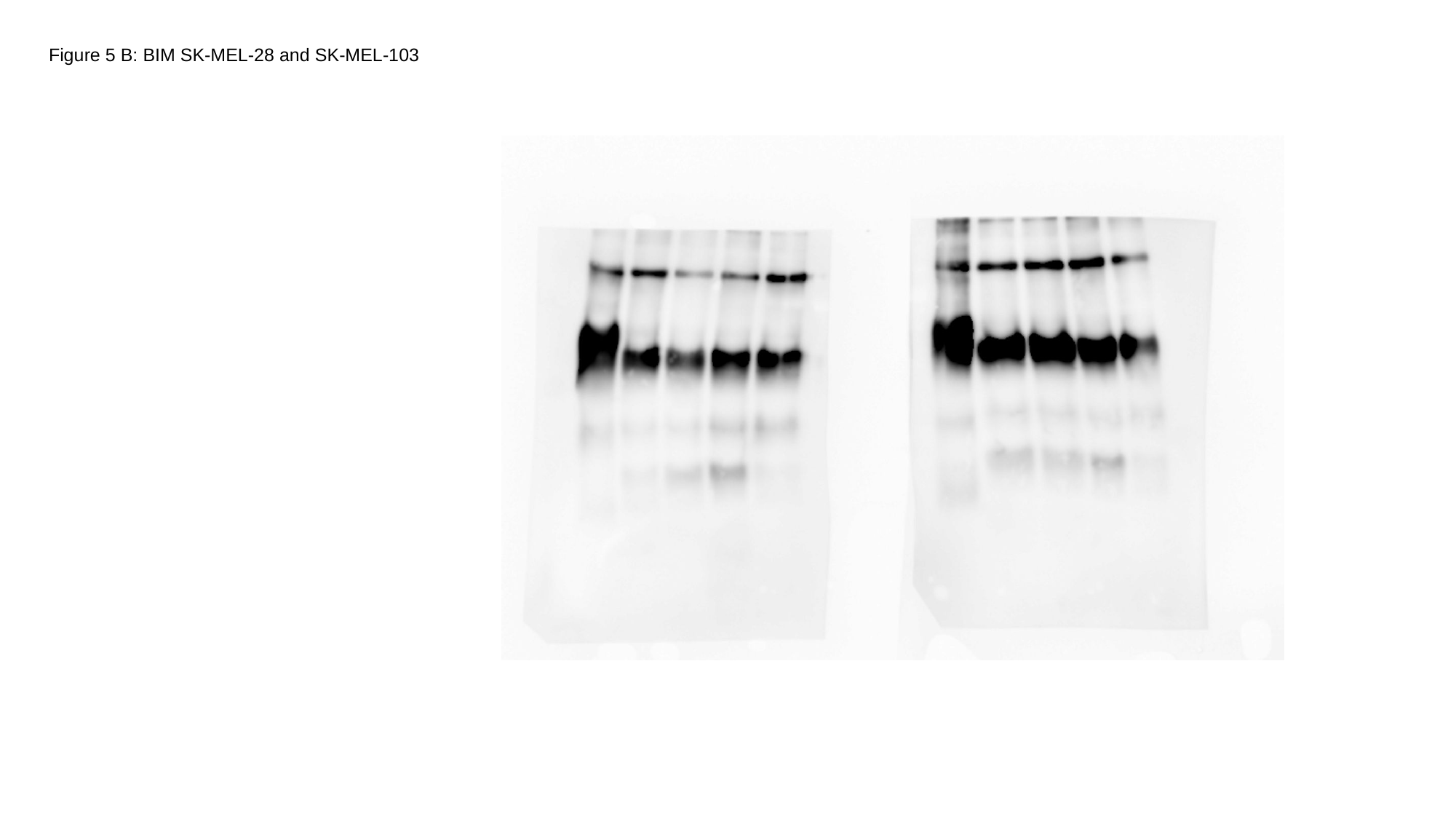

Figure 5 B: BIM SK-MEL-28 and SK-MEL-103

## Slide 35
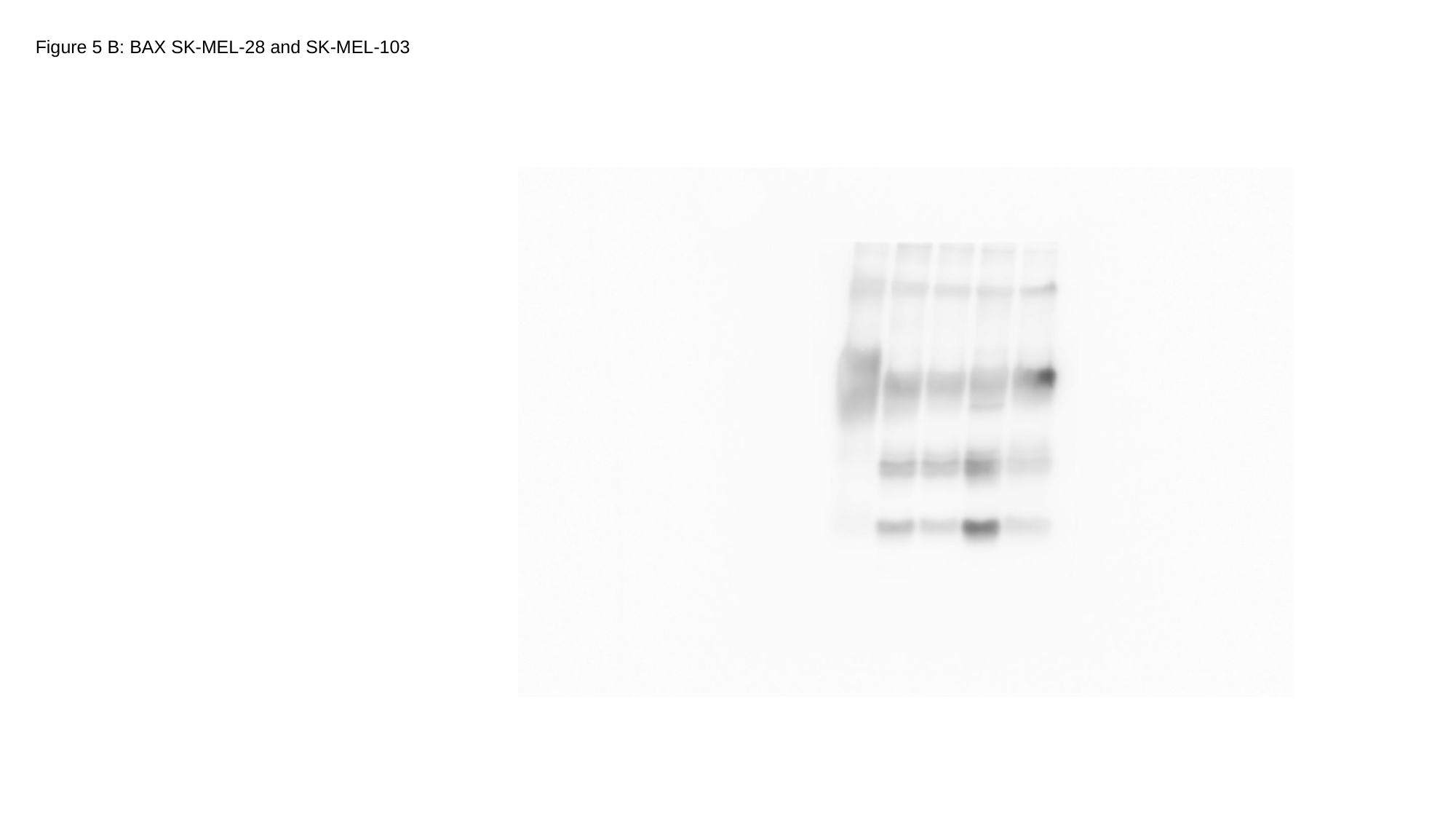

Figure 5 B: BAX SK-MEL-28 and SK-MEL-103

## Slide 36
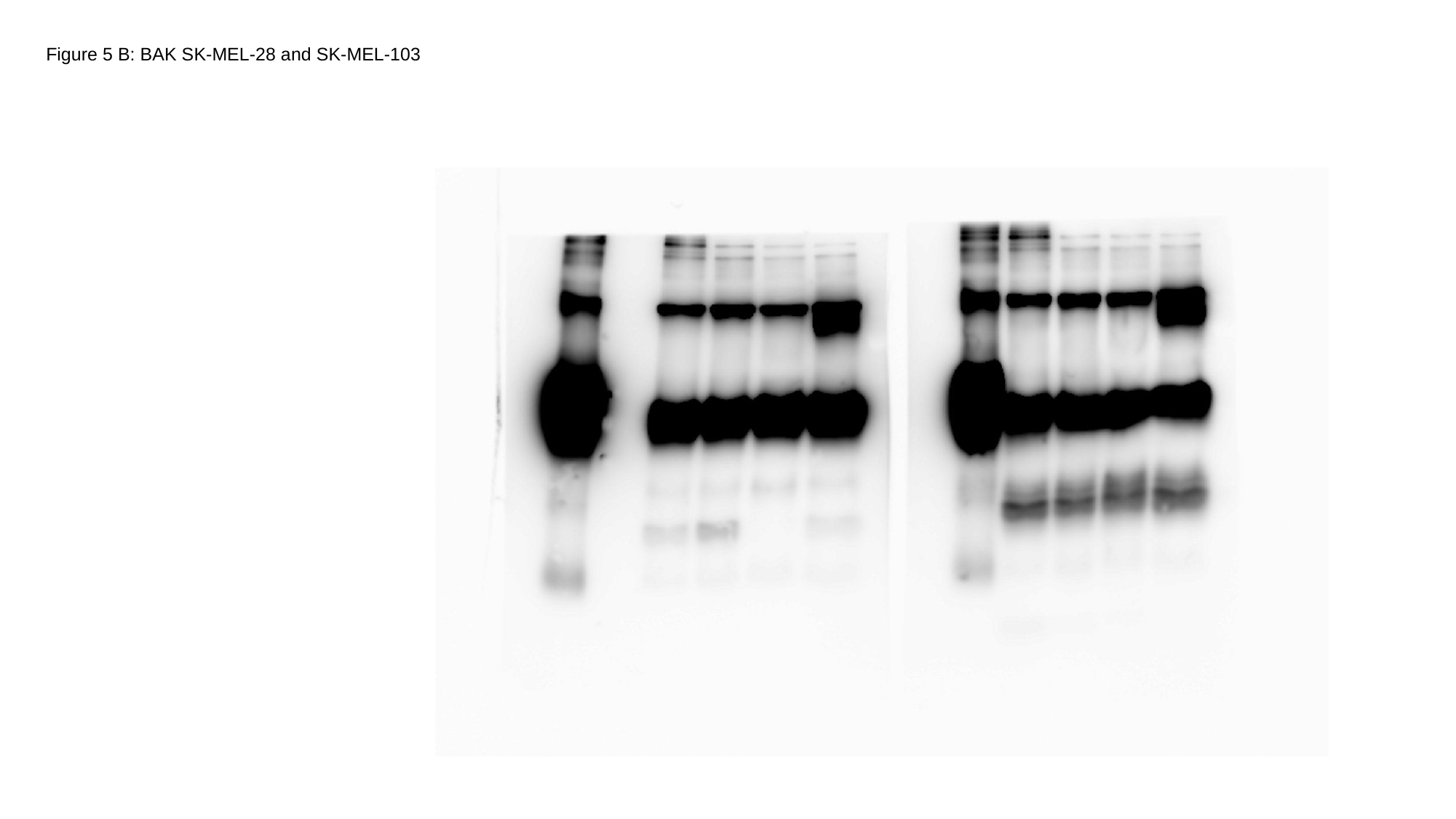

Figure 5 B: BAK SK-MEL-28 and SK-MEL-103
