# Supplementary material for: HRK downregulation and augmented BCL-xL binding to BAK confer apoptotic protection to therapy-induced senescent melanoma cells
Source: Cell Death Differ. 2024 Dec 3;32(4):646–56. doi: 10.1038/s41418-024-01417-z (PMC11982230; doi:10.1038/s41418-024-01417-z)
Supplement: Supplementary file 3 — Supplementary figure legend [file 41418_2024_1417_MOESM3_ESM.docx]

**SUPPLEMENTARY FIGURE LEGENDS**

**Supplementary Figure 1. Changes in protein levels in palbociclib-induced senescent SK-MEL-103 cells are due to changes in gene expression.** Z-score values for *BCL2L11* (BIM), *BAK1* (BAK) and *BAX* in proliferating and palbociclib-induced senescent (senescent-P) SK-MEL-103 cells.

**Supplementary Figure 2. Increase in the senescence markers p21 and p16 in SK-Mel-103 xenografts after palbociclib treatment.** Upper panel: Representative western blot images of protein extracts from SK-MEL-103 xenograft tumors of control or palbociclib treated mice. Lower panel: Optical density quantification of p21 and p16 normalized to tubulin. Values indicate mean values ± SEM from three samples. *p < 0.05 compared to control.

**Supplementary Figure 3. SA-β-galactosidase expression in irradiated-induced senescent melanoma cells.** A: Quantification of SA-β-galactosidase expression after 10Gy irradiation of melanoma cells by flow cytometry. B: Quantification of p21 expression after 10Gy irradiation of melanoma cells by flow cytometry. C: Quantification of p16 expression after 10Gy irradiation of melanoma cells by flow cytometry. Values indicate mean values ± SEM from at least three independent experiments. ** p<0.01, * p < 0.05 compared to control.

**Supplementary Figure 4. Increased trend of *CDKN1A* (p21) and *CDKN2A* (p16) mRNA expression in photodamaged skin.** mRNA expression of *CDKN1A* (p21) and *CDKN2A* (p16) in control and lesioned skin from healthy donors.

**Supplementary Figure 5. HRK downregulation trigger similar phenotypes as palbociclib-induced senescent melanoma cells.** A: Representative western blot images of SK-MEL-28 and SK-MEL-103 after transfection with siRNA against HRK or a control siRNA. B: % of cytochrome c retained inside the mitochondria with increasing concentrations of BIM peptide of SK-MEL-28 and SK-MEL-103 after transfection with siRNA against HRK or a control siRNA. C: % cytochrome c release after the incubation with sensitizer peptides (BAD 10µM, HRK 100µM and MS1 10µM) in SK-MEL-28 and SK-MEL-103 after transfection with siRNA against HRK or a control siRNA. D: Representative western blot images and optical density quantification of BCL-xL immunoprecipitation and BAK binding in SK-MEL-28 and SK-MEL-103 after transfection with siRNA against HRK or a control siRNA. Values indicate mean values ± SEM from at least three independent experiments.
